# Synthesis of ether lipids: natural compounds and analogues

**DOI:** 10.3762/bjoc.19.96

**Published:** 2023-09-08

**Authors:** Marco Antônio G B Gomes, Alicia Bauduin, Chloé Le Roux, Romain Fouinneteau, Wilfried Berthe, Mathieu Berchel, Hélène Couthon, Paul-Alain Jaffrès

**Affiliations:** 1 Univ. Brest, CNRS, CEMCA UMR 6521, 6 Avenue Victor Le Gorgeu, 29238 Brest, France

**Keywords:** amphiphiles, edelfosine, GAEL, glycerol lipids, glycolipids, ohmline, plasmalogen

## Abstract

Ether lipids are compounds present in many living organisms including humans that feature an ether bond linkage at the *sn*-1 position of the glycerol. This class of lipids features singular structural roles and biological functions. Alkyl ether lipids and alkenyl ether lipids (also identified as plasmalogens) correspond to the two sub-classes of naturally occurring ether lipids. In 1979 the discovery of the structure of the platelet-activating factor (PAF) that belongs to the alkyl ether class of lipids increased the interest in these bioactive lipids and further promoted the synthesis of non-natural ether lipids that was initiated in the late 60’s with the development of edelfosine (an anticancer drug). More recently, ohmline, a glyco glycero ether lipid that modulates selectively SK3 ion channels and reduces in vivo the occurrence of bone metastases, and other glyco glycero ether also identified as GAEL (glycosylated antitumor ether lipids) that exhibit promising anticancer properties renew the interest in this class of compounds. Indeed, ether lipid represent a new and promising class of compounds featuring the capacity to modulate selectively the activity of some membrane proteins or, for other compounds, feature antiproliferative properties via an original mechanism of action. The increasing interest in studying ether lipids for fundamental and applied researches invited to review the methodologies developed to prepare ether lipids. In this review we focus on the synthetic method used for the preparation of alkyl ether lipids either naturally occurring ether lipids (e.g., PAF) or synthetic derivatives that were developed to study their biological properties. The synthesis of neutral or charged ether lipids are reported with the aim to assemble in this review the most frequently used methodologies to prepare this specific class of compounds.

## Introduction

**Figure 1:**
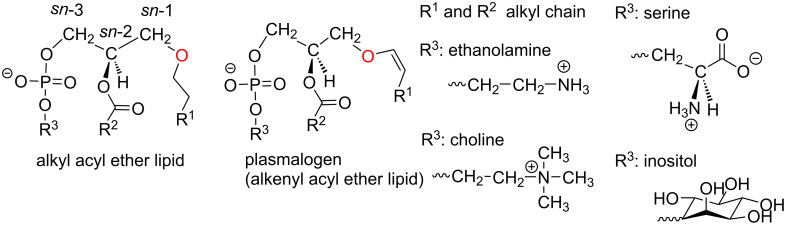
Chemical structure of some natural ether lipids (ELs).

Ether lipids (ELs) are natural compounds that feature a glycerol unit linked with an ether function to an alkyl (alkyl acyl ether lipid) or alkenyl (plasmalogen) lipid chain. For the alkenyl compounds, the vinyl ether function is characterized by a (Z)-configuration as shown in Figure 1. In addition, an acyl group is present on the secondary alcohol of the glycerol. This acyl group is constituted by a saturated or unsaturated lipid chain or, in the case of platelet-activating factor (PAF), by an acetyl group (R^2^ = CH_3_) [1]. The asymmetric carbon of the glycerol (*sn*-2 position) features a *R* configuration. The last substituent attached to the glycerol unit is a polar head group mostly constituted by a phosphatidylethanolamine group (PE) or a phosphocholine moiety (PC). ELs with either phosphatidylserine (PS) or phosphatidylinositol (PI) were also reported [2–3]. These two classes (alkyl and alkenyl) of tri-substituted glycerol ether lipids include a large number of compounds due to the possible structural variations at R^1^ (saturated and mono-unsaturated lipid chains) and R^2^ (a large variety of saturated, unsaturated and polyunsaturated lipid chains, Figure 1). These ether lipids constitute the majority of the ether lipids present in mammalians. Beside these two classes of compounds, there exists a multitude of other ether lipids that feature partly substituted glycerol. They correspond, for instance, to biosynthesized intermediates (e.g., 1-*O*-alkyl-glycerol-3-phosphate [4], lyso-PAF [5]) or neutral ether lipids (e.g., diacyl ether glycerol [6]). Ether glycerolipids are present in mammalian but also in anaerobic bacteria [7], archea (with an inverted stereochemistry at the *sn*-2 position of the glycerol) [8], protozoa [9], marine and land animals [10–11], but they are, according to the current scientific knowledge, absent in yeast [12] and plants [4].

It is estimated that ether lipids account for 10 to 20% of all the glycerophospholipids in humans. However, their tissue distribution is heterogeneous with abundant amount (up to 50% of the phosphoethanolamine based lipids) in central nervous system (mainly as PE-plasmalogen), in skeletal muscle, heart, kidney and lungs [13]. At a cellular scale, the biosynthesis of ELs is initiated in peroxisome and maturation is completed in the endoplasmic reticulum (ER). Then, ELs are spread off in the cell, to the plasma membranes but also in other biomembranes (membrane of mitochondria, ER, nucleus). The occurrence of ELs in tissues was first assessed by Snyder and Woods who reported that neutral ELs and phosphoglycero ELs were generally more abundant in cancer tissues than in normal human tissues [14]. It was also reported that the rate of neutral ether lipids in cancer cell line (quantified in vitro) was correlated to the tumorigenicity of the cancer cell lines in vivo [15]. However, the improvement of the analytical technics that now produces more accurate quantification of lipids with a distinction of neutral EL and phosphoglycero ELs produced contradictory data. Indeed, a recent study that focused on ether glycerophospholipids (alkyl and alkenyl ether lipids) indicates that in breast cancer cells (average of 9 breast cancer cell lines) the PC and PE ether lipids (alkyl and alkenyl together) were down regulated when compared to normal cells (MCF10A) with some variation in the different classes of lipid (PC, PE, PI, PS) [16]. However, alkylglyceronephosphate synthase (AGPS) is highly expressed in aggressive breast (231MFP), melanoma (C8161) and prostate cancer (PC3) cells compared with less aggressive cancer cells (MCF7, MUM2C, and LNCaP, respectively) suggesting that AGPS is an important player in the aggressiveness of cancers [17]. It was also suggested to use ELs as biomarkers for some human diseases like Parkinson disease [18], breast cancer [19] and in rectal adenocarcinoma [20] (in the last case by measuring lysophosphatidylcholine plasmalogen). Low levels of ether lipids are reported in inherited peroxisomal disorders (e.g., rhizomelic chondrodysplasia punctata or Zellweger syndrome) [21]. The severity of the phenotypes associated to peroxisomal disorders (e.g., brain abnormality, mental retardation, premature death) emphasizes the essential role of ether lipids in physiology of cells and tissues. This implication of ELs was also confirmed by producing glyceronephosphate *O*-acyltransferase (Gnpat) KO mouse which is a mouse model that stop the biosynthesis of ELs [22]. In that case, a reduction of the levels of various neurotransmitters were evidenced likely due to an alteration of the transport efficacy assumed by the synaptic vesicles. The phenotype of these KO mouse shows impaired social interactions and memory deficiency [23]. In another domain, it was reported that centenarians feature a specific profile of ELs in plasma with, in particular, higher level of *O*-alkyl form of phosphatidylcholine EL and a decreased level of phosphatidylethanolamine plasmalogen (alkenyl) [24]. It must be also noted that ELs are essential players of cell signaling [25]. Together, all these studies point out that ELs are essential for a multitude of biological functions [26] despite their mode of action is not yet fully understood.

From a molecular point of view, the ether function present in alkenyl ELs (plasmalogen) is highly sensitive to oxidation and it is reported that this function is even more reactive than unsaturated and polyunsaturated lipid chains [27]. Accordingly, plasmalogens could act as ROS scavenger and thus protect tissues (e.g., brain) from oxidative stress. The replacement of an ester bonding (present in diacyl glycero ether lipids) by an ether function has also some consequences on the biophysics of membranes. Two recent reviews by A. Koivuniemi [28] and by Jiménez-Rojo and Riezman [29] dedicated respectively to the effect of plasmalogen on biophysics of membranes and the molecular functions of ether lipids offer an interesting overview of the current knowledge of the effect of plasmalogen and ELs on membrane properties at a molecular scale. Shortly, according to molecular dynamics simulation, PE-plasmalogens form thicker, compressed and rigid bilayers when compared to PE-diacyl phosphoglycerolipids [30]. This is likely due to a reduction of the lateral area per molecule and an increase in lipid tail-ordering [31]. It must be however emphasis that the variability of the structure of the lipid chains can deeply influence the biophysical properties meaning that general conclusions could be modulated in function of the structure of these lipid chains. Another property of ELs is their capacity to favor inverted hexagonal phase and therefore to reduce the temperature of transition from L_α_ (lamellar-liquid crystalline phase) to H_II_ phases (inverted hexagonal phase). This property, which was mainly assessed for PE-ELs, suggests that the presence of EL in biomembranes impacts their curvature. The effect of ELs on the biophysics of membranes suggests that ELs have an important role in some physiological phenomena like the sporulation processes of some prokaryotic cells [32], the epithelium to mesenchymal transition of breast epithelium cell lines [33], the fusion of membranes with extracellular or intracellular vesicles [34] including neurotransmission vesicles [35]. ELs could also be used as a probe to evaluate climate change [36].

All the articles and reviews reporting the description of natural ELs [37], their quantification [38], their biological functions and their role in physio-pathological situations [39–40] invited the researchers to propose new analogues of ether lipids and to study their effects on biological systems. Most of the time the synthetic analogues of ELs aimed to interact with biomembranes via supramolecular interactions with the lipids and proteins that are embedded in these membranes. We can hypothesis that their mechanism of action occurs via a direct interaction with membrane proteins or by a modification of the biophysical properties of the membranes. It must be noted that the design of new bioactive ELs is an alternative to others strategies (e.g., small molecules, antibodies) to modulate membrane proteins functions. Recent results from our group [41–42] and others renew the interest to develop new ether lipids with promising perspectives to address the modulation of membrane proteins that represent a pertinent strategy for some diseases like cancers. Beside the use of synthetic ether lipids for the prevention or the treatment of cancers via different mechanisms, it must be noted that some synthetic ether lipids (e.g., Ino-C2-PAF) have also the ability to regulate genes involved in innate and acquired immune response [43–45] and genes related to inflammation with possible application for the treatment of inflammatory skin diseases [46].

The goal of this review is to report the synthetic approaches of ELs (natural or artificial) that could be helpful to the scientific community for different reasons: 1) to have an overview on the methods already available, 2) to invite scientists that are working on synthesis methodology to apply their works to the design of ELs analogues, 3) the methods reported in this review can be useful tools for the scientific community working on the analysis of EL that require the preparation of standards for analytical purposes. The analysis and quantification of ether lipids is a very important field of research and development that was previously reviewed [47–48]. In this review, we focus on synthesis methodologies applied to prepare alkyl ether lipids. The synthesis methods specifically designed for the preparation of alkenyl ether lipids (plasmalogen) are not reviewed herein. For each compound, their synthesis is reported jointly, when appropriate, with some elements relative to their biological activity. The next sections also include some examples of non-phosphorus glycosylated antitumor ether lipids (GAEL) but more details on the biology of these ether lipids can be found elsewhere [49–51]. The synthesis of analogues of archaeal ether lipids is not included herein but the reader can refer to other articles and reviews dedicated to archaeal lipids [52–53]. It is worth to be noted that some analogues of ether lipids are free of any glycerol unit or ether functions. These molecules that belong to the alkylphosphocholine (APC) class of compounds (e.g., miltefosine) are not illustrated in this review because they were previously reviewed [54–55].

## Review

### 1 Platelet Activating Factor (PAF) and PAF-analogues

The structure of PAF (1-*O*-alkyl-2-acetyl-*sn*-glyceryl-3-phosphorylcholine), which was reported in 1979 [56], features one ether function in *sn*-1 position, an acetyl group in *sn*-2 and a phosphocholine moiety as polar head group. In this section, we have included works that report the synthesis of PAF and, then, PAF-analogues. For the PAF-analogues, we have selected compounds that feature an acyl group in *sn*-2 position of the glycerol. Due to the number of PAF-analogues reported in the literature, we have done a selection, which is based on the methodology employed in order to have an overview of the most useful methods employed for the synthesis of PAF and PAF-analogues. The synthesis of alkyl EL involves the chemistry of glycerol or its direct precursors (e.g., glycidol, solketal, epichlorohydrin). The review of Lemaire et al., dedicated to the synthesis of glycerol ether, is complemental to this review article [57]. Of note, the review of Godfroid and Braquet attempted to decipher the binding site of PAF via a QSAR study [58].

#### 1.1 Synthesis of PAF and some building blocks

**Figure 2:**
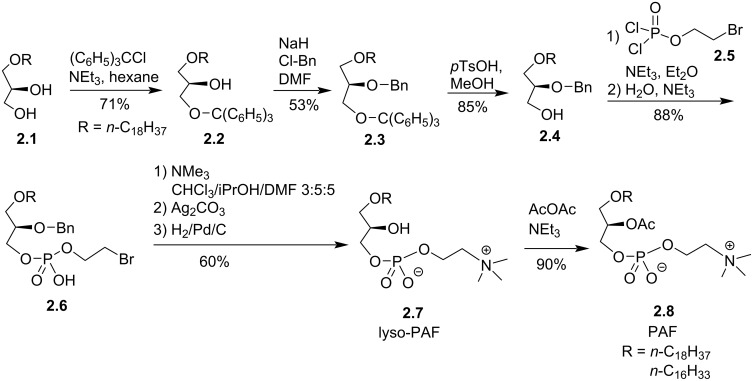
Synthesis of lyso-PAF and PAF from 1-*O*-alkylglycerol [64].

The platelet activating factor (PAF (**2.7**), 1-*O*-octadecyl-2-*O*-acetyl-*sn*-glycero-3-phosphocholine; Figure 2) is a natural compound involved in many biological processes [59], including, for instance, its capacity to aggregate platelets, to induce hypotensive effects [60] or to mediate anaphylaxis and inflammation processes [61]. A first hemi-synthesis was reported by Demopoulos et al. [56] and by Blank et al. [62] and two formal syntheses were reported by Benveniste et al. [63–64]. The second formal synthesis starts from the glycerol ether lipid **2.1** that reacted with trityl chloride to yield **2.2**. Then, the benzylation of the secondary alcohol produced **2.3**. The primary alcohol was deprotected in acidic conditions to produce **2.4**. The installation of the phosphocholine polar head group was achieved in two steps starting with the reaction of **2.4** with bromoethyl dichlorophosphate (**2.5**) to produce the phosphate derivative **2.6**. The treatment of **2.6** with trimethylamine produced an ammonium salt. A treatment with silver carbonate was applied to remove any traces of bromide salts. Then, the secondary alcohol was deprotected by hydrogenolysis to produce **2.7** (lyso-PAF). Finally, the acetylation of the secondary alcohol produced the final compound **2.8** (PAF) (Figure 2). A critical step in this synthesis scheme is the use of bromoethyl dichlorophosphate, prepared from POCl_3_ [65] and previously used for the synthesis of lecithin [66–67] and lecithin analogues [68], for the preparation of **2.6**.

**Figure 3:**
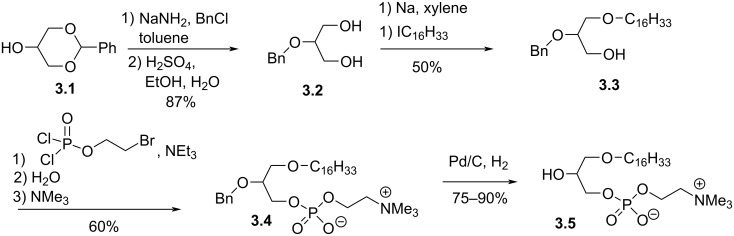
Synthesis of lyso-PAF from 1,3-benzylideneglycerol **3.1** [69].

It must be noted that a long time before the discovery of the structure of PAF, Arnold, Weltzien and Westphal reported the synthesis of lyso-PAF starting from 1,3-benzylideneglycerol (**3.1**) [69] (Figure 3). **3.2** was prepared from **3.1** following the methodology reported by West et al. [70]. Then, **3.2** was deprotonated with sodium and the alcoholate reacted with 1-iodohexadecane to produce **3.3** in 50% yield. The phosphocholine moiety was incorporated by using 2-bromoethyl dichlorophosphate as a key reagent and following a previously reported sequence [67]. The debenzylation by using hydrogenolysis conditions produced **3.5** in 75% to 90% yield.

**Figure 4:**
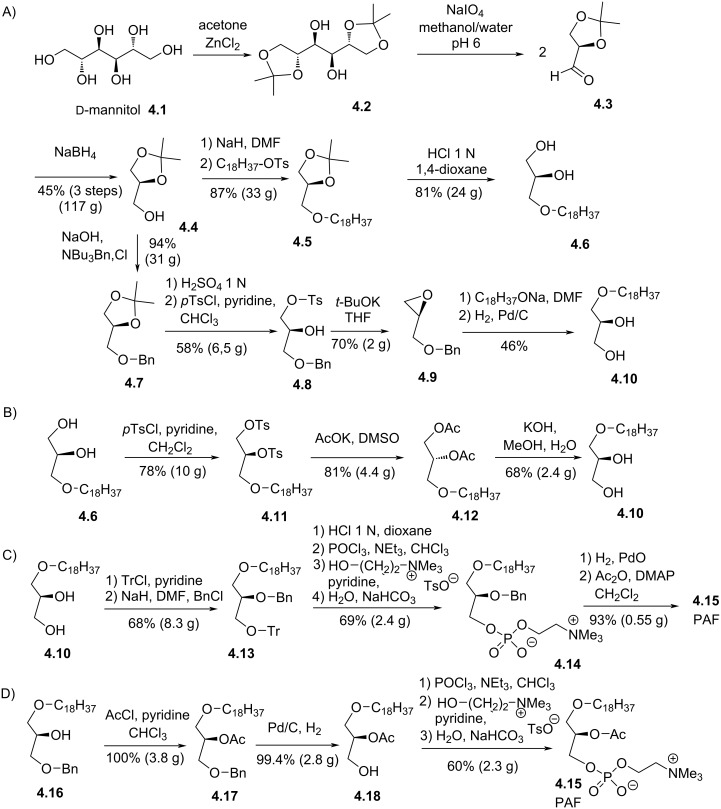
A) Synthesis of the two enantiomers of octadecylglycerol (**4.6** and **4.10**) from ᴅ-mannitol (**4.1**); B) sequence for the epimerization of **4.6** to **4.10**; C) installation of the phosphocholine moiety followed by acetylation; D) acetylation before the installation of the phosphocholine group [71].

In 1982, G. Hirth and R. Barner reported the synthesis of the two enantiomers of PAF [71]. The synthesis started from ᴅ-mannitol (**4.1**, Figure 4A). First, **4.1** was transformed to 1,2-isopropylidene-*sn*-glycerol (**4.4**) following a three-step sequence (45% yield over three steps) initially reported by H. Eilb [72]. Accordingly, ᴅ-mannitol was transformed in 1,2,5,6-diisopropylidene-ᴅ-mannitol (**4.2**) by reaction with acetone and ZnCl_2_. Of note, compound **4.2** was isolated with 5–10% of 1,2,3,4,5,6-triisopropylidene-ᴅ-mannitol. The oxidative cleavage of **4.2** with sodium periodate yields **4.3** that reacted immediately (one-pot procedure) with sodium borohydride to give **4.4**. It must be noted that this sequence does not induce racemization and that **4.4** can be stored for months with 0.5% of solid KOH acting as a stabilizer [72]. Then, **4.4** was alkylated with stearyl tosylate to produce **4.5**. The two alcohol functions of **4.5** were deprotected in acidic media to produce 3-*O*-octadecyl-*sn*-glycerol (**4.6**). The enantiomer of **4.6** was obtained from **4.4** by protecting the primary alcohol with a benzyl group to give **4.7**. Then, the deprotection of the two alcohol functions with H_2_SO_4_ in water followed by the tosylation of the primary alcohol produced **4.8**. The epoxidation of **4.8** occurred by reaction with *t*-BuOK in THF, thus producing **4.9** as a chiral electrophile. The regioselective opening of the epoxide is achieved by adding the octadecanol sodium salt. The intermediate was debenzylated by catalytic hydrogenolysis to produce 1-*O*-octadecyl-*sn*-glycerol (**4.10**). It must be noted that the authors, after the deprotection of the two alcohol functions of **4.7**, attempted the direct alkylation of the primary alcohol with octadecyltosylate. However, a mixture of mono and dialkylation was formed and were separated by chromatography. Because **4.6** was obtained in better yields and in only 3 steps, the epimerization of **4.6** to **4.10** was also reported (Figure 4B). This epimerization is achieved in three-step sequence that starts with the double tosylation of **4.6** to produce **4.11**. Then, the S_N_2 reaction with potassium acetate in DMSO produces the diester **4.12** with an inversion of the configuration of the chiral carbon atom. Then, **4.12** was hydrolyzed in the presence of KOH to produce **4.10**. The installation of the phosphocholine group was achieved following two schemes: a) Starting from the diol **4.10** (Figure 4C), tritylation and benzylation produced **4.13**. Then, the deprotection of the primary alcohol in acidic conditions allows introducing the phosphocholine polar head group by using POCl_3_ and the choline tosylate salt as reagents to yield **4.14**. Finally, the debenzylation of the secondary alcohol and its acylation produce PAF **4.15**. b) The second scheme is shorter (Figure 4D) and starts with the acylation of **4.16** to produce **4.17**. Then, the debenzylation of the primary alcohol produced **4.18**. Interestingly, the migration of the acetyl group from *sn*-2 to *sn*-3 position was not observed. Finally, the installation of the phosphocholine group was introduced following the same method to produce PAF **4.15**. By using the same methodology, the epimer of PAF **4.15** was synthesized.

**Figure 5:**
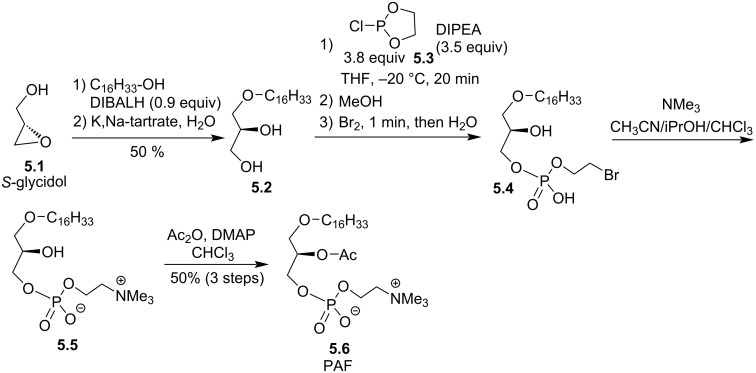
Four-step synthesis of PAF **5.6** from (*S*)-glycidol [73].

The group of Bittman reported in 1995 the synthesis of PAF with a four-step sequence starting from *S*-glycidol **5.1** in which the acylation of the *sn-*2 position was also achieved in the last step (Figure 5) [73]. DIBALH (diisobutylaluminium hydride) in toluene was added to hexadecanol in dichloromethane at 0 °C (Figure 5) to form in situ a lithium alcoholate. Then, *S*-glycidol was added at rt to produce in 50% yield the diol **5.2** after a regioselective opening of the epoxide. The lithium salts were removed by washing with potassium sodium tartrate (Seignette’s salt). Then, at low temperature an excess of 2-chloro-1,3,2-dioxaphospholane (**5.3**, 3.8 equiv) in the presence of diisopropylethylamine (DIPEA) reacted with the primary alcohol to produce, after an oxidation with Br_2_ and hydrolysis, the bromoethyl phosphate **5.4**. Finally, the quaternarization with trimethylamine produced **5.5** and the acetylation produced **5.6** PAF.

**Figure 6:**
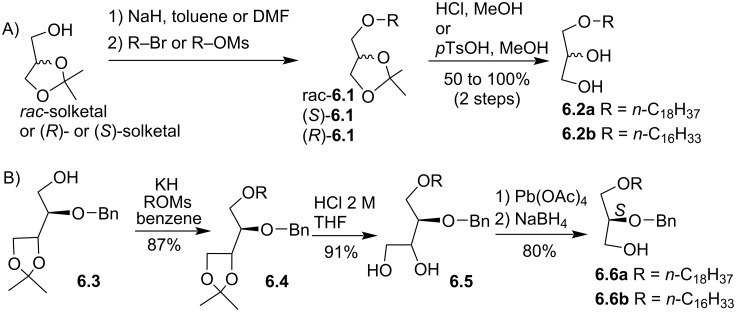
Synthesis of 1-*O*-alkylglycerol A) from solketal, B) from ᴅ- or ʟ-tartaric acid and the intermediate **6.3** [80].

The intermediate compounds like **6.2** (1-*O*-alkylglycerol) or the protected secondary alcohol **6.6**, either as enantiopure or racemic forms, are key intermediates for several syntheses of alkyl ELs or analogues. It is worth noticing that *rac*-1-*O*-octadecylglycerol (**6.2a**) is some time identified in the literature as batyl alcohol and *rac*-1-*O*-hexadecylglycerol (**6.2b**) as chimyl alcohol. For the racemic form (Figure 6A), the usual synthesis starts from racemic solketal which is deprotonated with potassium [74], NaH [75], NaNH_2_ [76] or KH and by using different solvents including benzene [74], toluene [76–77], THF [78], or DMF [75,79] and then alkylated with bromoalkyl [75–76] or mesylate lipid alcohol [74]. The same protocols (NaH, toluene or NaH, DMF) were applied to prepare enantiopure (*R*)-**6.1** or (*S*)-**6.1** from commercially available enantiopure solketal [75–76,78]. For the protected secondary alcohol of the glycerol derivative **6.6a** (Figure 6B), a stereoselective synthesis, starting from either ᴅ- or ʟ-tartaric acids, produced first the intermediate **6.3** (ᴅ- or ʟ-threitol) that was then alkylated with mesityl lipid alcohol to produce **6.4** [80–81]. The acetal protecting group was removed in acidic conditions and then the intermediate **6.5** was subjected to oxidative cleavage to yield an aldehyde that was reduced with NaBH_4_ to produce **6.6a**,**b** (Figure 6B).

**Figure 7:**
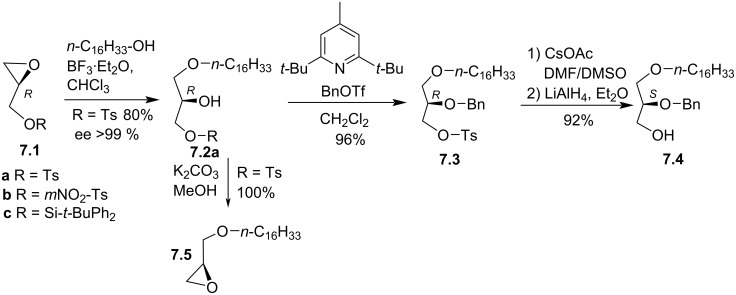
Synthesis of EL building blocks starting from substituted glycidol **7.1a**–**c** [82].

If solketal is frequently used as starting material for the synthesis of EL, it must be noted that glycidol is another substrate of interest available as racemic or enantiopure form. In 1988, Bittman and Guivisdalsky reported a new approach for the synthesis of stereocontrolled **7.4** starting from allyl alcohol (Figure 7) [82]. The Sharpless asymmetric epoxidation of allyl alcohol followed by tosylation produced glycidyl tosylate **7.1a** (Figure 7). The reaction of palmityl alcohol (C_16_H_33_-OH) in the presence of a catalytic amount of BF_3_ open regio- and stereoselectively the epoxide to produce **7.2a**. Interestingly, the same reaction can be achieved on the substituted glycidol **7.1b**,**c** with a yield of 70 to 80% and a regioselectivity that depends on the substituent (100% regioselectivity for R = Ts or *m*NO_2_-Ts and 90% of regioselectivity when R is *tert*-butyldiphenylsilane) [83–84]. Then, **7.2a** was converted to the epoxide **7.5** by cyclisation in the presence of potassium carbonate in methanol, thus producing the interesting building block **7.5**. A second option, optimized to avoid the formation of epoxide, used a hindered base and the reactive benzyltriflate as electrophile to achieve under mild conditions the benzylation of the alcohol function to yield **7.3**. Then, the removing of the tosyl group required a two-step sequence. First, **7.3** reacted with cesium acetate and then the resulting ester was reduced with LiAlH_4_ to produce **7.4** with control of its stereochemistry.

**Figure 8:**
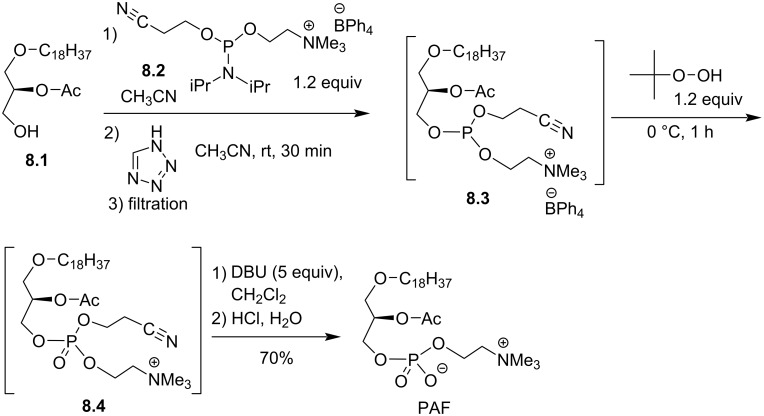
Synthesis of PAF **8.5** by using phosphoramidite **8.2** [86].

Very recently, a new method was developed for the incorporation of the phosphocholine polar head group that makes use of the phosphoramidite **8.2** which is a weakly air sensitive reagent [85]. This method was applied for the synthesis of **PAF** as illustrated in Figure 8 [86]. The alcohol **8.1** reacted with **8.2** in the presence of 1*H*-tetrazole to produce the trialkyl phosphite **8.3** that was oxidized with *tert*-butyl hydroperoxide to produce phosphate **8.4**. Then, β-elimination of the cyanoethyl protecting group produced **PAF** with a global yield of 70%. The limit of this method arises from the instability of the precursor **8.1** for which the acyl group can shift easily from the *sn-*2 to the *sn-*3 position. Noteworthy, the preparation of **8.2** was achieved in 3 steps from phosphorus trichloride and by using choline tetraphenylborate (prepared from choline chloride) [87] instead of the choline *p*-toluenesulfonate salt to improve the solubility of the salt.

#### 1.2 Analogues of PAF with modification on *sn-*1 position

**Figure 9:**
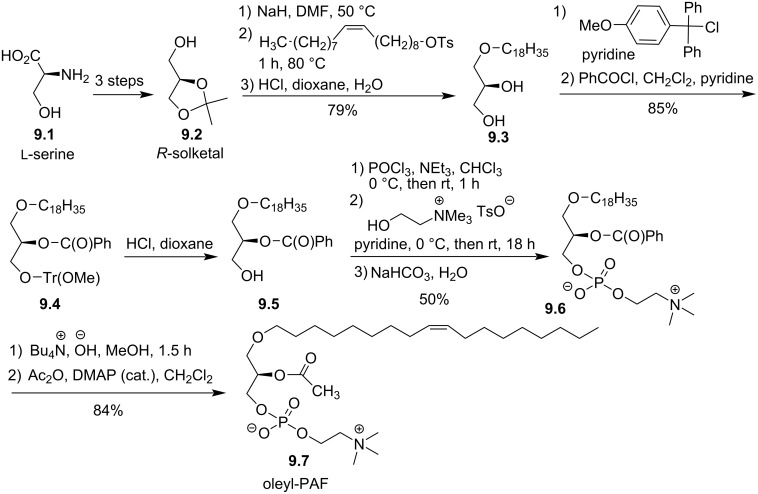
Synthesis of oleyl-PAF **9.7** from ʟ-serine [88].

PAF is characterized by a C16 or C18 saturated lipid chain at the *sn-*1 position. A first analogue, reported by Hirth et al. in 1983, consisted in replacing this saturated lipid chains by the mono-unsaturated oleyl ((*Z*)-octadec-9-enyl) lipid chain (Figure 9) [88]. The synthesis starts from serine **9.1** and produce (*R*)-solketal (**9.2**) following a three-step protocol [89] that was recently revisited [90] (diazotation, esterification, acetalization). Then, the incorporation of the oleyl chain (C_18_H_35_) was achieved by the deprotonation of solketal in DMF followed by the addition of oleyl alcohol tosylate. **9.3** was isolated after the hydrolysis in acidic conditions of the acetal protecting group. The protection of the primary alcohol required a protecting group that can be deprotected without affecting the C=C double bond of the oleyl chain. Accordingly, the primary alcohol was protected with *p*-methoxydiphenylmethyl (MeO-trityl) in pyridine and then, esterified in *sn-*2 position with benzoyl chloride to produce **9.4**. The deprotection of the primary alcohol under acidic conditions gave **9.5**. The polar head group was installed by using the PCl_3_ method. Then, the benzoyl protecting group was removed (89%) and the hydroxy group at the *sn-*2 position was esterified with acetic anhydride (94%) to produce oleyl-PAF **9.7**.

**Figure 10:**
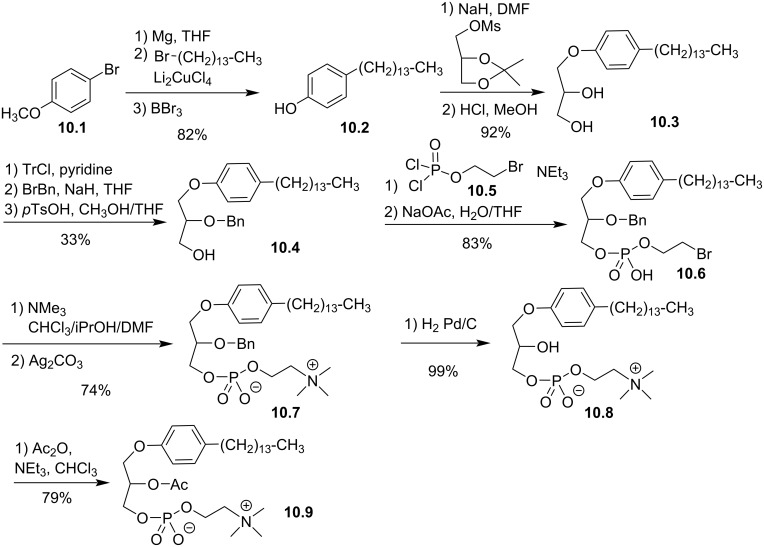
Synthesis of racemic analogues of lyso-PAF **10.8** and PAF **10.9** featuring a phenyl group between the glycerol and the lipid chain (racemic synthesis) [91].

Different modifications involved the *sn-*1 position of the glycerol moiety. In 1984, Wissner et al., reported the incorporation of a phenyl group between the glycerol and the lipid chain. The lipid chain was bonded to the aromatic ring in either *ortho-*, *meta-,* and *para*-position [91]. The incorporation of a phenyl moiety starts with the reaction of the Grignard reagent formed from 4-bromoanisole (**10.1**, the other isomers 2- or 3-bromoanisole were also reported) with bromotetradecane in the presence of a copper salt (Figure 10). Then, the deprotection of the phenol function with BBr_3_ produced **10.2**. The deprotonation of the phenol function with NaH in DMF and its reaction with solketal mesylate produced, after the deprotection of diol with HCl, the aryl ether glycerol **10.3**. The protection of the *sn-*2 position with a benzyl group was achieved by a classical tritylation of the primary alcohol, benzylation of the secondary alcohol and removing the trityl protecting group. The low yield of this three-step sequence is due to the incorporation of the trityl group, which is reported with 42% yield. The next steps correspond to the adaptation of previously reported methods with the use of bromoethyl dichlorophosphate (**10.5**) as precursor of the phosphocholine polar head group. The last steps (debenzylation and acetylation) produced successively the lyso derivative **10.8** and the acetylated compound **10.9**.

**Figure 11:**
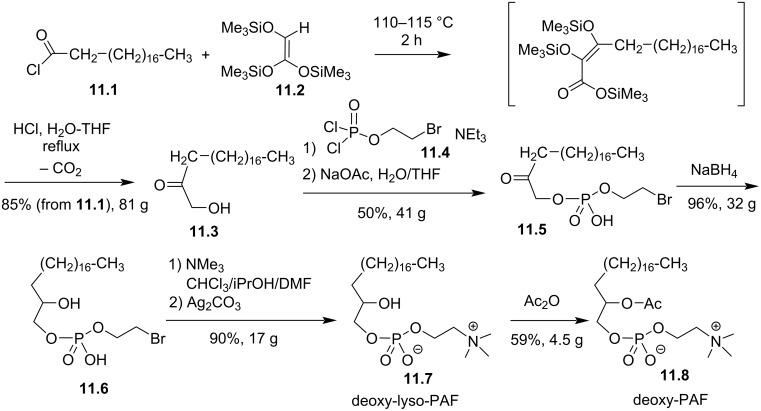
Synthesis of racemic deoxy-lyso-PAF **11.7** and deoxy-PAF **11.8** [91].

The suppression of the ether function in position *sn*-1 of PAF or its replacement by a thioether function were also reported in 1984 with the aim to identify new compounds that could modulate platelet aggregation or featuring hypotensive effects. First, Wissner et al. reported the synthesis of racemic *sn-*1-deoxy-PAF **11.8** (Figure 11) [91]. First, *n*-octadecanoic acid chloride (**11.1**) reacted with tris[(trimethylsilyl)oxy]ethylene (**11.2**) [92] to produce, after acidic hydrolysis and subsequent decarboxylation, compound **11.3**. Then, the phosphate moiety was introduced via the use of bromoethyl dichlorophosphate **11.4** to produce **11.5**. The reduction of the ketone with NaBH_4_ produced **11.6**, and then the incorporation of the trimethylammonium group produced deoxy-lyso-PAF **11.7**. Finally, the acetylation of the secondary alcohol produced racemic deoxy-PAF **11.8**. The biological evaluations of deoxy-PAF **11.8** have shown that it was less efficient than PAF to reduce blood pressure and to stimulate platelet aggregation [91].

**Figure 12:**
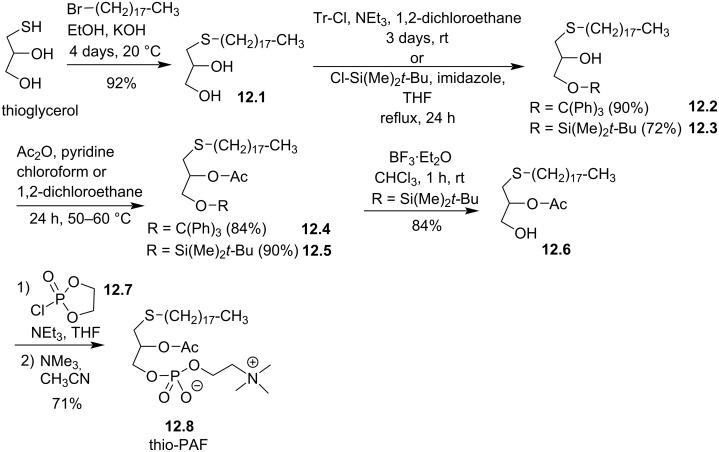
Synthesis of racemic thio-PAF **12.8** [93].

The synthesis of the racemic *sn-*1-thio-PAF **12.8** was reported by Maffrand et al. in 1984 (Figure 12) [93]. The sequence starts with the alkylation of the racemic thioglycerol with bromooctadecane in the presence of potassium hydroxide. Then, the protection of the primary alcohol was achieved either with trityl chloride or with dimethyl-*tert*-butylchlorosilane. The authors also attempted to use chlorotrimethylsilane but the protection was not regioselective (a mixture of primary and secondary protected alcohols was formed). The acylation of the secondary alcohol was then achieved with acetic anhydride in the presence of pyridine. Then, the deprotection of the trityl moiety of compound **12.4** by catalytic hydrogenation failed whereas heating it in 75% acetic acid solution produced the deprotected compound but migration of the acyl group from the *sn-*2 to the *sn-*3 position lead to an inseparable mixture of regioisomers. A selective desilylation of **12.5** was finally achieved with BF_3_·Et_2_O producing **12.6** without migration of the acyl group. Then, the phosphocholine polar head group was introduced with a sequential one-pot procedure using first 2-chloro-2-oxo-1,3,2-dioxaphospholane (**12.7**) to produce a cyclic phosphate as intermediate that subsequently reacted with trimethylamine to produce thio-PAF **12.8**. The opening of dioxaphospholane with trimethylamine was initially reported by the group of P. Chabrier [94] and subsequently applied for the synthesis of diacyl-glycerophospholipids [95] and for the synthesis of ether lipids by J. Hajdu et al. [96].

**Figure 13:**
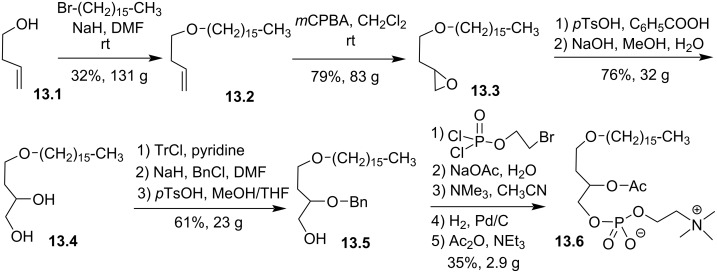
Racemic synthesis of **13.6** to illustrate the modification of the glycerol backbone by adding a methylene unit [97].

Wissner et al. modified the glycerol backbone by adding methylene units between either *sn-*1 and *sn-*2 positions or *sn-*2 and *sn-*3 positions [97]. The incorporation of one methylene unit is shown in Figure 13 as an illustration of all these possibilities. But-3-en-1-ol (**13.1**) was alkylated with bromohexadecane to produce the ether **13.2**. The epoxidation of the carbon–carbon double bond with *m*CPBA produced the epoxide **13.3**. Then, the addition of benzoic acid in the presence of acid catalysis produced an ester that was saponified to yield the diol **13.4**. A three-step sequence is applied to produce compound **13.5** that features a secondary alcohol protected with a benzyl group. Then, the installation of the phosphocholine moiety (67%) followed by the deprotection of the secondary alcohol (100%) and its acetylation (53%) produced **13.6**.

**Figure 14:**
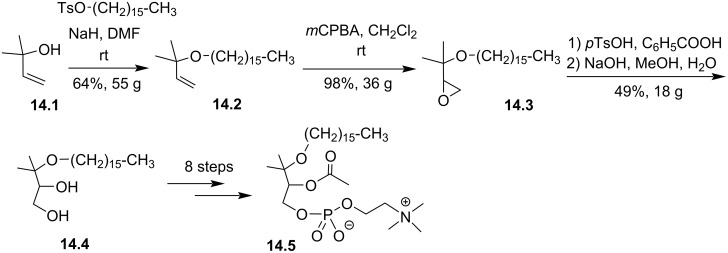
Racemic synthesis of **14.5** as an illustration of the introduction of methyl substituents on the glycerol backbone of PAF [97].

Wissner et al. also reported the incorporation of a *gem*-dimethyl substituent on the glycerol backbone [97]. One illustration of this structural modification is shown in Figure 14. 2-Methylbut-3-en-2-ol (**14.1**) was used as substrate and the next steps were almost comparable to those reported in Figure 13. The key intermediate **14.4** was isolated after a three-step sequence and used to prepare **14.5**.

#### 1.3 Analogues of PAF with modification at the *sn*-2 position

**Figure 15:**
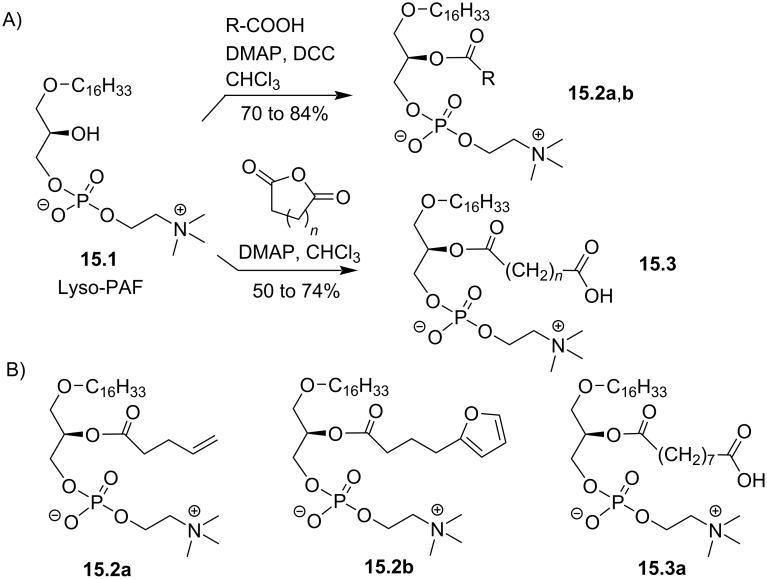
Synthesis of functionalized *sn-*2-acyl chains of PC-EL; A) Steglich esterification or acylation reaction with cyclic acid anhydride; B) examples of reported compounds [98–99].

The acylation of lyso-PAF with a series of functionalized carboxylic acid was reported in a series of articles from the group of Salomon [98–99]. This group aimed to identify natural compounds that could be formed by the oxidation of ether lipids featuring a polyunsaturated acyl chain in *sn-*2 position. This work includes, in addition to the oxidation of such type of polyunsaturated EL incorporated in liposomes [99], the formal synthesis of some oxidized derivatives. As an illustration, lyso-PAF **15.1** (extracted from egg albumin) was acylated using Steglich conditions with ω-unsaturated carboxylic acid to produce **15.2a** [99] (Figure 15A and B) or with functionalized furane to produce **15.2b**. The acylation was also achieved by reaction with cyclic acid anhydride to place in ω-position a carboxylic acid function as exemplified with **15.3a** [98].

**Figure 16:**
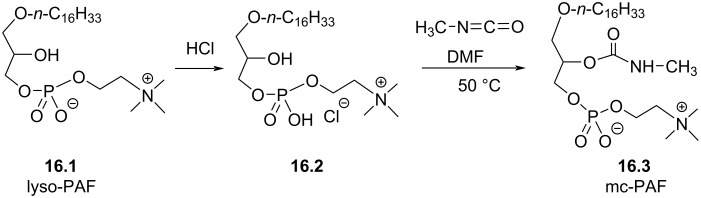
Synthesis of racemic mc-PAF (**16.3**), a carbamate analogue of PAF [102].

The acetyl group present in the structure of PAF in position *sn-*2 of glycerol is readily hydrolyzed in vivo and in serum [100–101]. With the aim to produce more stable compounds, the modification of the *sn-*2 position of the glycerol was reported. A first option consisted in placing a carbamate function leading to the synthesis of methyl carbamoyl-PAF (1-*O*-hexadecyl-2-*O*-(*N*-methylcarbamoyl)-*sn*-glycero-3-phosphocholine, mc-PAF (**16.3**)). Its synthesis starts with the protonation of lyso-PAF to form **16.2** that subsequently reacted with methylisocyanate to produce mc-PAF **16.3** (Figure 16) [102]. The yield of this reaction was not reported. mc-PAF **16.3**, which is indeed much more stable than PAF in serum, increases the formation of prostaglandin E2 from astrocyte cortical cell culture [103] and affect memory [104].

**Figure 17:**
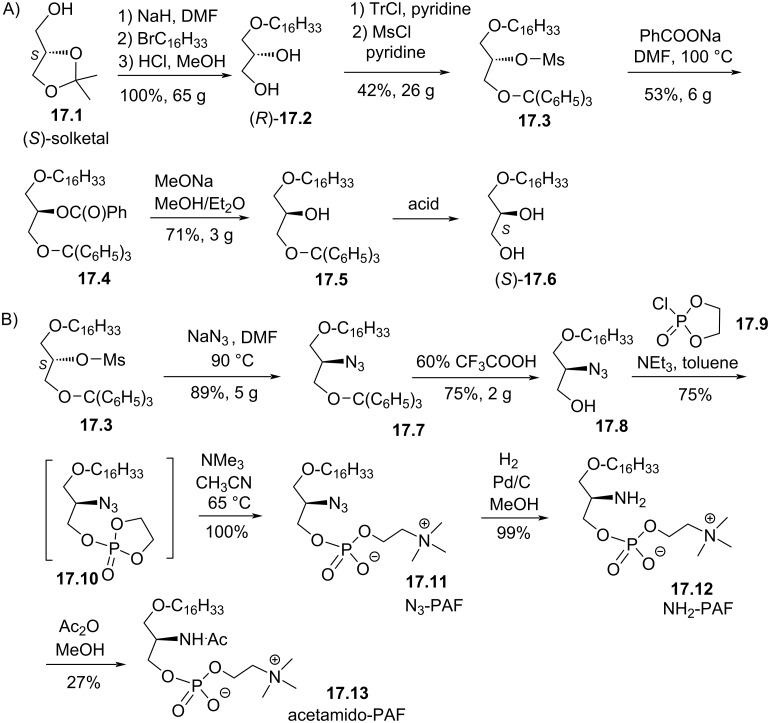
A) Synthesis of (*R*)-**17.2** and (*S*)-**17.6** starting from (S)-solketal (**17.1**); B) synthesis of N_3_-PAF (**17.11**), NH_2_-PAF (**17.12**) and acetamido-PAF (**17.13**) [79].

Ponpipom et al. have reported the synthesis of PAF-analogues featuring in *sn*-2 position either an azide, amine or acetamide group [79]. In each case, both enantiomers were reported. For the control of the chirality in position 2 of glycerol, (*S*)-solketal (**17.1**) was used as starting material to prepare first the hexadecylglycerol (*R*)-**17.2** which was converted to its enantiomer following a five-step sequence (Figure 17A). First, tritylation and mesylation produced **17.3**. Then, the nucleophilic substitution (S_N_2) reaction of benzoate with **17.3** produced the benzoate ester **17.4** with an inversion of configuration. Then, the two protecting groups (ester and trityl) were removed to produce (*S*)-**17.6**. The modification of the *sn*-2 position is illustrated in Figure 17B starting from the mesylate derivative (*S*)-**17.3**. Its reaction with sodium azide produced, following a S_N_2 reaction, the azido derivative **17.7**. The deprotection of the alcohol function produced **17.8** that subsequently reacted with 2-chloro-2-oxo-1,3,2-dioxaphospholane (**17.9**) in the presence of trimethylamine to yield the phosphate **17.10** as an intermediate. Then, its reaction with trimethylamine produced the phosphocholine moiety and compound N_3_-PAF (**17.11**). Then, the amine (NH_2_-PAF) **17.12** was formed by catalytic hydrogenation and subsequently the (acetamido-PAF) **17.13** was formed by acetylation of the amine with acetic anhydride. It is worth noticing that acetamido-PAF **17.13** was previously reported following a different synthesis scheme starting from serine as chiral precursor [96,105]. Recently, is was reported that the acetamido-PAF **17.13** is an activator of the TRPV2 channel leading to constitutive Ca^2+^ entry thus influencing breast cancer cell migration [106].

#### 1.4 Analogues of PAF with modification at the *sn*-3 position

**Figure 18:**
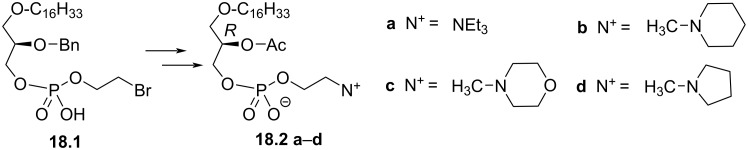
Modification of the phosphocholine polar head to produce PAF analogues [81].

Finally, a series of PAF’s analogues were reported by changing the structure of the phosphocholine polar head group. Ohno et al. replaced the trimethylammonium moiety by either triethylammonium **18.2a**, *N*-methylpiperidinium **18.2b**, *N*-methylmorpholinium **18.2c**, or *N*-methylpyrrolidinium **18.2d** (Figure 18). **18.1** was used as substrate and was transformed in compounds **18.2a**–**d** following a three-step sequence (introduction of the ammonium, debenzylation and acetylation) [81]. Two of these PAF-analogues (**18.2b** and **18.2d**) were more efficient than PAF for platelet aggregation and for their ability to reduce hypertension.

**Figure 19:**
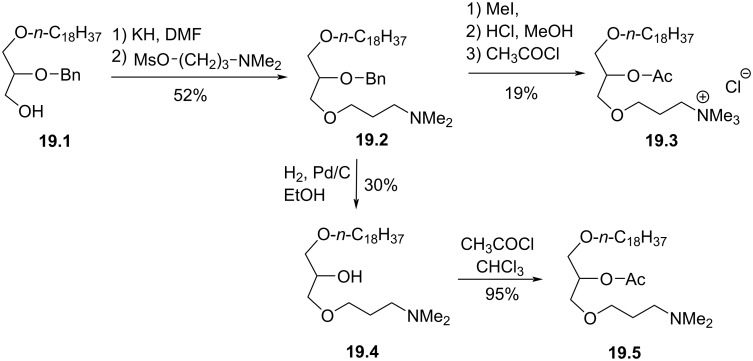
Racemic PAF analogues **19.3** and **19.5** characterized by the absence of the phosphate group [107].

Heymans et al. reported in 1985 a series of PAF-analogues featuring a polar head group in which the phosphate function was replaced by an ether function (Figure 19) [107]. The synthesis starts from the lipid alcohol **19.1** which was deprotonated and alkylated with a mesylamine to produce **19.2**. Then, the formation of the ammonium by reaction with methyl iodide followed by the deprotection of the benzyl protecting group under acidic conditions and the acetylation produced the PAF-analogue **19.3**. The analogue **19.5** was prepared from **19.2** by debenzylation using catalytic hydrogenation to produce **19.4** that was then acetylated to produce **19.5**. **19.3** or **19.5** were not able to induce either platelet aggregation or bronco-constrictive activities.

**Figure 20:**
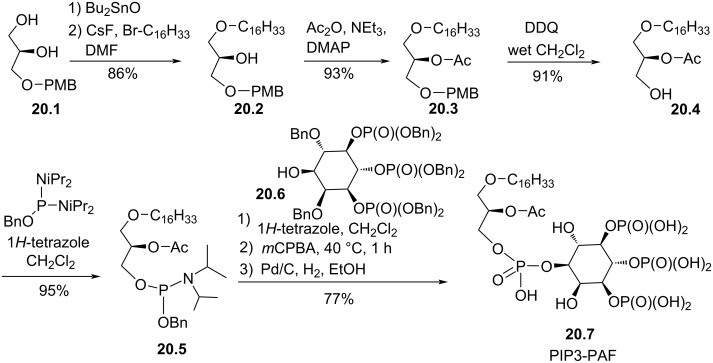
Synthesis of PIP3-PAF (**20.7**) [108].

A third modification of the phosphocholine polar head group consisted in replacing the ammonium moiety by *myo*-inositole-3,4,5-trisphosphate to prepare PIP3-PAF (Figure 20) [108]. The synthesis reported by Wang et al. in 2001 started from the enantiopure diol **20.1**, protected with a *para*-methoxybenzyl (PMB) group at the *sn*-3 position. **20.1** was selectively alkylated on the primary alcohol to produce **20.2** via the use of dibutyltin oxide as selective reagent for the alkylation of diols [109]. For this reaction, CsF was added to increase the reactivity of the alkyl bromide, likely by a combined effect that includes the interaction of the cesium cation with the halogen atom and the activation of the Sn–O bond of the stannylene acetal via a pentacoordinated intermediate with the fluoride anion [110]. The acetylation of the secondary alcohol and the deprotection of the primary alcohol with 2,3-dichloro-5,6-dicyano-1,4-benzoquinone (DDQ) produced **20.4**. Then, the incorporation of the phosphoinositol moiety was achieved by using phosphoramidite chemistry. First, the alcohol **20.4** reacted with *O*-benzyl-*N*,*N*,*N’*,*N’*-tetraisopropylphosphorodiamidite to produce the phosphoramidite **20.5** that subsequently reacted with dibenzyl-tris(dibenzylphosphate) *myo*-inositol (**20.6**). The oxidation of the phosphite intermediate with *m*-CPBA followed by the catalytic hydrogenolysis of the benzyl protecting groups produced PIP3-PAF (**20.7**).

### 2 Edelfosine and diether analogues

PAF and PAF-analogues that feature an acyl or more generally an ester group in *sn-*2 position are unstable in physiological environment. The introduction of a second ether function in *sn-*2 position was applied to increase the stability of such type of compounds in biological media. The initial investigations evaluated the capacity of PAF analogues, featuring higher physiological stability, to aggregate platelets [111]. All these characteristics led to the development of edelfosine and other EL analogues. It is worth noting, that the review articles of Houlihan et al. [112], Brachwitz and Vollgraf [113] and Principe and Braquet [114] reporting the biological effects of phospholipid antitumor agents but without discussing the synthesis approaches, are complementary to the synthesis aspects presented in the upcoming section.

#### 2.1 Synthesis of edelfosine

**Figure 21:**
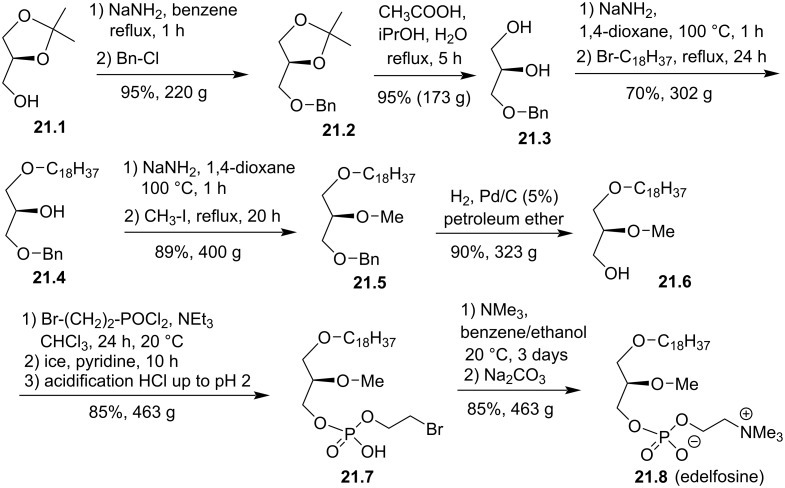
Large-scale synthesis of C18-edelfosine (**21.8**) [116].

Edelfosine (1-*O*-octadecyl-2-*O*-methylglycero-3-phosphocholine) ET-18-OCH_3_ or ET-16-OCH_3_ is an alkyl ether lipid with a methoxy group in *sn*-2 position and a *sn*-3 phosphocholine moiety. The length of the saturated lipid chain is composed by either 16 or 18 carbon atoms in the backbone and the stereochemistry of the carbon atom in *sn*-2 position can be racemic or stereocontrolled. Edelfosine was first synthesized by G. Kny in 1969 [115] but to the best of our knowledge, R. Berchtold reported in 1982 the first synthesis in large quantities with a control of the chirality at the *sn*-2 position (Figure 21) [116]. The synthesis starts from (S)-1,2-isopropylideneglycerol (**21.1**). The deprotonation of the primary alcohol with sodium amide followed by the benzylation of the sodium alcoholate produced **21.2** in 95% yield. The deprotection of the acetal was achieved with acetic acid in a mixture of isopropanol and water to give **21.3**. Then, a critical step is the alkylation of the primary alcohol in the presence of the unprotected secondary alcohol. This step was achieved by the deprotonation of **21.3** followed by the reaction with 1-bromooctadecane. Compound **21.4** was purified by chromatography on silica gel followed by a recrystallization in acetone. Deprotonation of the secondary alcohol present in **21.4** followed by the addition of iodomethane as electrophile produced **21.5** in 89% yield. The debenzylation of the *sn*-3 alcohol was achieved by catalytic hydrogenolysis to give **21.6**. Then, the phosphocholine moiety was introduced by using 2-bromoethyl phosphorodichloridate as key reagent to give **21.7** that subsequently reacted with trimethylamine to produce **21.8** (edelfosine; Et-18-OCH_3_).

**Figure 22:**
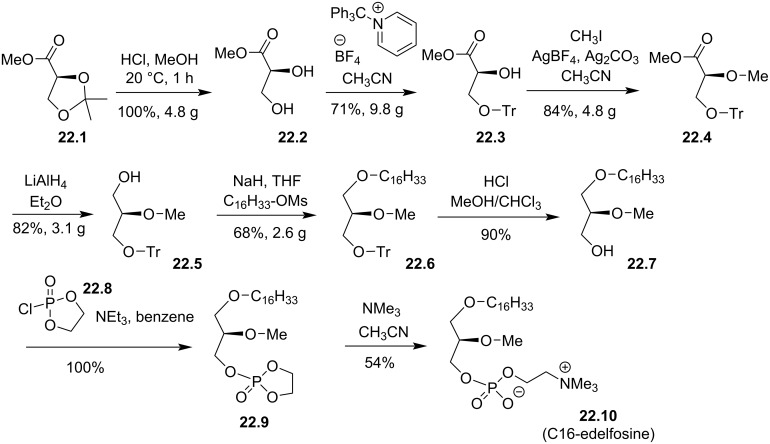
Synthesis of C16-edelfosine (**22.10**) starting from isopropylidene-ʟ-glyceric acid methyl ester (**22.1**) as a chiral substrate [118].

In 1987 [117] and 1988 [118], J. Hadju and S. K. Bhatia reported a stereocontrolled synthesis of edelfosine starting from the commercial isopropylidene-ʟ-glyceric acid methyl ester (**22.1**, Figure 22). The synthesis starts with a trans-acetalization step to remove the acetal protecting group thus producing **22.2**. Then, the primary alcohol was protected by reaction with tritylpyridinium tetrafluoroborate salt to produce **22.3**. In the next step, the secondary alcohol was methylated with iodomethane in the presence of silver salts (AgBF_4_) and silver base (Ag_2_CO_3_) to give **22.4**. Alcohol **22.5** was isolated after the reduction of the ester group of **22.4**. Then, the C16 alkyl chain was introduced to form **22.6** by the reaction of the alcoholate formed by deprotonation of **22.5** and hexadecyl mesylate. Then, the trityl group was removed under acidic conditions in a mixture of methanol and chloroform to give **22.7**. The last two steps consist in introducing the phosphocholine moiety. A first step consists in the reaction of 2-chloro-2-oxo-1,2,3-dioxaphospholane (**22.8**) with **22.7** to yield **22.9**. Then, the heterocycle was opened by reaction with trimethylamine to produce **22.10**. However, the last step features the lower yield (54%) of this 8-step synthesis.

**Figure 23:**
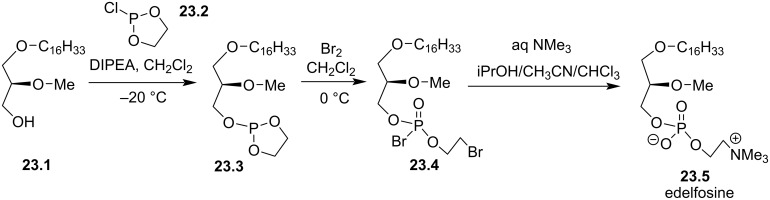
Phosphocholine moiety installation by the use of chlorophosphite **23.2** as key reagent [119].

In 1994, Bittman et al. reported an alternative strategy to introduce the phosphocholine moiety by the preparation of a cyclic phosphite as a key intermediate [119]. This one-pot three-step sequence starts with the reaction of **23.1** with chlorophosphite **23.2** in the presence of diisopropylethylamine (DIPEA, Figure 23). Then, the intermediate **23.3** reacts with Br_2_ to produce the intermediate **23.4**. Finally, the addition of trimethylamine in an aqueous-organic medium produces edelfosine (**23.5**).

**Figure 24:**
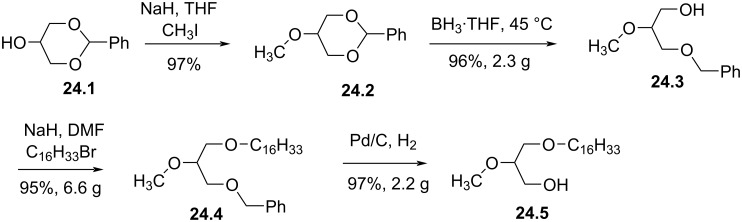
Synthesis of *rac*-1-alkyl-2-*O*-methylglycerol (AMG) [120].

For most of the syntheses of edelfosine reported above, the phosphocholine polar head group was introduced during the last steps. Accordingly, the glycerol moiety functionalized with a lipid chain (C16 or C18) and a methoxy group, respectively, in *sn*-1 and *sn*-2 position of glycerol (alkyl-methoxy-glycerol – AMG) constitutes an important building unit. The synthesis of this intermediate was prepared as a racemic (Figure 24) or stereocontrolled form. One strategy to prepare *rac*-AMG starts from **24.1** (**24.1** can be prepared from glycerol) [120–121]. Methylation of **24.1** is readily achieved by the deprotonation of the secondary alcohol with NaH followed by the methylation with iodomethane. Then, the cleavage of the acetal occurs by reaction with BH_3_·THF to give **24.3**. Then, the primary alcohol was alkylated with the lipid chain (e.g., C_16_H_33_) to produce **24.4**. Finally, the benzyl protecting group was removed by catalytic hydrogenolysis to produce **24.5**.

**Figure 25:**
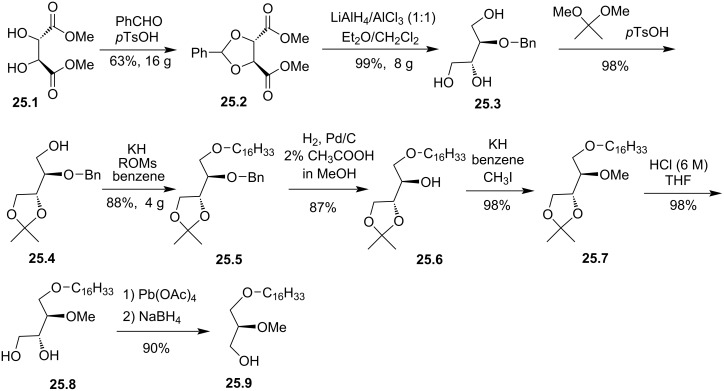
Synthesis of stereocontrolled 1-alkyl-2-*O*-methyl glycerol **25.9** (AMG) from dimethyl ᴅ-tartrate [81].

In addition to the stereocontrolled synthesis of AMG reported in Figure 21 [116] and Figure 22 [118], another possibility uses dimethyl-ᴅ-tartrate (**25.1**) as chiral precursor (Figure 25) [81]. This 8-step synthesis starts with the protection of the diol to form the benzylidene tartrate **25.2**. Then, a reductive cleavage of the acetal and the reduction of the two ester functions produced 2-*O*-benzyl-ᴅ-threitol (**25.3**) in nearly quantitative yield. The acetalization of the *gem*-diol produce **25.4** that was deprotonated with KH and alkylated with hexadecyl mesylate to produce **25.5**. The deprotection of the secondary alcohol under catalytic hydrogenolysis conditions produced **25.6**. Then, the deprotonation of **25.6** followed by the alkylation of the alcoholate with iodomethane produced **25.7**. The oxidative cleavage of the *gem*-diol with lead acetate produced an aldehyde that was reduced with sodium borohydride to give the alcohol **25.9**.

#### 2.2 Analogues of edelfosine

In view of the remarkable effects of edelfosine on cancer cells [122], its action as a proapoptotic agent [123–124] and its effect on lipid raft [125–126] or its action against leishmania [127–128], the synthesis of analogues of edelfosine are numerous and aimed to improve its efficacy and/or the selectivity that could also reduce side effects. In the next section, we report a selection of the molecular modifications that are classified depending on the position of the glycerol moiety (*sn*-1 lipid chain; *sn*-2; *sn*-3 polar head group) where the molecular structure is altered. However, the incorporation of a saccharide unit or an inositol moiety is included in subsequent sections.

**Figure 26:**
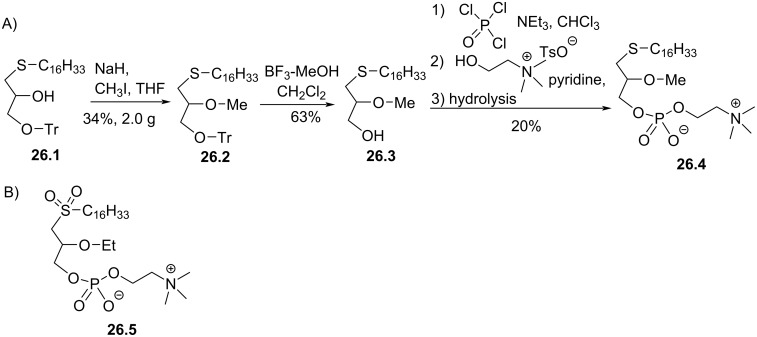
A) Racemic synthesis of thioether **26.4** [129–130], B) structure of sulfone analogue **26.5** [129].

**Modulation *sn*-1:** In 1986, Morris-Natschke et al. [129–130] reported a racemic synthesis of thioether analogues of edelfosine using thioglycerol as precursor. The reaction started with the S-alkylation of thioglycerol by bromo- or iodoalkyl chains as previously reported [131]. Then, the primary alcohol was protected with a trityl group to form **26.1** (Figure 26A). The secondary alcohol was first deprotonated with sodium hydride and alkylated with iodomethane to give **26.2**. **26.3** was isolated after the deprotection of the primary alcohol with BF_3_. Then, the installation of the phosphocholine polar head used POCl_3_ and choline tosylate to produce in low yield the phosphocholine thioether lipid **26.4**. In this study, the authors reported that compound **26.4** has similar cell toxicity than edelfosine-C18 on HL-60 leukemic cells [129–130]. The authors also reported that the sulfone **26.5** (Figure 26B) had similar cytotoxicity than edelfosine-C18. In 1987, the group of E. J. Modest reported that the thioether **26.4** featured comparable cytotoxicity than C18-edelfosine on two leukemic cell lines (HL60 and K562) [132]. Finally, it must be noted that choline tetraphenylborate salt was advantageously used for the preparation of glycerophospholipids by Harbison and Griffin [133]. This salt of choline is poorly hygroscopic and is more soluble in organic media like pyridine.

**Figure 27:**
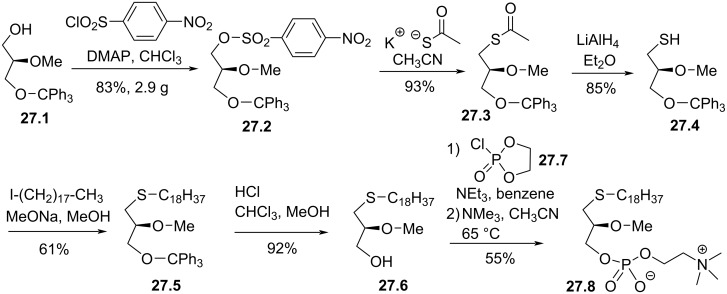
Stereocontrolled synthesis of C18-edelfosine thioether analogue **27.8** [118].

A stereocontrolled synthesis of **26.4** was reported by Hajdu and Bhatia in 1988. The sequence starts from **27.1** that was prepared from ʟ-glyceric acid (Figure 27) [118]. Then, the free alcohol was converted as an efficient leaving group by reaction with 4-nitrobenzenesulfonyl chloride in the presence of dimethylaminopyridine (DMAP). Then, **27.2** reacted with potassium thioacetate to produce the thioester **27.3**. Its reduction with lithium aluminium hydride produced the free thiol **27.4** that was used as nucleophile on octadecyl iodide to install the C18 lipid chain. The deprotection of the primary alcohol produced **27.6** that, in a two-step sequence, was used to install the phosphocholine moiety to produce **27.8**.

**Figure 28:**
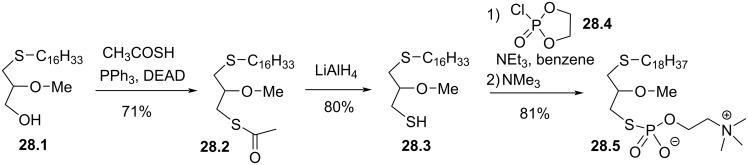
Synthesis of thioether **28.4** that include a thiophosphate function [134].

The double structural modification of edelfosine that consists to link the lipid chain via a thioether function and to replace the phosphate moiety by a thiophosphate was reported by Markowska et al. in 1993 [134]. As detailed in Figure 28, the synthesis starts with a Mitsunobu esterification of **28.1** with thioacetic acid to produce the thioester **28.2**. Then, the reduction with lithium aluminium hydride produced the thiol **28.3**. Finally, the phosphocholine moiety was introduced by using **28.4** followed by the opening of the intermediate heterocycle with trimethylamine to produce **28.5**.

**Figure 29:**
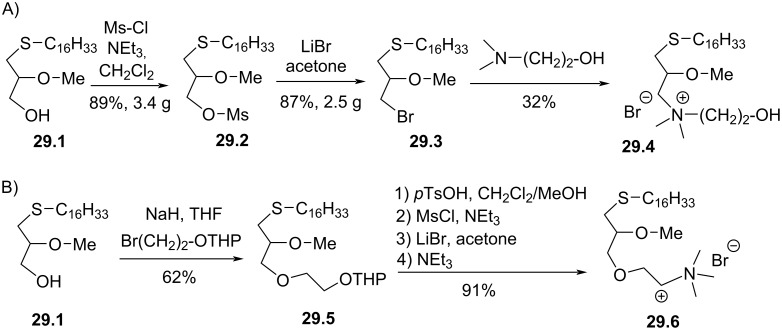
Synthesis of ammonium thioether **29.4** and **29.6** [135].

It must be noted that C. Piantadosi, S. Morris-Natschke et al. reported in 1990 [135] a series of alkyl thioethers with additional modifications in position *sn*-3. First, the thioether **29.1** (Figure 29A) was mesylated to **29.2**. Then, a bromine atom was introduced via a nucleophilic substitution with LiBr in acetone to form **29.3**. Finally, the primary bromide was used to introduce different ammonium salts as illustrated with compound **29.4**. A variation of this sequence consisted in placing an ether function in position *sn*-3 (Figure 29B). Accordingly, a Williamson ether synthesis was applied to **29.1** with bromoethanol protected with a THP group to produce **29.5**. Then, the deprotection of the THP group, the installation of a mesyl group, the exchange with LiBr and the final reaction with trimethylamine produced **29.6**.

**Figure 30:**
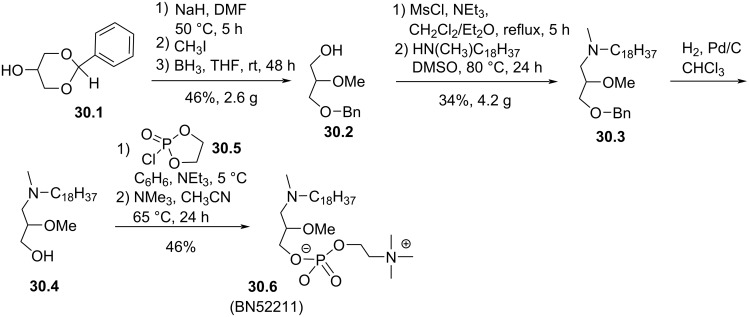
Synthesis of the *N*-methylamino analogue of edelfosine **30.6** (BN52211) [138].

The replacement of the oxygen atom located in *sn*-1 position by a methylamino group corresponds to a compound known as BN52211 (**30.6**, Figure 30). This compound was used in many studies for its antitumor cytotoxicity [136] or its immunologic properties [137]. To the best of our knowledge, the synthesis of this compound is only reported in a patent published in 1991 by P. Braquet et al. [138]. The reaction starts with the deprotonation of **30.1** to produce the alcoholate that was alkylated with iodomethane. Then, the reductive opening reaction of the 1,3-dioxane heterocycle in the presence of BH_3_ as reducing agent produced **30.2**. The mesylation of **30.2** followed by the nucleophilic addition of *N*-methyloctacedylamine produced **30.3**. The debenzylation of **30.3** to produce **30.4** followed by the installation of the phosphocholine moiety by using the chlorophosphate **30.5** yield **30.6** (BN52211).

**Figure 31:**
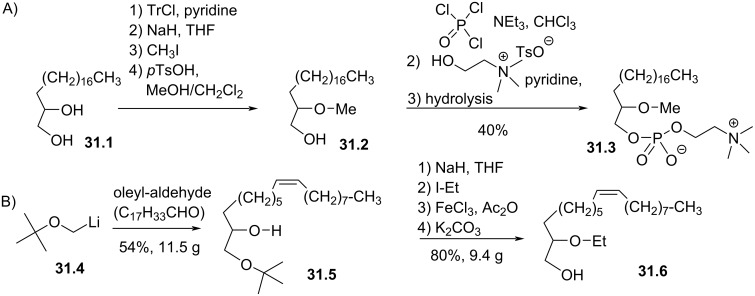
Synthesis of 1-desoxy analogues of edelfosine; A) with a saturated alkyl chain; B) synthesis of the precursor **31.6** with a mono-unsaturated lipid chain [139].

The replacement of the oxygen atom at the *sn*-1 position of the glycerol by a methylene unit (*sn*-1-desoxy glycerol derivatives) was reported by Bonjouklian et al. in 1986 [139]. These authors reported the synthesis of edelfosine analogues with saturated, unsaturated or polyunsaturated lipid chains. For the analogues with a saturated lipid chain (Figure 31A), the reaction started from the diol **31.1**. A four-step sequence, which is not detailed in their publication, produced the intermediate **31.2**. Then, the phosphocholine moiety was introduced by using POCl_3_ and choline tosylate to produce **31.3**. For the unsaturated and polyunsaturated analogues, the reaction started with the metalation of *tert*-butyl methyl ether according to the conditions reported by Corey and Eckrich [140], followed by the nucleophilic addition on unsaturated or polyunsaturated aldehyde to produce, as an example, **31.5**. Then, the alkylation of the secondary alcohol with iodoethane and the transformation of the *t*-Bu ether in acetyl ester following the method of Ganem and Small [141], produced, after saponification, the key intermediate **31.6**.

**Figure 32:**
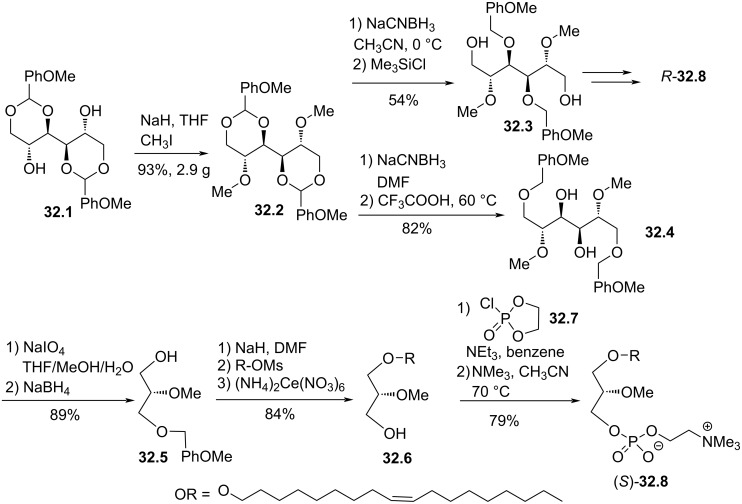
Stereocontrolled synthesis of edelfosine analogue (*S*)-**32.8** featuring a C18:1 lipid chain [142].

In 1993, Pinchuk reported a stereocontrolled synthesis of both enantiomers of analogues of edelfosine featuring a C18:1 mono-unsaturated lipid chain [142]. This synthesis starts from ᴅ-mannitol that was protected with 4-methoxybenzaldehyde to produce **32.1** (Figure 32) [143]. Then, the methylation of the two alcohol functions produced **32.2**. A regioselective reductive cleavage of the acetal was achieved by using either sodium cyanoborohydride and trifluoroacetic acid to yield the thermodynamic product **32.4** or sodium cyanoborohydride and trimethylchlorosilane (a bulkier reagent) that favor the formation of the kinetic product **32.3** (Figure 32). Then, a quite similar sequence (the order was different) can be applied to produce one of the enantiomers (*R*)-**32.8** or (*S*)-**32.8**. As exemplified for (*S*)-**32.8** in Figure 32, the oxidative cleavage of **32.4** with sodium periodate and the reduction of the aldehyde intermediate with sodium borohydride yields the alcohol **32.5** that was deprotonated with NaH and subsequently alkylated with oleyl alcohol mesylate. The deprotection of the primary alcohol with cerium salts, produced the key intermediate **32.6**. Finally, the phosphocholine group was installed via the use of the 2-chloro-2-oxo-1,2,3-dioxaphospholane (**32.7**) to produce (*S*)-**32.8**.

**Figure 33:**
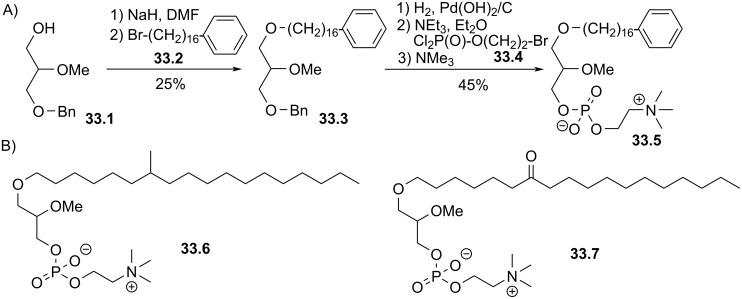
Synthesis of edelfosine analogues with modulation of the lipid chain; A) illustration with the synthesis of **33.4**; B) structures of **33.5** with a methyl group and **33.6** with a ketone [145].

The last two examples illustrated the incorporation of a mono-unsaturated lipid chain in the structure of edelfosine analogues. Another type of modification involved the lipid chains. In 1989 the group of Counsell reported analogues of edelfosine with an iodophenyl group positioned in ω-position of the lipid chain. These structures were designed for bio-imaging purposes [144]. In 1995, Mauleón et al. [145], extended this study by reporting the incorporation of a phenyl group in ω-position or by adding an alkyl group, a ketone or an alcohol function as illustrated in Figure 33. The idea of these modifications was to gain in selectivity because previous works demonstrated that for PAF a straight saturated alkyl chains was required for its biological effects [58]. The synthesis started by the deprotonation of the alcohol **33.1** that reacted with the bromoalkyl **33.2** that features a phenyl group in ω-position to produce **33.3**. Then, the debenzylation and the installation of the phosphocholine polar head group using 2-bromoethyl dichlorophosphate (**33.4**) produced **33.5**. Two analogues, among many others, were prepared following the same method featuring a branched lipid chain (**33.6**) or a lipid chain with a ketone function (**33.7**) as shown in Figure 33B.

**Figure 34:**
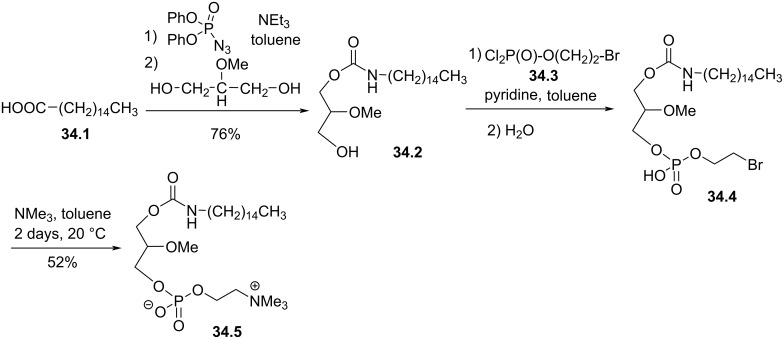
Synthesis of phospholipid featuring a carbamate function to link the lipid chain to the glycerol unit [146].

The replacement of the *sn*-1 ether linkage by a carbamate moiety was proposed by the company Takeda Chemicals Ind. [146–147]. The reaction starts with the conversion of the fatty acid (palmitic acid (**34.1**) in the example shown in Figure 34) into a reactive lipid isocyanate via the Curtius rearrangement with diphenylphosphoryl azide (DPPA). Then, the isocyanate generated in situ reacted with 2-methoxypropane-1,2-diol to produce the carbamate **34.2**. The use of a glycerol building unit already methylated in *sn*-2 position as precursor is interesting since it renders the synthesis more convergent. This approach is adapted when the stereocontrol of the *sn*-2 position is not required. Then, the phosphocholine polar head group was introduced in a two-step sequence by using 2-bromoethyl phosphorodichloridate (**34.3**) to give **34.5** in two steps. Following this synthesis scheme Tsushima et al. [147], also introduced different ammonium salts (e.g., pyridinium, thiazolium). This series of molecules were evaluated as antifungal compounds. The compounds featuring a C14 lipid chain and a phosphocholine polar head group were the most efficient compounds.

**Figure 35:**
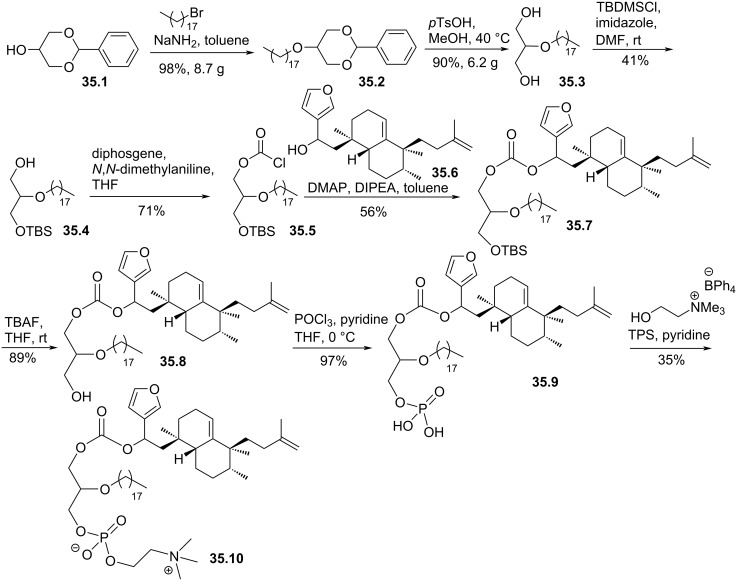
Synthesis of sesquiterpene conjugates of phospho glycero ether lipids [148].

The replacement of the alkyl chain present in *sn*-1 position by a sesquiterpene linked to the glycerol moiety via a carbonate tether and the incorporation of one unsaturated lipid chain via an ether function at the *sn*-2 position was described by Gil-Mesón et. al, in 2016 [148]. The goal of this study was to design sesquiterpene bioconjugates having a structure inspired from ether lipids like edelfosine. The strategy started from the protected glycerol **35.1** that was deprotonated with sodium amide and alkylated with bromooctadecane according to a Williamson reaction to produce **35.2** (Figure 35). After deprotection of the primary alcohol function, **35.3** was monoprotected with *tert*-butyldimetylsilyl (TBS) to produce **35.4** that was treated with diphosgene in the presence of *N*,*N*-dimethylaniline providing the intermediate **35.5** featuring a chloroformate moiety at the *sn*-1 position. Then, the sesquiterpene moiety **35.6** was incorporated by the reaction of its alcohol function with the chloroformate **35.5** in the presence of DMAP and DIPEA. To finalize the synthesis, the TBS protecting group was removed and, then, the phosphocholine polar head group was installed by using the POCl_3_ method. The phosphate intermediate **35.9** received the choline group in a reaction with choline tetraphenylborate and 2,4,6-triisopropylbenzenesulfonyl chloride (TPS) acting as a coupling agent providing the sesquiterpene conjugate derivative **35.10**.

**Figure 36:**
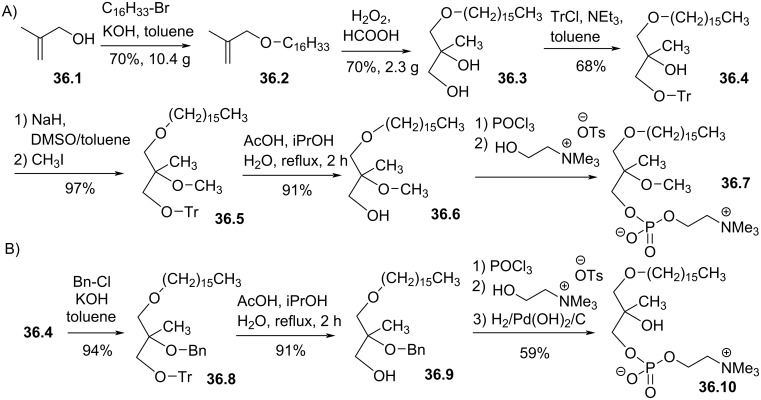
Racemic synthesis of methyl-substituted glycerol analogues **36.7** and **36.10**: A) synthesis of diether **36.7**; B) synthesis of lyso-analogue **36.10** [149].

**Modification at the *sn*-2 position:** Different modifications at the position *sn*-2 of the glycerol were reported. Bittman et al. reported in 1987 the synthesis of one analogue of edelfosine featuring one additional methyl group on the glycerol moiety as illustrated by the compounds **36.7** and **36.10** (Figure 36) [149]. The aim of the synthesis of these compounds was to evaluate the consequences of steric hindrance on the biological activity. They reported that **36.10** was neither an inhibitor of acetyl-CoA-dependent acyltransferase nor a toxic compound against human leukemic cells (HL-60). However, the methoxy analogue **36.7** exhibited high cell toxicity against HL-60 thus emphasis the importance of the methoxy group on cancer cell cytotoxicity. The synthesis of **36.7** and **36.10** starts with the alkylation of 2-methylprop-2-enol (**36.1**) to produce **36.2**. The bis-hydroxylation of the C–C double bond was achieved with hydrogen peroxide in the presence of formic acid to produce **36.3**. Then, tritylation of the primary alcohol produced **36.4** which is an intermediate for the synthesis of both **36.7** and **36.10**. For the synthesis of **36.7**, the tertiary alcohol was methylated to produce **36.5**. Then, the detritylation and the installation of the phosphocholine polar head group produced **36.7**. For the synthesis of **36.10**, the benzyl ether **36.8** was prepared and then, the trityl protecting group was removed. Then, the installation of the phosphocholine moiety and the debenzylation produced **36.10** featuring an unsubstituted alcohol.

**Figure 37:**
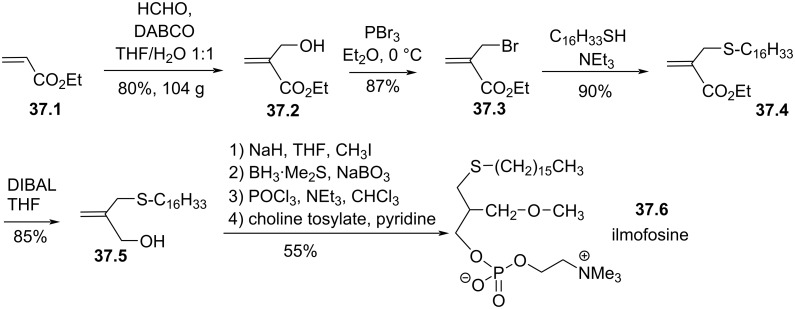
Racemic synthesis of ilmofosine (**37.6**) [155–156].

Ilmofosine is an analogue of edelfosine featuring two structural modifications. First, the C16 lipid chain is attached via a thioether linkage instead of an ether linkage for edelfosine. The second is related to the presence of a methoxymethyl fragment instead of a methoxy group present in edelfosine. Ilmofosine was tested in a phase I clinical trial and proved to be acceptable at a dose of 450 mg/m^2^ but no effect on the solid tumor was noticed [150] and a phase II clinical trial in non-small cell bronchogenic carcinoma had no effect on tumor regressions [151]. This compound is also efficient to prevent in vitro degranulation of mast cells [152]. Of note, the enrichment of plasma membrane with polyunsaturated lipids (e.g., DHA) increased the sensibility of leukemic cells to ilmofosine [153]. The first synthesis of ilmofosine [154] was improved by Bittman et al. [155] by using 2-hydroxymethylacrylate **37.2** which was prepared on large scale from ethyl acrylate **37.1** (Figure 37) [156]. The bromination of **37.2** and the incorporation of the lipid chain via a nucleophilic substitution with hexadecylthiol produce the lipid derivative **37.4**. The reduction of the ester function to a primary alcohol produces **37.5**. The alcohol function was methylated with iodomethane after a deprotonation with sodium hydride. Then, the hydroboration and oxidative treatment with sodium perborate produced a primary alcohol. The installation of the phosphocholine group was achieved by using the POCl_3_ method to produce ilmofosine (**37.6**) in 37% overall yield.

**Figure 38:**
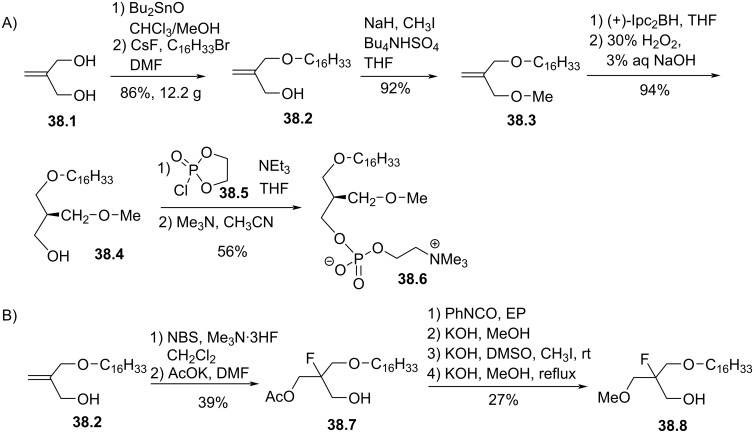
A) Stereoselective synthesis of **38.5** via a stereoselective hydroboration reaction; B) synthesis of fluorinated intermediate **38.8** [157–158].

The group of Bittman also reported a stereocontrolled synthesis of an oxygenated analogue of ilmofosine [157]. As shown in Figure 38A, the reaction started with the formation of a 1,3-cyclic stannoxane which is open and mono-alkylated to produce the ether lipid **38.2** on a large scale (12 g). Then, the methylation of the primary alcohol was achieved in the presence of tetrabutylammonium hydrogenosulfate as phase transfer catalyst to give **38.3**. The hydroboration of **38.3** with diisopinocampheylborane ((+)-Ipc_2_BH) produced **38.4** with high ee (84% ee). Then, the phosphocholine moiety was installed in 56% yield by using the method using 2-chloro-1,3,2-dioxaphospholane 2-oxide (**38.5**) to produce **38.6**. **38.6** and ilmofosine were evaluated as antiproliferative agent on epithelial cancer cell lines (MCF-7, A549, A427). Almost identical in vitro cytotoxicity was observed for ilmofosine and (*R*)- or (*S*)-**38.6** emphasis that both the chirality and the nature of the heteroatom (oxygen versus sulfur) have no influence on the cytotoxicity. The 2-fluoro analogue of **38.8** was reported by Burchardt et al. (Figure 38B) [158]. The key step is a bromofluorination of **38.2**. Then, the methylation of the alcohol function produced **38.8** which was subsequently functionalized with a phosphocholine polar head group.

**Figure 39:**
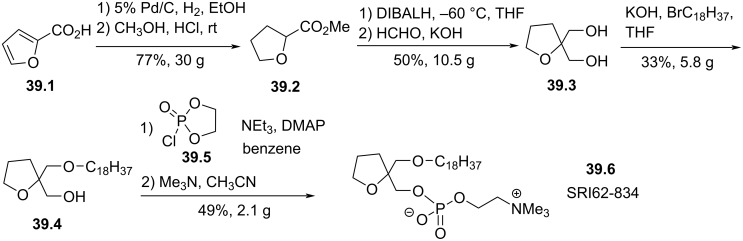
Racemic synthesis of SRI62-834 (**39.6**) featuring a spiro-tetrahydrofurane heterocycle in position 2 of the glycerol backbone [159].

The modification of the glycerol moiety by the incorporation of a tetrahydrofuran heterocycle was reported in 1987 by Houlihan et al. (Figure 39) [159]. 2-Furoic acid (**39.1**) was reduced into 2-tetrahydrofuroic acid and esterified to produce **39.2**. Then, the reduction of the ester function with DIBALH and the hydroxymethylation produced the diol **39.3**. The mono-alkylation of the diol produced in modest yield the ether lipid **39.4**. The installation of the phosphocholine polar head group, via the method of Chabrier et al. [94], produced SRI62-834 (**39.6**). It is reported that this compound featured similar cytotoxicity than edelfosine on cancer cell lines [136] and was also an inhibitor of PAF. Of note, the two enantiomers of SRI62-834 show the same cytotoxicity as the racemic form on HT29 and HL60 cancer cells [136].

**Figure 40:**
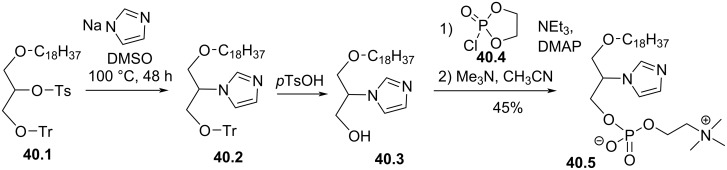
Racemic synthesis of edelfosine analogue **40.5** featuring an imidazole moiety in *sn*-2 position [160].

The installation of an imidazole or triazole heterocycle in *sn*-2 position in place of the secondary alcohol was reported in 1997 by H. K. Nair et al. (Figure 40) [160]. Starting from *rac*-1-*O*-*n*-octadecyl-2-*p*-toluenesulfonyl-3-*O*-tritylglycerol (**40.1**), the imidazole moiety was incorporated via a nucleophilic substitution (Figure 40). Then, the deprotection of the primary alcohol to **40.3** and the installation of the phosphocholine moiety produced the imidazole derivative **40.5**. The same protocol was used to prepare an analogue with a triazole moiety in position *sn*-2. These two analogues were tested as cytotoxic compounds against cancer cells. The authors reported that **40.5** and its triazole analogue featured similar cytotoxicity against MDA-MB-231, HL60 or HT29 than edelfosine (IC_50_ = 2–6 µM).

**Figure 41:**
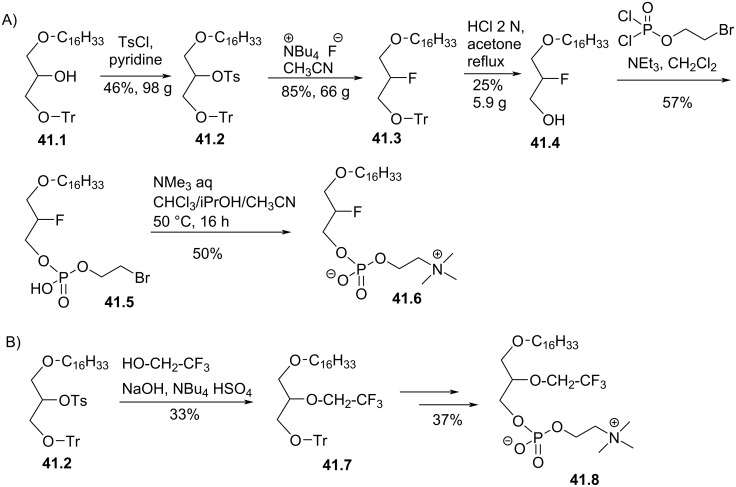
Racemic synthesis of fluorine-functionalized EL: A) Synthesis of **41.6** and B) synthesis of **41.8** [161–163].

The replacement of the methoxy group in *sn*-2 position of edelfosine by a fluorine atom was reported by Brachwitz et al. in 1982 (Figure 41A) [161] Starting from glycerol ether lipid **41.1**, the secondary alcohol was activated with a tosyl moiety as leaving group. Then, the fluorine atom was introduced by nucleophilic substitution using tetrabutylammonium fluoride to give **41.3**. After removing the trityl group to yield **41.4** [162], the phosphocholine moiety was introduced in two steps using 2-bromoethyl dichlorophosphate as reagent [161]. **41.6** was tested as antiproliferative compounds against Ehrlich ascites carcinoma cells and with an IC_50_ of 7 µM was a little less efficient than edelfosine (IC_50_ = 4.5 µM). The same synthetic route was used by Brachwitz et al. to place a 2,2,2-trifluoroethyl group in the *sn*-2 position (Figure 41B) [163]. The incorporation of the trifluoroethyl group was achieved by reaction of the ether lipid **41.2** with 2,2,2-trifluoroethanol in the presence of sodium hydroxide and with tetrabutylammonium salt as phase transfer catalysis (PTC). **41.8** exhibited almost the same cytotoxicity as **41.6** on Ehrlich ascites carcinoma cells with IC_50_ = 9.5 µM [164].

**Figure 42:**
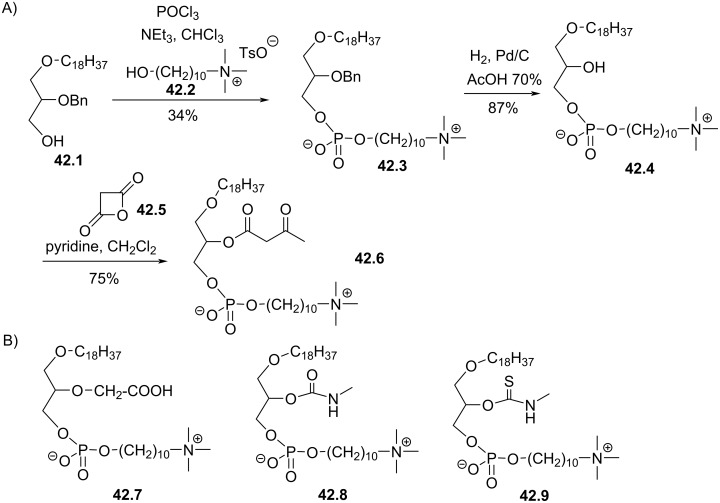
A) Synthesis of the β-keto-ester **42.6** that also features a decyl linker between the phosphate and the ammonium salt; B) structures of three further analogues **42.7**, **42.8**, and **42.9** prepared from the same substrate **42.4** [165].

In 1989, Nomura et al. reported the synthesis of ether lipids with a double modification on the glycerol moiety [165]. The authors introduced different modifications in position 2 of the glycerol including a keto ester (**42.6**, Figure 42A), a carboxylic acid function (**42.7**, Figure 42B) or a carbamate or thiocarbamate moiety (**42.8** and **42.9**). In the meantime, position 3 of the glycerol is constituted by a zwitterionic group with a decyl chain acting as a linker between the phosphate and the ammonium salt. The synthesis of one analogue is illustrated in Figure 42A. Starting from the alcohol **42.1**, the first step consists in the installation of the polar head group that uses phosphorus oxychloride and the ammonium salt **42.2** that was previously prepared from decane-1,10-diol. Then, **42.3** was debenzylated by catalytic hydrogenolysis to give **42.4**. Finally, the secondary alcohol opens the lactone of the ketene **42.5** to offer the analogue **42.6** with a β-keto ester moiety at position 2 of the glycerol.

**Modification at the *sn*-3 position:** The modifications in position *sn*-3 of the glycerol correspond to alternatives to the phosphocholine moiety or to introduce some structural alterations corresponding for instance to transform the zwitterion polar head group into a cationic moiety. Of note, the replacement of the zwitterion polar head group by a non-ionic polar group (e.g., saccharide) is mainly reported in the next section.

**Figure 43:**
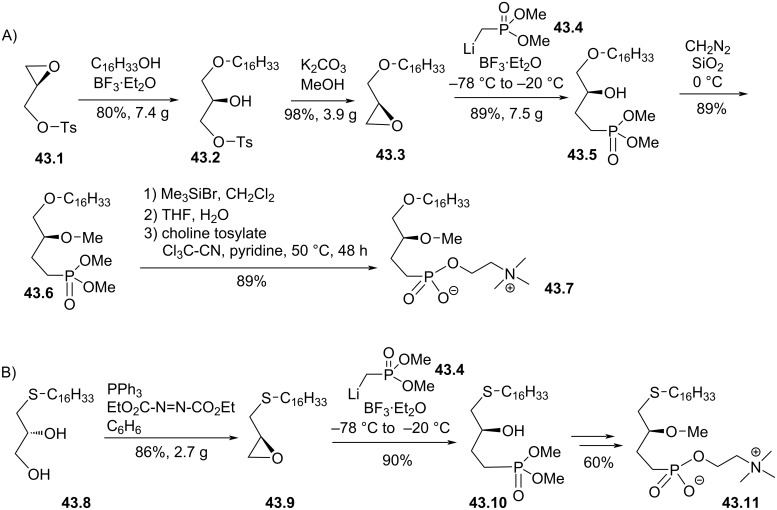
Synthesis of phosphonate-based ether lipids; A) edelfosine phosphonate analogue **43.7** and B) thioether analogue **43.11** [166–167].

With the aim to prepare ether lipids resistant to phospholipase C, the replacement of the phosphate group by a phosphonate was reported by R. Bittman et al. [166–167]. The enantioselective sequence starts with the addition of hexadecanol on glycidol tosylate **43.1** assisted by boron trifluoride (Figure 43A). The resulting secondary alcohol **43.2** placed in basic media produced the epoxide **43.3**. Then, the key step of the sequence involved the nucleophilic addition of the lithium salt of dimethyl methylphosphonate **43.4** on the epoxide **43.3** in the presence of boron trifluoride to give **43.5**. Diazomethane was employed to achieve the methylation of the secondary alcohol to form **43.6**. Then, the phosphonic acid function was prepared by reaction with bromotrimethylsilane according to the McKenna method [168]. Finally, **43.7** resulted from the coupling of the phosphonic acid function with choline tosylate that was promoted with trichloroacetonitrile according to the method of Rosenthal [169–170]. The synthesis of the thio-analogue **43.11** (Figure 43B) used the thioether **43.8** as starting material that was previously prepared from glycidol and hexadecylthiol [169]. The epoxide **43.9** was prepared by a Mitsunobu reaction. Then, the incorporation of the dimethyl phosphonate and the installation of the choline moiety used the same methodology than for **43.6** and **43.7**. The cytotoxicity of **43.7** and **43.11** was comparable to those of edelfosine on WEHI-3B cells. In vivo, these two compounds prolonged the survival of CD1 mice implemented with L1210 tumors.

**Figure 44:**
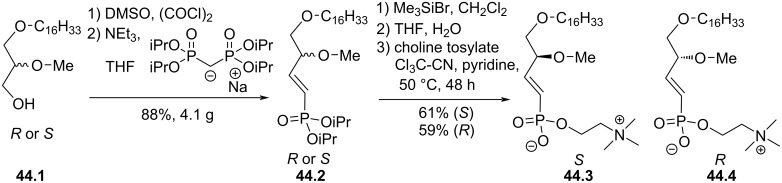
Enantioselective synthesis of phosphonates **44.3** and **44.4** [171].

Another strategy to introduce a hydrolytically stable phosphonate function was reported by Bittman and Arthur in 2004 by using the Wadsworth–Horner–Emmons (WHE) reaction (Figure 44) [171]. Starting from the enantiomerically controlled diether **44.1**, a two-step one-pot synthesis combining Swern oxidation and WHE reaction produced the vinylphosphonate **44.2** (*R* or *S*). The transformation of the phosphonate to phosphonic acid with bromotrimethylsilane and then the coupling of the phosphonic acid with the choline moiety produced the enantiopure derivatives **44.3** or **44.4**. Interestingly, a singularity of these two enantiomers is that they exhibit different biological effects on PKB (protein kinase B) while similar efficacy was reported on MAPK (mitogen-activated protein kinase) and JNK (c-Jun-NH_2_-terminal kinase).

**Figure 45:**
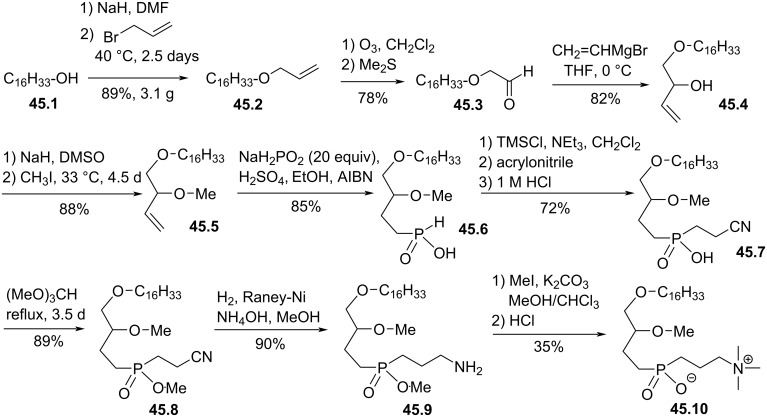
Racemic synthesis of phosphinate-based ether lipid **45.10** [172].

Still with the aim to limit the action of phospholipase, Regan and Markoulides reported in 2015 the replacement of the phosphate group by a phosphinate moiety meaning that the phosphorus atom is bonded to two stable P–C bonds thus rendering the molecule resistant to both phospholipase C and D [172]. The sequence starts from hexadecanol, which was allylated to **45.2** (Figure 45). Then, the ozonolysis of **45.2** in reductive medium produced aldehyde **45.3**. The nucleophilic addition of vinylmagnesium bromide produced **45.4** that was methylated on the secondary alcohol to give **45.5**. The radical addition of sodium hypophosphite, used in excess, produced in good yield the phosphinic acid **45.6**. The double silylation of **45.6** produced a phosphorus(III) intermediate that reacted with acrylonitrile (Michael addition) and produced, after hydrolysis of the remaining silylphosphinate, **45.7**. The methylation of **45.7** with trimethyl orthoformate produced the methyl phosphinate **45.8**. Then, the reduction of the nitrile into primary amine **45.9** was achieved by hydrogenation in the presence of Raney-nickel catalyst. Finally, the per-methylation of the primary amine and the hydrolysis of methylphosphinate produced the zwitterion **45.10**. Of note, the demethylation of the phosphinate was also achieved in the presence of iodotrimethylsilane but in that case the demethylation of the methoxy group was also observed producing a lyso-phosphinate derivative. The biological evaluation of **45.10** was not reported.

**Figure 46:**
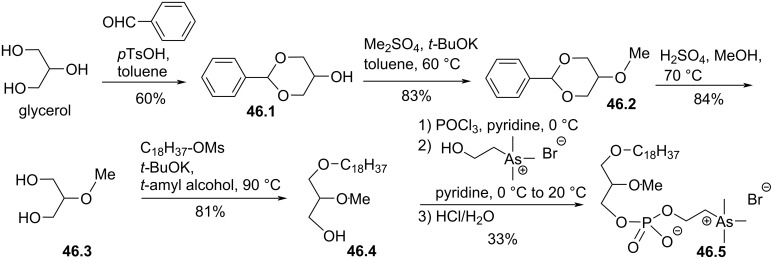
Racemic synthesis of edelfosine arsonium analogue **46.5** [173].

As reported above, in vivo and/or clinical trials revealed that edelfosine and different analogues exhibited elevated toxicity. One strategy, reported by Stekar et al. [173], investigated a modification that consisted in the replacement of the phosphocholine polar head group of edelfosine by a phospho-arsonium polar head group. The synthesis starts from glycerol that was protected as 1,3-benzylideneglycerol (**46.1**, Figure 46). Then, the methylation of the alcohol function was achieved with dimethyl sulfate to give **46.2**, which was subsequently deprotected to give 2-*O*-methylglycerol (**46.3**). This compound was alkylated with an octadecyl mesylate via a nucleophilic substitution to produce **46.4**. Then, the zwitterion polar head group was introduced by reaction of **46.4** successively with POCl_3_ in the presence of pyridine, with arsonium choline bromide and hydrolysis to produce **46.5**. The toxicity of **46.5** was studied and compared to edelfosine. In vitro toxicity revealed almost identical toxicity but acute in vivo toxicity assays indicated that **46.5** was less toxic than edelfosine (the LD_50_ is 1.7 mmol/kg for **46.5** and 0.8 mmol/kg for edelfosine). The low toxicity of arsonium-based cationic lipids was also confirmed later in other studies dedicated to the development of gene synthetic carriers [174–175].

**Figure 47:**
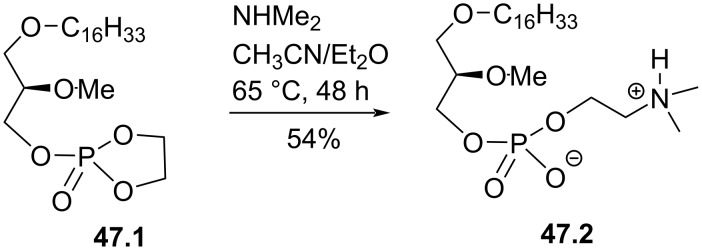
Synthesis of edelfosine dimethylammonium analogue **47.2** [118].

The replacement of the trimethylammonium moiety of edelfosine by a dimethylammonium was reported by Hajdu and Bhatia and follows the same methodology used for the synthesis of edelfosine [118]. The difference occurs at the very last step by the reaction of the cyclic phosphate **47.1** with dimethylamine (Figure 47). This reaction suggests that the cyclic phosphate can be open by a series of amines or nucleophiles that could produce a series of edelfosine analogues.

**Figure 48:**
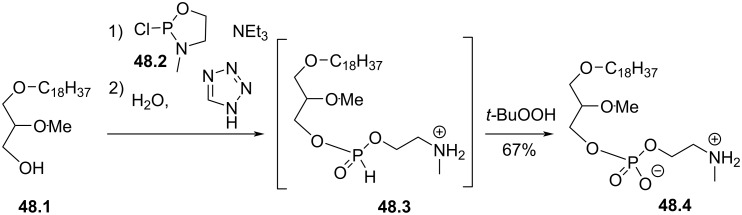
Synthesis of *rac*-C18-edelfosine methylammonium analogue **48.4** [176].

In line of the modification of the amine moiety of the polar head group of edelfosine, Lemmen and Stumpf [176] reported in 1990 a strategy to introduce a *N*-methylphosphoethanolamine moiety (Figure 48). The reaction of the diether **48.1** with 2-chloro-3-methyl-1,3,2-λ^3^-oxazaphospholane (**48.2**) in the presence of triethylamine followed by the opening of the heterocyclic intermediate with water in the presence of tetrazole produced the intermediate **48.3** featuring a dialkyl phosphite function. Finally, **48.3** was oxidized in situ with *tert*-butyl hydroperoxide to give **48.4**.

**Figure 49:**
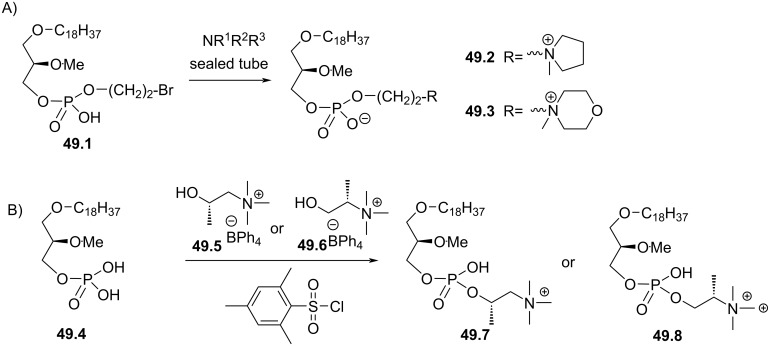
A) Synthesis of edelfosine *N*-methylpyrrolidinium analogue **49.2** or *N*-methylmorpholinium analogue **49.3** cations; B) illustration of the synthesis of ether lipids with methylphosphocholine polar head group [177].

The replacement of the trimethylammonium moiety, which is present in the phosphocholine polar head group of edefosine, with a modified ammonium salt was reported, for instance, by Fournier et al. in 1994 [177]. Starting from the enantiopure phosphate **49.1** (Figure 49A), the ammonium salts **49.2** and **49.3** were obtained by reaction with either *N*-methylpyrrolidine or *N*-methylmorpholine used in excess (the yields of this reaction were not specified). In the meantime, these authors reported a ramification of the ethylene linker between the ammonium and the phosphate function with a methyl group to prepare analogues with a methylcholine polar head group. For that purpose, the synthesis used the phosphate **49.4** as starting material which was previously reported by Hong et al. [178]. The coupling between **49.4** and the methylcholine **49.5** or **49.6** proceeded in the presence of mesitylsulfonyl chloride. Interestingly, **49.2** and **49.3** presented similar or higher cytotoxicity than edelfosine ((*R*)-, (*S*)- or *rac*-edelfosine) on the three leukemic cell lines tested (CEM, HUT78, and Namalwa) [177]. Regarding the methylcholine derivatives, the presence of the methyl in alpha position to the nitrogen atom (**49.8**) was more cytotoxic than in β-position (**49.7**).

**Figure 50:**
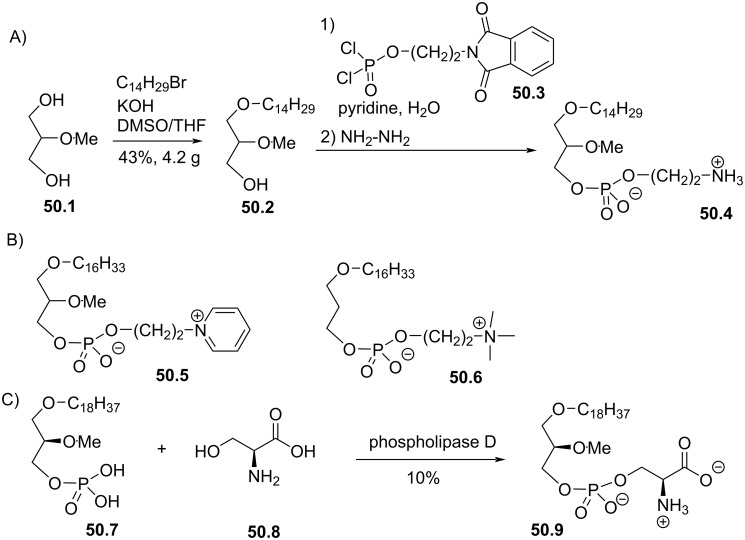
A) Synthesis of edelfosine’s analogue **50.4** with a PE polar group; B) illustration of a pyridinium derivative **50.5** and a desoxy analogue **50.6**; C) enzymatic synthesis of edelfosine phosphatidylserine analogue **50.9** [179–180].

The replacement of the phosphocholine (PC) polar group of edelfosine with phosphoethanolamine (PE) or phosphatidylserine (PS) was reported. For the PE derivative [179] the synthesis starts from 2-*O*-methylglycerol (**50.1**, Figure 50A) that was alkylated with 1-bromotetradecane to produce **50.2**. Then, the incorporation of the polar head group required two steps. First, 2-phthalimidoethyl phosphorodichloridate (**50.3**) was reacted with **50.2** and then water was added to hydrolyze the remaining P–Cl bond. Finally, the primary amine was deprotected with hydrazine to give **50.4**. Of note, in this publication, Nomura et al. have also reported analogues with a variation of the lipid chain length and have also replaced the trimethylammonium group with a pyridinium moiety as illustrated with **50.5**. They have also reported *sn*-2 desoxy analogues as illustrated with **50.6**. All these compounds were tested for their antimicrobial activities. For the PS derivative, Brachwitz et al. [180] reported an enzymatic method to prepare phosphatidylserine analogue **50.9**. **50.9** was tested as inhibitor of Ehrlich ascites tumor cell growth (IC_50_ = 30 µM).

**Figure 51:**
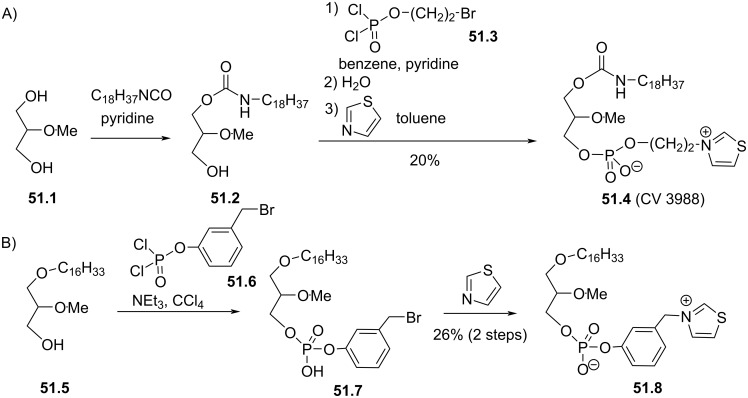
A) Synthesis of **51.4** featuring a thiazolium cationic moiety; B) synthesis of thiazolium-based EL **51.8** [146,181].

In 1983, Takeda Chemicals reported the synthesis and the biological effect (inhibitor of PAF) of **51.4** (CV-3988) that features a methoxy group in position 2 of glycerol, an octadecyl lipid chain linked to glycerol via a carbamate function and a polar group constituted by a phosphate and a thiazolium moiety (Figure 51A) [146]. The synthesis of **51.4** started with the incorporation of the lipid chain via the formation of a carbamate function (**51.2**). Then, the installation of the polar group used 2-bromoethyl dichlorophosphate (**51.3**) to introduce the phosphate moiety and then the reaction with thiazole produced the thiazolium compound **51.4** (CV 3988). The lipid diether with a thiazolium moiety was also reported in 1992 by Wissner et al. (Figure 51B) [181]. These analogues are also featuring an aromatic ring between the thiazolium and the phosphate moiety. Starting from the diether **51.5**, the reaction with the dichlorophosphate **51.6** [182] produced intermediate **51.7**. Then, the reaction with thiazole produced the thiazolium **51.8**.

**Figure 52:**
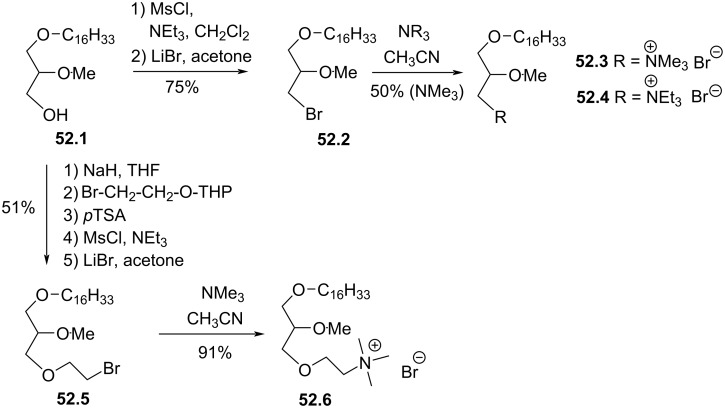
Synthesis of cationic ether lipids **52.3**, **52.4** and **52.6** [135,183].

After having illustrated the modification of the cationic moiety present in edelfosine, another series of compounds consisted in removing the phosphate group, thus leading to cationic ether lipids or protonable ether lipids. In 1990, E. J. Modest et al. reported the synthesis of a series of cationic ether lipids starting from the diether **52.1** (Figure 52) [183]. The transformation of **52.1** into its bromide analogue **52.2** was achieved in a two-step sequence using mesylation and reaction with lithium bromide [135]. Finally, the introduction of the ammonium salt was achieved by reaction with either trimethylamine in a sealed tube or with triethylamine to give respectively **52.3** and **52.4**. With the aim to keep present the oxygen atom in position 3 of the glycerol, the introduction of a 2-bromoethyl moiety was achieved to prepare **52.5** in a five-step sequence. Then, the same reaction protocol producing the ammonium salts was applied to give **52.6** [135]. Of note, a series of compounds having some structural analogy with **52.6** (the difference comes from the presence of an ester function in position 2 of glycerol; i,e., acetyl) were reported by Wissner et al. [182].

**Figure 53:**
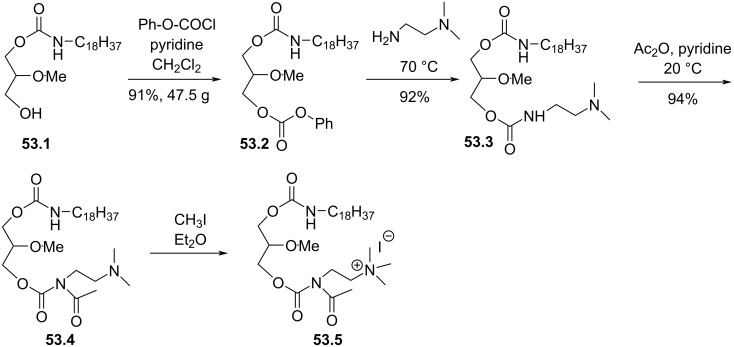
Synthesis of cationic carbamate ether lipid **53.5** [184].

Another series of compounds devoid of the phosphate group was reported by Takeda Chemicals Industries in 1989 [184]. The synthesis of one typical example, featuring a carbamate linkage between the lipid chain and glycerol, a methoxy group in position 2 of glycerol and an ammonium salt linked via an acetylated carbamate function in position 3 of glycerol, is presented in Figure 53. The synthesis used **53.1** as starting material that was prepared according to a previous reported method [147]. **53.1** reacted with phenyl chloroformate to give the phenyl carbonate **53.2**. *N*,*N*-Dimethylethylenediamine was added to **53.2** to produce the carbamate **53.3**. Then, a regioselective acylation of the carbamate function present in position 3 of glycerol was achieved with acetic anhydride in the presence of pyridine. Finally, the methylation of the tertiary amine with iodomethane produced the ammonium salt **53.5** (the yield was not indicated for this step).

**Figure 54:**
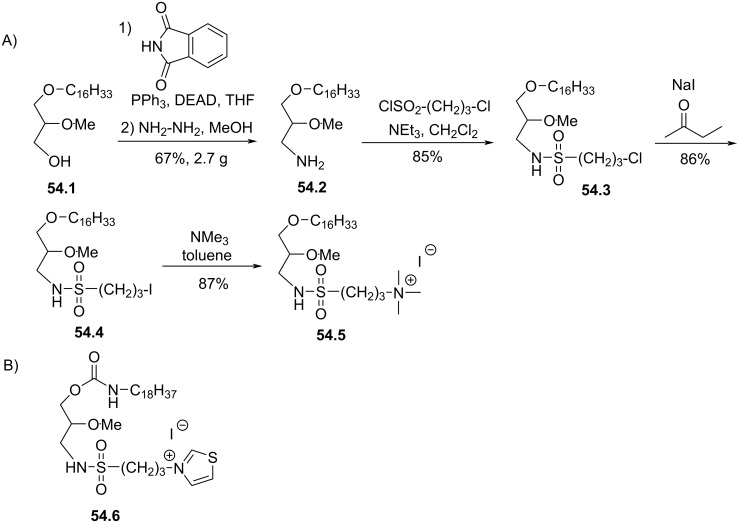
Synthesis of cationic sulfonamide **54.5** [185].

The replacement of the carbamate function that links the cationic moiety to the glycerol backbone in position 3 of compound **53.5** with a sulfonamide group was reported by Kamata et al. (Figure 54) [185]. This sequence started from the diether **54.1** that was transformed in the primary amine **54.2** by a sequence using a Mitsunobu reaction (to introduce the phthalimide moiety) followed by reaction with hydrazine. Then, the amine **54.2** reacted with 3-chloropropanesulfonyl chloride to produce **54.3**. The exchange of the chloride with iodide was achieved with sodium iodide in methyl ethyl ketone used as solvent. Finally, the reaction of **54.4** with trimethylamine produced **54.5**. It must be noted that the authors used **54.4** to introduce a thiazolium salt instead of the trimethylammonium moiety. In this paper, the authors also reported a large series of analogues of **54.5** in which the ether function present in position 1 of the glycerol was replaced by a carbamate function and with different cationic groups (e.g., thiazolium, pyridinium, quinolinium) located close to the sulfonamide tether as illustrated with **54.6**.

**Figure 55:**
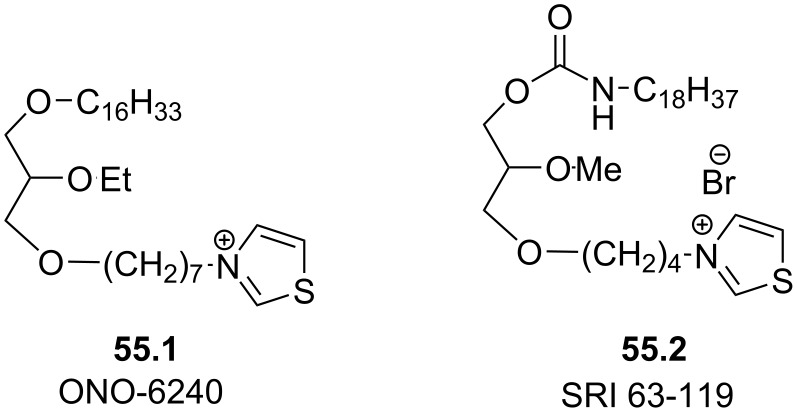
Chemical structure of ONO-6240 (**55.1**) and SRI-63-119 (**55.2**).

The compound ONO-6240 (**55.1**) and SRI-63-119 (**55.2**, Figure 55) developed respectively by ONO Pharmaceuticals and Sandoz are other examples of ether lipids without phosphate groups. To the best of our knowledge, their synthesis is not reported in the literature. These compounds were reported to be PAF antagonists [186–187].

**Figure 56:**
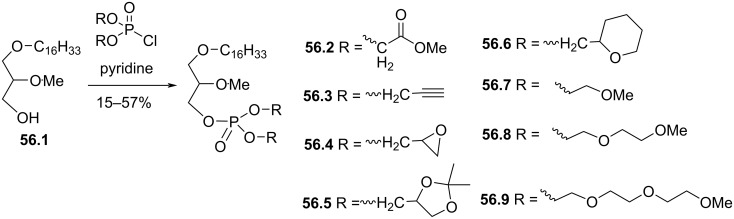
Synthesis of non-ionic ether lipids **56.2**–**56.9** [188].

The precedent figures illustrated the suppression of the phosphate moiety present in edelfosine thus producing cationic ether lipids. Another structural modification consisted in removing the cationic moiety and to substitute the phosphate function in order to isolate non-ionic ether lipids as reported by McGuigan et al. in 1995 [188]. These non-ionic ether lipids were prepared by using **56.1** as starting material. The reaction of **56.1** with the appropriate chlorophosphate in pyridine produced a series of ether lipids. Some of these compounds (**56.2**–**56.9**) are presented in Figure 56. This series of compounds were tested in vitro as inhibitors of the incorporation of tritiated thymidine in DNA of CNCM-I222 cells. For this in vitro assay, compound **56.9** exhibited the highest inhibition at 10 and 100 µM.

**Figure 57:**
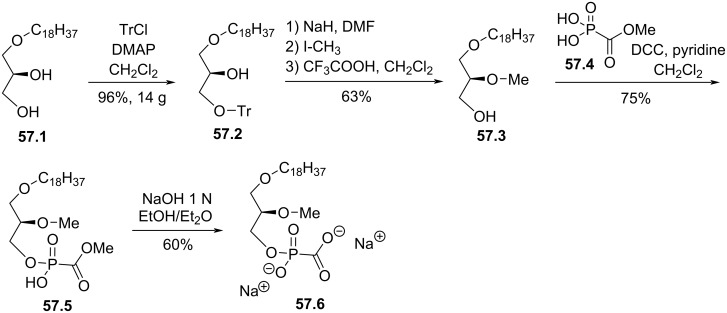
Synthesis of ether lipid conjugated to foscarnet **57.6** [189].

Another series of compounds came from the replacement of the phosphocholine moiety of edelfosine by fragments previously identified for their biological effects. The first goal of these hybrid structures was to combine the biological effect of ether lipids with those of another drug and more specifically antiviral drugs. In 1997, G. D. Kini et al. reported the conjugation of ether lipids with foscarnet [189]. For this purpose, the primary alcohol present in **57.1** was first protected with a trityl group to give **57.2** (Figure 57). Then, the secondary alcohol was methylated with iodomethane and the primary alcohol was deprotected with trifluoroacetic acid to give **57.3**. The coupling of **57.3** with methyl phosphonoformate **57.4** in the presence of DCC yields **57.5**. Finally, the saponification of the ester function produced the ether lipid conjugate **57.6** as its disodium salt.

**Figure 58:**
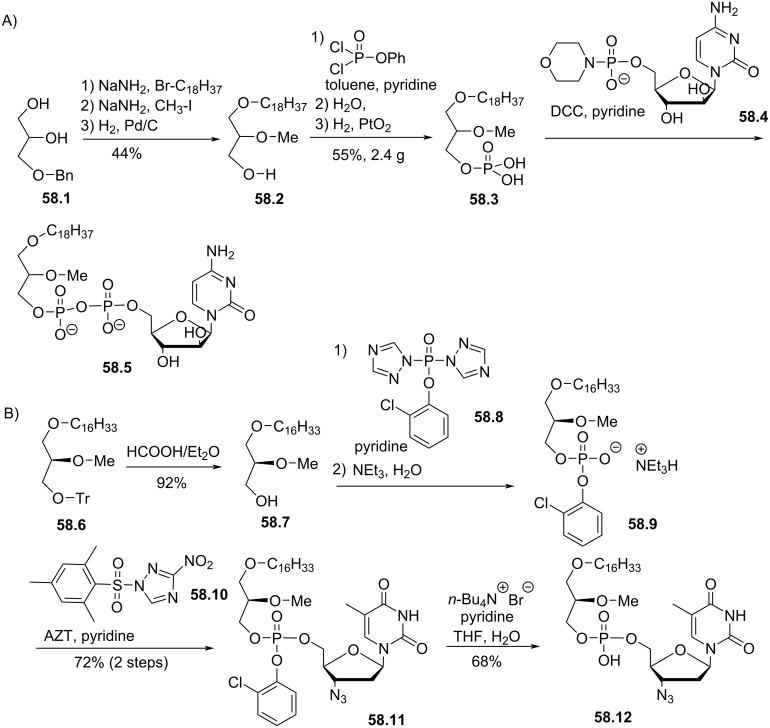
A) Synthesis of ether lipid conjugated to arabinofuranosylcytosine; B) synthesis of AZT conjugated to ether lipid [178,190].

W. E. Berdel et al. reported in 1986 the conjugation of ether lipids with nucleotides derived from arabinofuranosylcytosine [178]. As detailed in Figure 58A, benzylglycerol **58.1** was alkylated with bromooctadecane in the presence of sodium amide, methylated at the position 2 of the glycerol and finally the primary alcohol was deprotected by catalytic hydrogenolysis to give **58.2**. Then, the reaction of **58.2** with phenyl phosphonodichloridate followed by hydrolysis and hydrogenolysis to remove the phenyl group in the presence of PtO_2_ as catalyst produced **58.3**. The coupling reaction of **58.3** with 1-β-ᴅ-arabinofuranosylcytosine-5'-monophosphoromorpholidate **58.4** gives **58.5** with an unspecified yield. The incorporation of AZT (3’-azido-3’-deoxythymidine) directly bonded to the phosphate group present in edelfosine was reported by T. Mavromoustakos et al. in 2001 (Figure 58B) [190]. First, the authors used a method initially reported by Hajdu and Bhatia [118] (Figure 22) to prepare **58.6** in 49% yield from ʟ*-*glyceric methyl ester acetonide. Then, the trityl group of **58.6** was removed in acidic media to give **58.7**. The primary alcohol of **58.7** reacted with 2-chlorophenyl bis(1*H*-1,2,4-triazol-1-yl)phosphinate (**58.8**) in the presence of pyridine to give, after hydrolysis in presence of triethylamine, the phosphate salt **58.9**. Then, the coupling of **58.9** with AZT in the presence of 1-(mesitylene-2-sulfonyl)-3-nitro-1*H*-1,2,4-triazole (MSNT) used as coupling agent produced **58.11**. In the final step, the *O*-chlorophenyl group was removed by hydrolysis in the presence of pyridine to give **58.12**.

**Figure 59:**
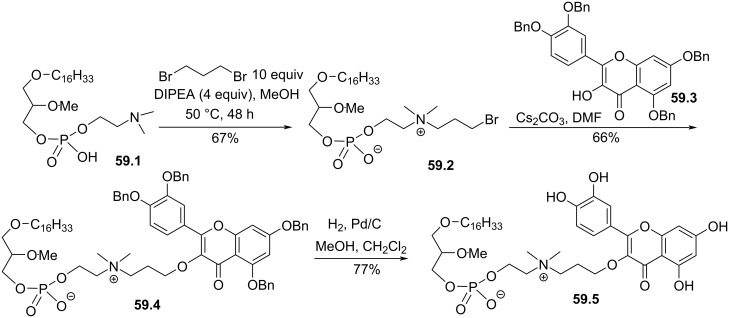
Synthesis of quercetin conjugate to edelfosine [191].

In 2019, Y. Xia et al. reported the conjugation of edelfosine to quercetin that belongs to flavonoids [191]. Quercetin is known for its antioxidant properties. The synthesis starts from the ether lipid **59.1** that was alkylated with an excess of 1,3-dibromopropane to produce **59.2** (Figure 59). Then, the incorporation of the benzyl-protected quercetin (**59.3**) via the formation of an ether linkage yields **59.4**. After debenzylation, the final ether lipid conjugate of quercetin **59.5** was isolated. Of note, compound **59.5**, at the difference of quercetin, was soluble in a mixture of methanol and dichloromethane. **59.5** was tested in vitro on a panel of cancer cells. The IC_50_ concentrations were lower for **59.5** than for edelfosine indicating an improved antiproliferative activity. Although edelfosine exhibited a membrane damage as revealed by lactate dehydrogenase released, this situation was not observed for **59.5**. The authors reported that **59.5** induced apoptosis.

### 3 Saccharide derivatives of glycero ether lipids

The interest in glyco ether lipids increased over the last 20 years. This family of compounds that features a glycerol ether lipid backbone and at least one saccharide moiety was also identified by some authors as glycosylated antitumor ether lipids (GAELs). To the best of our knowledge this acronym was introduced by R. Bittman et al. in 1996 [192]. Nevertheless, if most of the biological studies of this type of compounds are currently focused on cancer treatment applications, it would not be surprising that other applications could emerge. In this section we have included the synthesis of ether lipids featuring at least one saccharide unit. Accordingly, the two coming sub-sections correspond to the incorporation of a saccharide moiety either in positions 2 and 3 of glycerol. Of note, the phosphocholine moiety present in edelfosine can still be present in the structure of the saccharide analogues (e.g., Glc-PAF) or absent (e.g., ohmline). The analogues possessing either an inositol or an aminoglycoside moiety are presented in the subsequent sections.

#### 3.1 Saccharide unit in position 2 of glycerol

**Figure 60:**
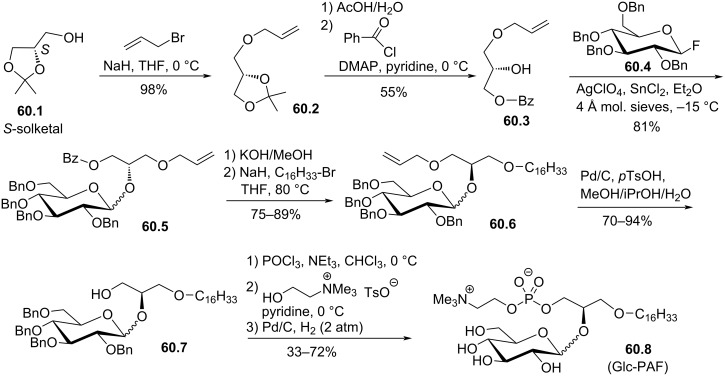
Synthesis of **60.8** (Glc-PAF) [194].

As reported above, edelfosine and some analogues were identified for their anticancer properties or for their utility for other pathologies. Unfortunately, the toxicity of these compounds has limited applications. With the aim to propose new analogues with reduced toxicity or side effects, the groups of J. Mulzer and W. Reutter, have reported in 1995 the incorporation of a saccharide unit in the structure of amphiphilic compounds that still featuring a phosphocholine moiety. First, they have reported a 1-acylglycero-*sn*-3-phosphocholine derivative with a glucose moiety in *sn*-2 position (Glc-PC). They have shown that this compound was a non-toxic (at 10 µM) inhibitor of cell proliferation and that the antiproliferative properties were not mediated by protein kinase C [193]. Then, they reported one analogue of Glc-PC but featuring one ether function in *sn*-1 position instead of an acyl linkage (Figure 60) [194]. This synthesis starts from (*S*)-solketal (**60.1**), which was alkylated with allyl bromide to produce **60.2**. Then, the deprotection of the acetal and the protection of the primary alcohol as a benzoic ester produced **60.3**. The glycosylation reaction involving the secondary alcohol and tetrabenzyl-β-ᴅ-glucopyranosyl fluoride (**60.4**) produced **60.5**. The saponification reaction liberates the primary alcohol that was alkylated with different alkyl bromides including 1-bromohexadecane as illustrated in Figure 60 to afford **60.6**. The alcohol function protected with an allyl group was deprotected in the presence of palladium used as catalyst to yield **60.7**. Then, the installation of the phosphocholine polar head group and the debenzylation of the protected alcohol functions of glucose produced the glucose analogue of PAF **60.8** (Glc-PAF) (the acetyl group of PAF was replaced by the glucopyranosyl moiety). Of note, a review dedicated to the biological effects of Glc-PC and **60.8** (Glc-PAF) and other analogues was published by Danker et al. in 2010 [195].

**Figure 61:**
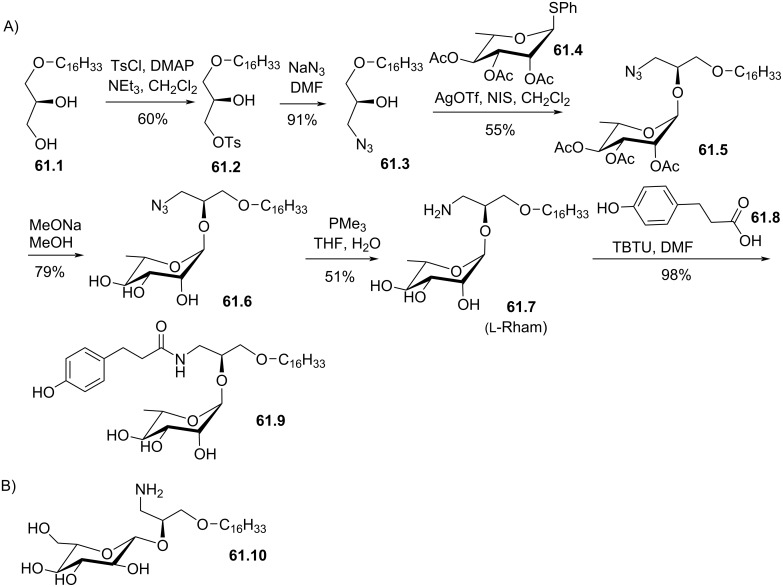
A) Synthesis of amino ether lipid **61.7** functionalized with a rhamnose unit and its amide analogue **61.9**; B) structure of ʟ-glucose-based derivative **61.10**, an analogue of **61.7** [196].

In 2020, the groups of G. Arthur and F. Schweizer from the University of Manitoba reported a series of ether lipids functionalized with an ʟ-rhamnose moiety [196]. As detailed in Figure 61, **61.1** was tosylated on the primary alcohol to give **61.2**. Then, the azide function was introduced with sodium azide in DMF to produce **61.3**. The glycosylation step used the method of B. Fraser-Reid [197] with the glycoside donor **61.4** and the acceptor **61.3** in the presence of *N*-iodosuccinimide (NIS) as coupling reagent and with silver triflate as catalyst. The resulting compound **61.5** was treated with sodium methoxide in methanol to remove the acetyl groups to give **61.6**. The application of Staudinger’s reaction conditions with trimethylphosphine as reagent produced the amino derivative **61.7**. Then, coupling of the amino group of **61.7** with carboxylic acid **61.8** with TBTU as coupling reagent produced amide **61.9**. In the meantime, the authors prepared **61.10** following a similar methodology to incorporate an ʟ-glucopyranoside moiety. **61.7** and **61.9** were tested as cytotoxic compounds on epithelial cancer cell lines. **61.7** proved to be the most efficient. Investigations related to the mechanism of action concluded that cell death was not due to a membrane lysis-based mechanism. In vivo studies on chicken embryo model of high-grade ovarian cancer proved the efficacy of compound **61.7** (ʟ-Rham) [198].

#### 3.2 Saccharide unit on position 3 of glycerol

**Figure 62:**
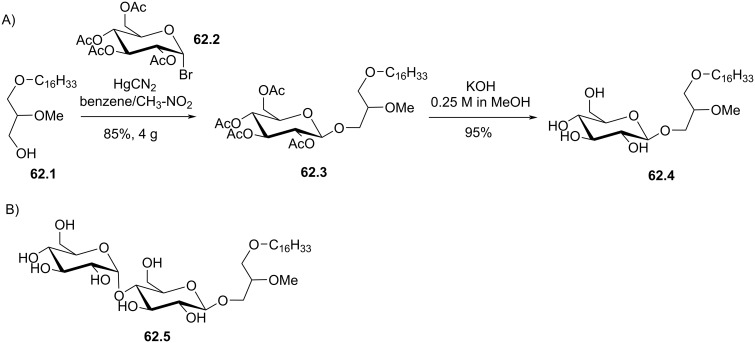
A) Synthesis of glucose ether lipid **62.4**; B) structure of ether lipid **62.5** possessing a maltose unit [205].

The synthesis of glycerol-based lipids functionalized with a saccharide moiety have interested many chemists from the beginning of the 70’s [199]. In the 80’s, the synthesis of glycerol monoether [200] or diether with two lipid chains [201–202] and with different saccharide moieties (e.g., glucopyranosyl [203], 2-desoxy-2-fluoromannopyranosyl [204]) was reported. One of the first studies that reports the synthesis of glycerol diether including a methoxy group in position 3 of the glycerol was published by N. Weber and H. Benning in 1986 [205]. The initial idea was to replace the ionic phosphocholine group present in the structure of edelfosine with non-ionic polar fragments. As shown in Figure 62A, the glycosylation reaction used the Knoenigs–Knorr method using acetobromo-α-ᴅ-glucose **62.2** and the ether lipid **62.1**. The acetylated β-ᴅ-glucopyranosyl ether lipid **62.3** was isolated and then, the acetyl groups were removed to give **62.4**. Of note, the same methodology was applied to prepare the disaccharide derivative **62.5** (Figure 62B) that features a maltose unit as neutral polar group. In this study, the authors have applied the synthesis from racemic or stereocontrolled substrate **62.2**. In a subsequent study, these authors reported that the analogue of **62.4** possessing an octadecyl lipid chain labeled with ^14^C was rapidly metabolized in vitro (Ehrlich ascites tumor cells). They concluded that the glycosidic bond was cleaved by the action of a β-glycosidase [206]. Of note, R. W. Franck and C. H. Marzabadi reported the analogue of **62.4** with a 2-desoxyglucose moiety [207–208].

**Figure 63:**
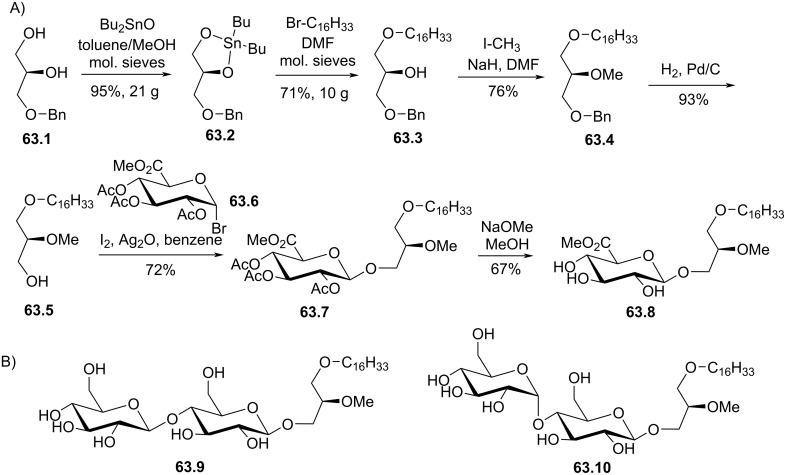
A) Synthesis of glucuronic methyl ester **63.8**; B) structure of cellobiose **63.9** and maltose **63.10** analogues prepared following the same protocol [209].

In 1991, the group of M. Liefländer reported the synthesis of neutral analogues of edelfosine with the replacement of the phosphocholine polar group with either glucuronic acid methyl ester **63.8**, cellobiose **63.9** or maltose **63.10** (Figure 63A and B) [209]. The synthesis (Figure 63A), illustrated with the preparation of glucuronic acid methyl ester **63.8**, started with the synthesis of the diether **63.5** which is a key intermediate. Its synthesis used 3-*O*-benzyl-*sn*-glycerol as starting material followed an unusual method because others, requiring protecting groups, did not produced satisfactory yields according to the authors. Here, the reaction of the diol **63.1** with dibutyltin oxide produced the tin acetal **63.2**. This compound reacted selectively in *sn*-1 position with 1-bromohexadecane to produce, after workup, the diether **63.3**. The next two steps were achieved following a previously reported protocol [71] to give **63.5**. Then, the last two steps were carried out using their own protocol published earlier to give **63.8** [200]. The cellobiose analogue **63.9** and the maltose analogue **63.10** were prepared following the same methodology.

**Figure 64:**
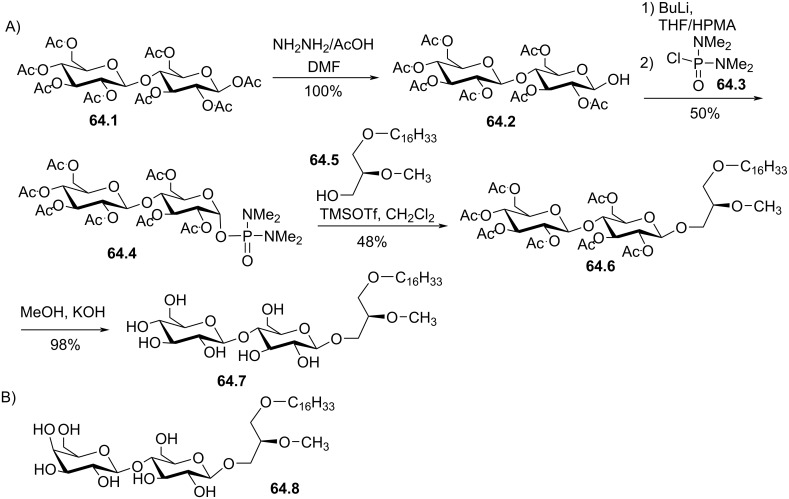
A) Synthesis of maltosyl glycerolipid **64.7**; B) structure of lactose analogue **64.8** prepared following the same protocol [210–211].

In 1994, the group of Bittman reported briefly the synthesis of **64.7** (no experimental parts) and in detail its biological effect that was compared to edelfosine [210]. The chemistry part of this work was more detailed in a second paper published in the same year [211]. They reported that edelfosine was an antineoplastic agent by limiting the number of colonies forming of murine WEHI-3B cells at 1 µM whereas **64.7** exerted any action up to 20 µM. They also reported that both compounds have a lysis effect on erythrocytes but with different kinetics and dose effect. The synthesis of **64.7** was inspired from a previous work reporting the synthesis of cerebrosides [212]. The anomeric acetyl group of peracetylated maltose **64.1** was quantitatively deprotected with hydrazine in acetic acid (Figure 64). Then, the deprotonation of the alcohol with butyllithium and the reaction of the alcoholate with tetramethyldiamidophosphorochloridate (**64.3**) produced the phosphorodiamidate **64.4** [212]. Then, the glycosylation of alcohol **64.5** with **64.4** catalyzed with trimethylsilyltriflate produced **64.6** [211]. **64.6** was deacetylated in methanol in the presence of potassium hydroxide to yield the maltose derivative **64.7**. The same methodology was applied to prepare **64.8** that features a lactose moiety in place of the maltose unit.

**Figure 65:**
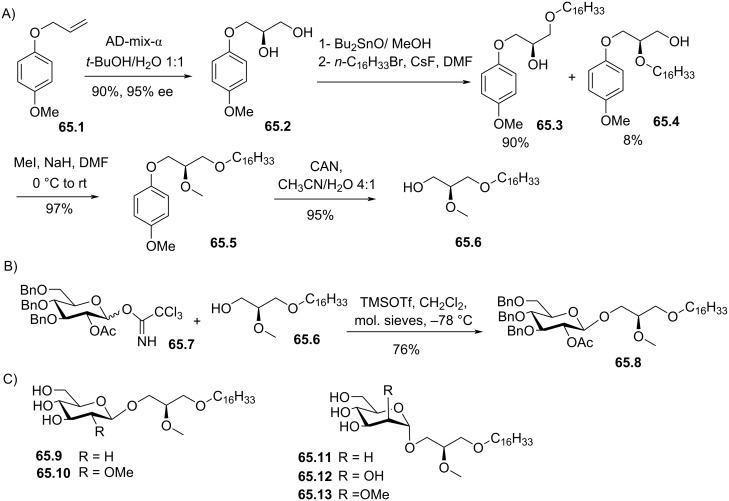
A) Asymmetric synthesis of the aglycone moiety starting from allyl 4-methoxyphenyl ether; B) glycosylation reaction to produce **65.8**; C) structure of glycol ether lipids [192].

The group of Bittman reported in 1996 a series of glycosylated antitumor ether lipids (GAELs) featuring either a glucose or a mannose moiety [192]. First, they reported an asymmetric synthesis of the aglycone part starting with the asymmetric bis-hydroxylation of allyl 4-methoxyphenyl ether **65.1** to produce **65.2** (Figure 65A). The opening of the 1,2-*O*-stannylidene intermediate, previously reported by Liefländer [209], is regioselective but it is not fully controlled leading to the formation of **65.3** (90%) and **65.4** (8%), required a separation by chromatography. Then, the methylation of the *sn*-2 alcohol function produced **65.5** and the deprotection of the primary alcohol with cerium(IV) ammonium nitrate (CAN) produced the key intermediate **65.6** in 95% yield. The saccharide units (glucose or mannose) were activated in anomeric position with a trichloroacetimidate group. The glycosylation reaction was achieved with trimethylsilyl trifluoromethanesulfonate (TMSOTf) used as catalyst and in the presence of molecular sieves (3 Å) (Figure 65B). The assistance of the adjacent acetyl group explained the formation of the β-anomer for the glucose derivative and α-anomer for mannose. For the glucose derivative (**65.8**), the authors removed the acetyl group in position 2 via the preparation of a xanthate and its reduction with tributyltin in the presence of AIBN, followed by debenzylation produced **65.9** (Figure 65C). The methylation of the intermediate secondary alcohol produced **65.10**. By using the same protocol, the mannose derivatives **65.11**, **65.12** and **65.13** were characterized. From this study, the authors reported that **65.9** was more efficient than edelfosine to inhibit the growth of MCF-7 human breast cancer cells. All the other compounds were less efficient.

**Figure 66:**
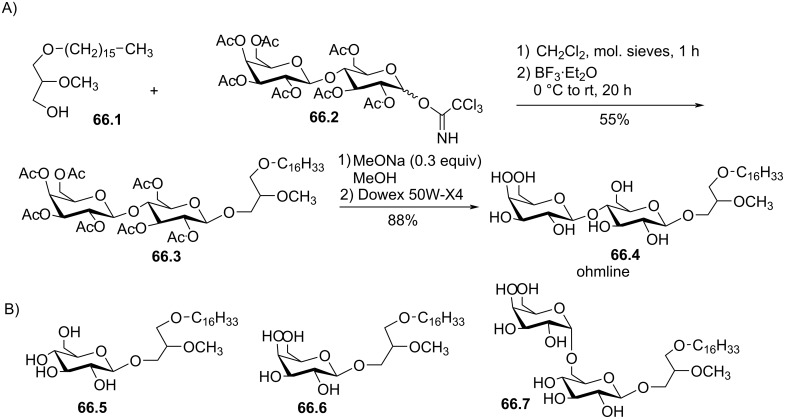
A) Synthesis of ohmline possessing a lactose moiety. B) Structure of other glyco glycero lipids prepared following the same method [41].

In 2011, the group of C. Vandier have reported for the first time that edelfosine inhibited the activity of SK3/KCa2.3 channels at 1 µM which is a non-toxic concentration (toxicity was observed at a concentration higher than 3 µM) [213]. Nevertheless, with the aim to evaluate less toxic compounds, we reported the synthesis of analogues of edelfosine in which the phosphocholine polar head group was replaced by a mono or disaccharide unit [41]. As an example, the synthesis of **66.4** (also identified as ohmline) starts (Figure 66) from the racemic (or chiral) alcohol 1-*O*-hexadecyl-2-*O*-methylglycerol (**66.1**). The glycosylation proceeds with hepta-*O*-acetyllactose-1-*O*-trichloroacetimidate (**66.2**) in the presence of boron trifluoride etherate to produce **66.3** that features a β-glycosidic linkage. Then, the deprotection of the alcohol function with sodium methanolate in methanol produced ohmline in 88% yield. A similar protocol was applied to the mono and disaccharides thus producing compounds **66.5** (glucose), **66.6** (galactose) and **66.7** (melibiose). These compounds were able to inhibit the activity of SK3 ion channels [214] and to reduce SK3-dependent cancer cell motility. The most efficient one is **66.4** (ohmline) that reduced SK3 current by 73% at 10 µM and SK3-dependent cancer cell motility by 50% (24 h at 300 nM). Noteworthy, ohmline which is non-toxic at 10 µM is also one of the most selective agents for the inhibition of SK3 [50] with only a limited effect on the other isoforms SK1 (20% of inhibition at 10 µM) and no effect on SK2 and IKCa (SK4). It must be noted, that the chirality in position *sn*-2 of the glycerol has no effect on the modulation of SK3 in vitro [41]. Finally, ohmline was also tested in vivo and proved its efficacy to prevent the occurrence of bone metastasis in a breast cancer model (murine experiments) [42]. Of note, the incorporation of a pyrene fluorescent group in omega position of the lipid chain of ohmline was prepared following the same methodologies as employed to prepare ohmline [215].

**Figure 67:**
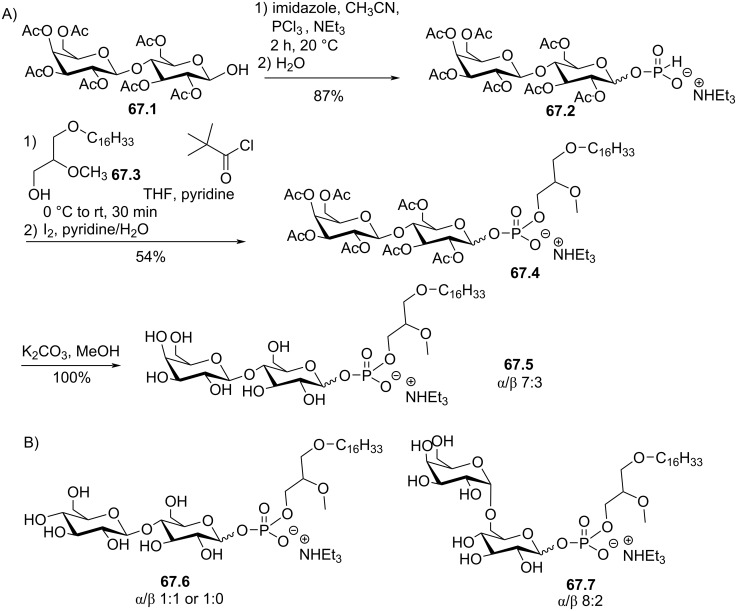
A) Synthesis of lactose-glycerol ether lipid **67.5**; B) analogues possessing a maltose (**67.6**) or melibiose moiety (**67.7**) [216].

With the aim to improve the water solubility of ohmline and also to evaluate the effect of a negative charge localized in the polar head group, we designed analogues possessing a phosphate moiety placed between glycerol and the saccharide or disaccharide unit [216]. The synthesis, illustrated with lactose in Figure 67A, consisted in first introducing the phosphate moiety at the anomeric position of lactose heptaacetate (**67.1**) by using phosphorus trichloride followed by a hydrolysis step to produce the salt **67.2**. The incorporation of the lipid moiety (1-*O*-hexadecyl-2-*O*-methyl-*rac*-glycerol **67.3**) was achieved via a mixed anhydride with pivaloyl chloride which is, for such type of lipid moiety, the best coupling agent [217]. Then, the di-substituted phosphite was oxidized with I_2_ in the presence of pyridine and water to produce **67.4**. Finally, deacetylation produced the lactose phosphate glycerol ether lipid **67.5**. A similar procedure was applied to prepare the maltose and melibiose derivatives **67.6** and **67.7** (Figure 67B). The lactose derivative **67.5** was indeed more water soluble than ohmline. This series of compounds exhibited almost the same activity than ohmline to reduce the activity of SK3 at 10 µM and to reduce SK3-dependent cell motility at 300 nM. This study also demonstrates that both the structure of the disaccharide unit and the stereochemistry of the glycoside linkage have, in this series of compounds, a limited incidence on the biological effects. More recently, we have reported via ^2^H NMR using deuterated ohmline that it has a tropism for membranes. This behavior is easily explained by its amphiphilic structure. Furthermore, the incorporation of ohmline in model membranes impact the order parameters especially for the rigid membrane featuring high ratios of cholesterol [218]. In addition, A. M. Bouchet, via molecular dynamics simulations reported that the presence of ohmline in model biomembranes induces a modification of the position of cholesterol [218]. Altogether, these results suggest that ohmline could act on SK3 channels function not via a direct interaction between SK3 proteins but via a local modulation of the biophysical properties of the plasma membrane. This selectivity of action (e.g., ohmline has no effect on SK2 channels) could be explained by the different interaction of membrane proteins with its lipid environment. Interestingly, we have also reported in the context of colon cancer cells that ohmline modifies the anti-EGFR mAbs action mediated by the control of calcium signaling [219].

**Figure 68:**
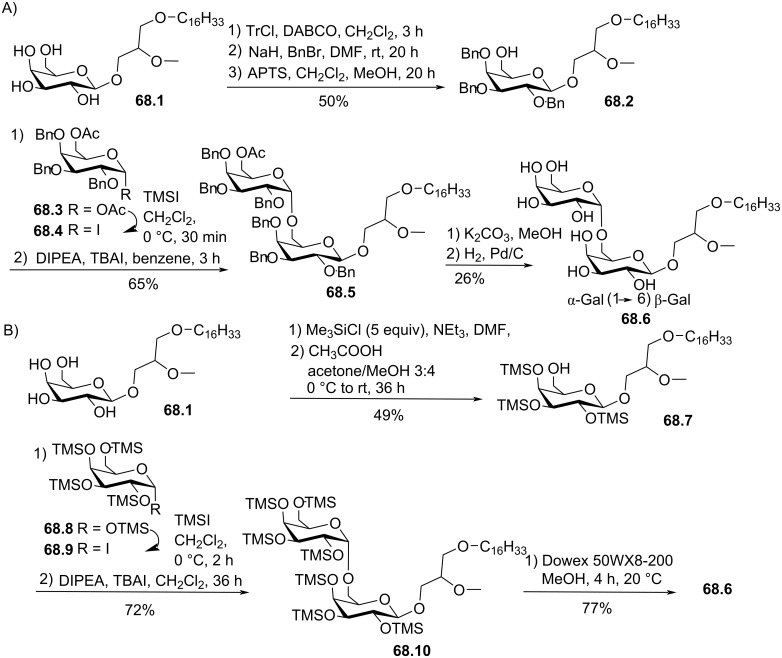
Synthesis of digalactosyl EL **68.6**, A) by using trityl, benzyl and acetyl protecting groups, B) by using trimethylsilyl as protecting group [221].

The incorporation of the (1→6)-digalactosyl moiety in place of the lactosyl group present in ohmline was inspired from a study reporting that some plants, under phosphate deprivation, induced the biosynthesis of (1→6)-digalactosyldiacylglycerol (DGDG) in place of phosphatidylcholinediacylglycerol [220]. We proposed two distinct syntheses for the preparation of digalactosyl ether lipids [221]. The common point of these two strategies was based on the glycosylation reaction of the galactosyl intermediate **68.1** (Figure 68). According to the first approach (Figure 68A), we used the classical protecting groups (benzyl, acetyl, trityl) to prepare in three steps the galactosyl derivative **68.2**. The second galactosyl unit **68.3** was prepared in two steps (56% yield) from the expensive commercial compound methyl α-ᴅ*-*galactopyranoside. Then, the anomeric position of **68.3** was transformed in α-iodo intermediate **68.4** that reacted with **68.2** to produce **68.5**. The acetyl and benzyl groups were removed to produce the (1→6)-digalactosylglycerol ether lipid **68.6**. Finally, **68.6** was isolated in 8 steps with a global yield of 5%. The second approach for the synthesis of **68.6** (Figure 68B), consisted in using trimethylsilyl as protecting group. Accordingly, **68.1** was per-silylated and then the silylated primary alcohol was deprotected with acetic acid in a mixture of acetone and methanol to produce **68.7**. The second galactosyl moiety **68.8** was prepared in one step (silylation) from galactose. The anomeric position was iodinated with trimethylsilyl iodide to produce **68.9** which was directly engaged in the coupling reaction with alcohol **68.7** to produce **68.10**. The deprotection of the seven alcohol functions was achieved by using an acidic resin to produce **68.6** in 77% yield. According to this second synthesis scheme, **68.6** was prepared in 5 steps in 25% global yield. The evaluation of **68.6** for the modulation of SK3 ion channels indicated that it was much less efficient than ohmline [221].

**Figure 69:**
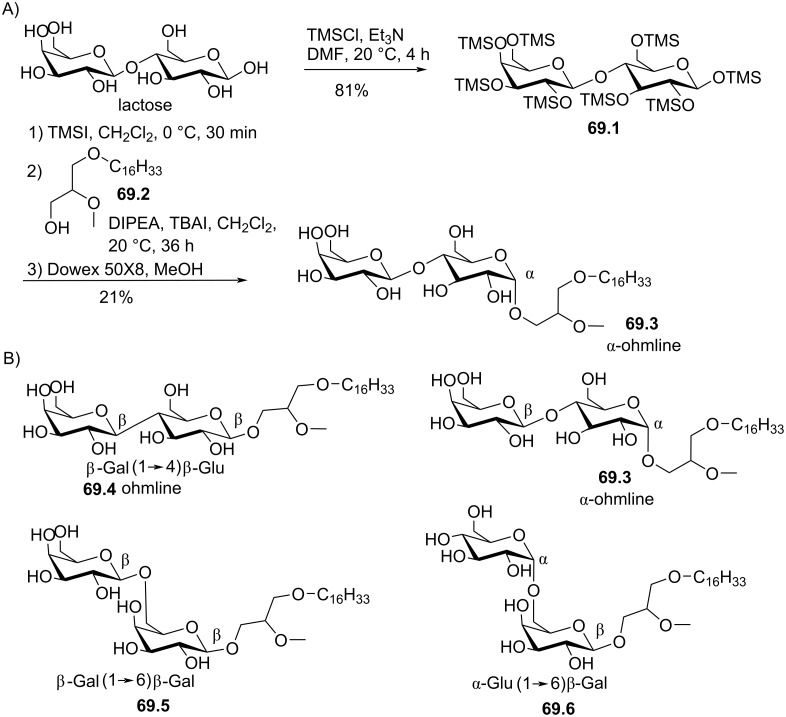
A) Synthesis of α-ohmline; B) structure of disaccharide ether lipids prepared by using similar methodologies [222].

The methodology using trimethylsilyl as protecting group was also employed to prepare another series of disaccharide-based ether lipids. This study aimed to evaluate the effect of (1→6) versus (1→4) disaccharide linkage, to study the stereochemistry of the glycosidic linkage for ohmline (α versus β linkage) and to study the effect of the order of the saccharide units (Glc-Gal versus Gal-Glc) [222]. As an example (Figure 69), α-ohmline was prepared by the per-silylation of lactose to produce **69.1**. Then, the mono-iodination at the anomeric position with trimethylsilyl iodide produced an electrophile that reacted with the lipid alcohol **69.2**. Then, the deprotection of the seven alcohol functions produced **69.3** (α-ohmline). The other molecules prepared in this study are presented in Figure 69B. The biological evaluation indicated that α-ohmline was completely inefficient to inhibit SK3. Among the other compounds, we found that **69.6** was as efficient as ohmline while **69.3** was inefficient. More recently, we replaced the oxygen atom localized at the *sn*-1 position of the glycerol unit of ohmline by a sulfur atom at the same position or close to this position. We found that some of these sulfur analogues of ohmline were activators of SK3 channels. This gain of function was applied to vasorelaxation assays [223].

**Figure 70:**
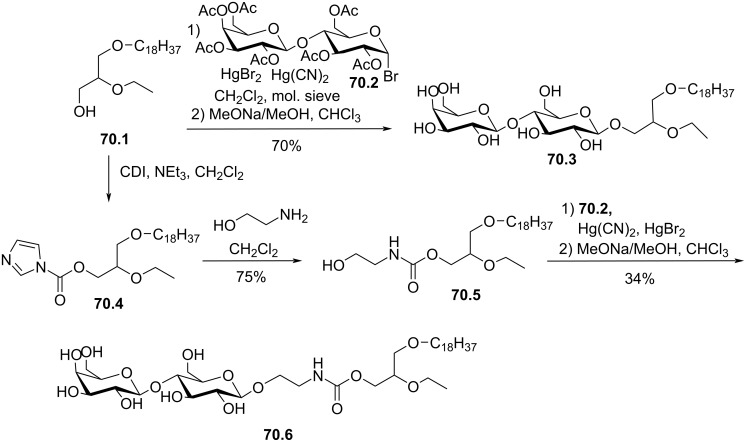
Synthesis of lactose ether lipid **70.3** and its analogue **70.6** featuring a carbamate function as linker [224].

In 2015, the group of N. G. Morozova reported in a communication the synthesis of analogues of ohmline in which the methoxy group in position 2 of glycerol was replaced by an ethoxy group and other derivatives with a carbamate linker between the glycerol and the saccharide unit [224]. The synthesis started from the diether **70.1** that was glycosylated with a bromide glycosyl donor like hepta-acetylated lactose **70.2** in the presence of mercury salts (Figure 70). Then, deacetylation produced **70.3**. A second approach consisted in adding a linker between the saccharide unit (e.g., lactose) and the glycerol moiety. To this end, **70.1** reacted with carbonyldiimidazole (CDI) to produce **70.4**. Then, the addition of amino-alcohol (e.g., aminoethanol) produced **70.5**. Finally, the glycosylation and the deprotection of the alcohol functions produced **70.6**. In this communication, the yield and spectroscopic characterizations of compound **70.4** are not reported.

**Figure 71:**
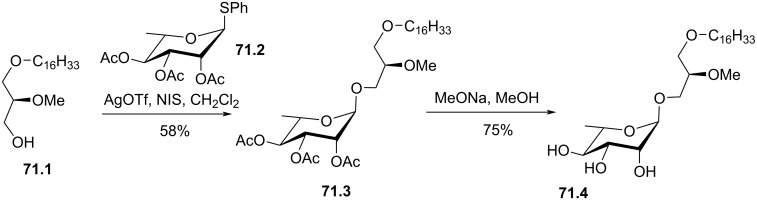
Synthesis of rhamnopyranoside diether **71.4** [196].

The incorporation of a rhamnose moiety in *sn*-3 position of glycerol was reported by F. Schweizer and G. Arthur in 2020 [196]. The glycosylation of the diether **71.1** with phenyl 2,3,4-triacetyl-1-thio-α-ʟ-rhamnopyranoside (**71.2**) in the presence of silver triflate and *N*-iodosuccinimide produced the rhamnopyranoside derivative **71.3**. Deprotection of the alcohol functions produced **71.4** (Figure 71). This neutral ether lipid **71.4** proved to be less toxic against cancer cell lines than other derivatives that included in their structure a protonable amino group.

**Figure 72:**
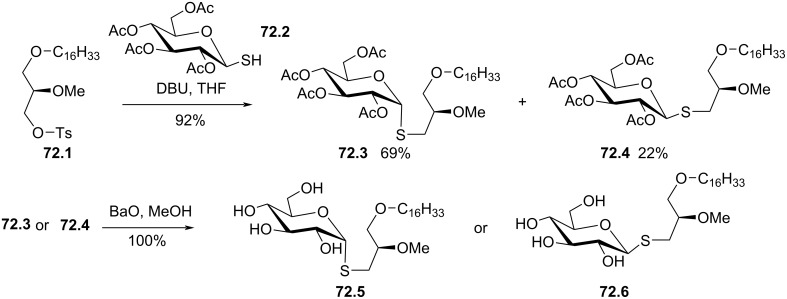
Synthesis of 1-*O*-hexadecyl-2-*O*-methyl-3-*S*-(α-ᴅ-1'-thioglucopyranosyl)-*sn*-glycerol (**72.5**) [225].

With the aim to produce glyco ether lipids featuring higher lipophilicity and possibly higher stability in biological media, R. Bittman et al. reported in 1990 the synthesis of the α-thioglucopyranoside **72.5** (Figure 72) [225]. The glycosylation occurred between the tosylated diether **72.1** and 2,3,4,6-tetra-*O*-acetyl-1-mercapto-β-ᴅ*-*glucopyranose (**72.2**) in the presence of DBU (1,8-diazabicyclo(5.4.0)undec-7-ene) to produce a mixture of the α- and β-thioglycoside **72.3** and **72.4** that were separated by flash chromatography. The deprotection of the alcohol groups of **72.3** was achieved with barium oxide in methanol to give α-thioglycoside **72.5**. The evaluation of **72.5** and **72.6** indicated that **72.5**, used at 40 µM, limited strongly the incorporation of [^3^H]thymidine into DNA of WEHI 3B and C653 cell lines while **72.6** was almost inefficient. However, **72.5** was less efficient than edelfosine.

**Figure 73:**
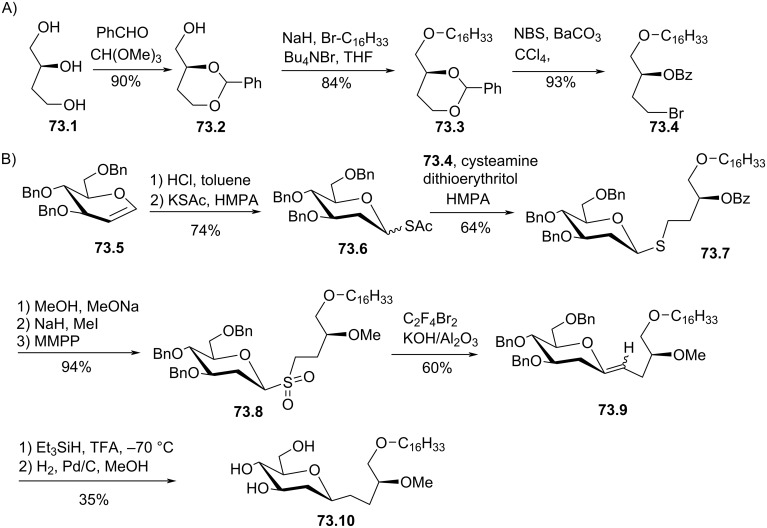
A) Preparation of lipid intermediate **73.4**; B) synthesis of 2-desoxy-*C*-glycoside **73.10** [226].

Still with the aim to produce metabolically stable glyco ether lipids, the groups of R. W. Franck, R. Bittman and G. Arthur reported in 1999 the synthesis of the *C*-glycoside **73.10** that features a 2-desoxyglucopyranoside moiety [226]. The synthesis of the ether lipid fragment (Figure 73A) starts from butanetriol **73.1** that was selectively protected by reaction with benzaldehyde in the presence of orthoformiate to produce the acetal **73.2**. The alkylation of the remaining alcohol function with 1-bromo-hexadecane produced the protected ether lipid **73.3**. The opening of the 1,3-dioxane acetal following the Hanessian–Hullar reaction produced the bromo derivative **73.4** that was used as electrophile in the second part of the synthesis (Figure 73B). The starting saccharide material was the benzylated glucal **73.5** that was hydrohalogenated and subsequently transformed in the thioacetate **73.6**. In situ generation of a thiol intermediate from **73.6** and its reaction with **73.4** produced the 2-desoxythioglucopyranoside **73.7**. The deprotection of the secondary alcohol by trans-esterification with sodium methanolate and the subsequent methylation of the alcoholate allowed the replacement of the benzoyl group by a methoxy group. Then, reaction with magnesium monoperoxyphthalate (MMPP) produced sulfone **73.8**. The application of the Ramberg–Bäcklund reaction that involved dibromotetrafluoroethane and KOH/Al_2_O_3_ lead to the elimination of SO_2_ and the formation of alkene **73.9** as a mixture of diastereoisomers. Finally, ionic hydrogenation reduced the alkene and a subsequent catalytic hydrogenolysis lead to the debenzylated *C*-glycoside **73.10**.

**Figure 74:**
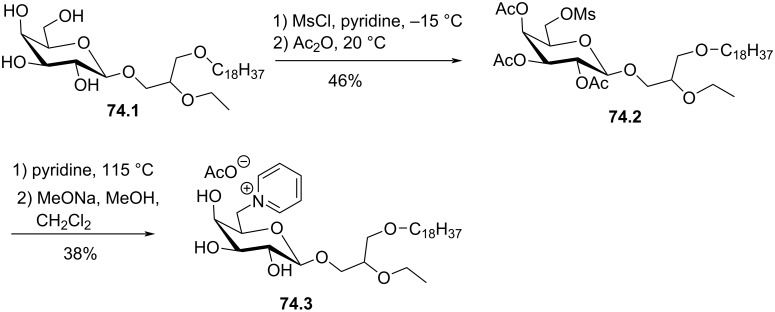
Synthesis of galactose-pyridinium salt **74.3** [228].

N. G. Morozova et al., after reporting the incorporation of a cationic group (imidazolium) between position 3 of glycerol and a saccharide moiety [227], reported in 2019 the functionalization of the position 6 of a saccharide moiety (galactose, mannose) with a cationic group (pyridinium or *N*-methylimidazolium) [228]. The synthesis of the pyridinium derivative is outlined in Figure 74. The synthesis started with the mesylation in position 6 of the galactosyl derivative **74.1**. Then, the pyridinium salt was formed by refluxing **74.2** with pyridine. In the last step the acetyl protecting groups were removed to produce **74.3**.

### 4 Inositol and analogous derivatives of glycerol ether lipids

Phosphoinositides constitute a class of lipids that are involved in a multitude of biochemical processes [229]. These biological roles of phosphoinositides incited chemists and biologists to incorporate phosphatidylinositol or inositol moieties in the structure of glycerol ether lipids.

#### 4.1 Incorporation of an inositol moiety or analogues in position 2 of glycerol

**Figure 75:**
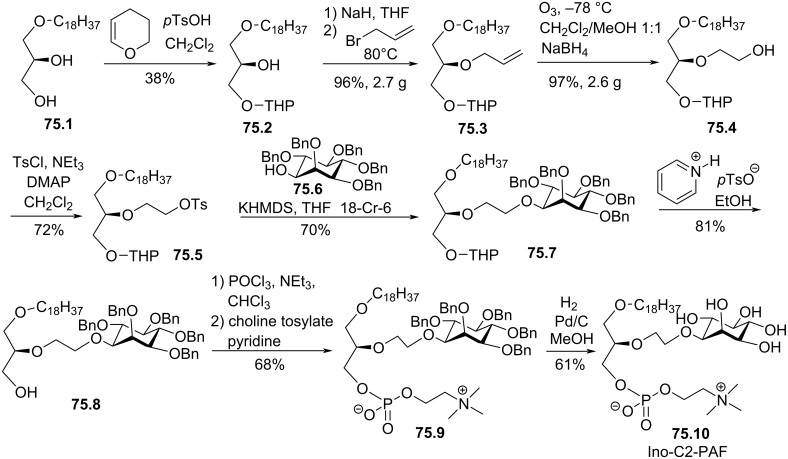
Synthesis of *myo*-inositol derivative Ino-C2-PAF (**75.10**) [230].

K. Danker et al. reported in 2006 the synthesis of one analogue of Glc-PAF in which the glucosyl unit was replaced by a *myo*-inositol moiety (Figure 75) [230]. The synthesis starts from the ether lipid **75.1** which was converted into the tetrahydropyrane protected primary alcohol **75.2**. Then, the allylation of the *sn*-2 position produced **75.3**, which, after a sequence including ozonolyze and reduction, produced **75.4**. This sequence allows the incorporation of an ethylene glycol spacer at the *sn*-2 position. Then, the tosylation of the primary alcohol gave **75.5** and the incorporation of racemic 2,3,4,5,6-penta-*O*-benzylinositol (**75.6**) produced **75.7**. The cleavage of the THP protecting group and the installation of the phosphocholine polar head group produced **75.9**. The debenzylation by catalytic hydrogenolysis produced Ino-C2-PAF (**75.10**). This compound featured a low toxicity (non-toxic up to 5 µM) on keratinocyte cell line (HaCaT cells) but prevent its proliferation.

#### 4.2 Incorporation of an inositol moiety or analogues in position 3 of glycerol

**Figure 76:**
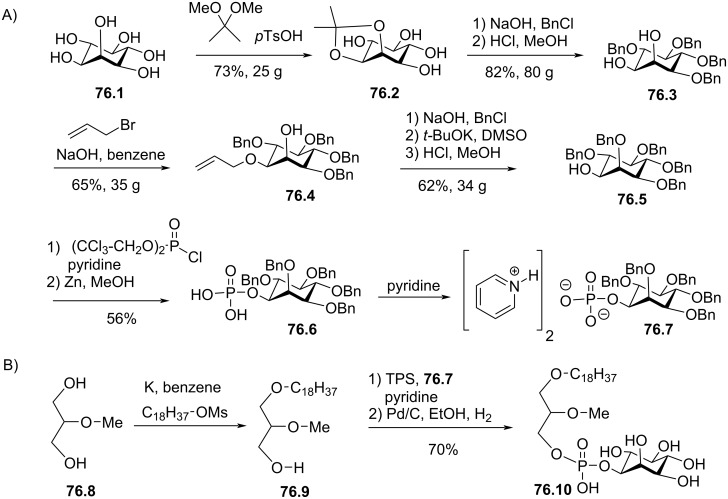
A) Synthesis of *myo*-inositol phosphate building block **76.7**; B) synthesis of *myo*-inositolphosphate diether lipid **76.10** [231].

The groups of K. S. Ishaq and E. J. Modest reported in 1989 the synthesis of analogues of edelfosine in which the choline unit was replaced by a *myo*-inositol moiety (compound **76.10**, Figure 76) [231]. First, the synthesis required to prepare the benzylated phospho-*myo*-inositol **76.7** as reported in Figure 76A. This sequence follows the method initially reported by R. Gigg [232]. *myo*-Inositol was first protected in positions 1 and 2 by reaction with acetone dimethylacetal in acidic conditions to produce **76.2**. Then, the benzylation of the remaining alcohol functions and the deprotection of the acetal in acidic conditions produced the tetrabenzylinositol **76.3**. Then, an allylation reaction proceeds in position 1 selectively to produce **76.4**. The benzylation of the position 2 and the deallylation via an isomerization of the allyl group to a prop-1-enyl moiety with potassium *tert*-butylate followed by hydrolysis in acidic conditions produced pentabenzylinositol **76.5**. The incorporation of the phosphate moiety was achieved with a two-step sequence using first a chlorophosphate and second the deprotection of the trichloroethyl groups with zinc in methanol to produce **76.6** and subsequently its pyridinium salt **76.7**. The synthesis of the ether lipid is reported in Figure 76B. The diol **76.8**, prepared following the method of J. R. Surles et al. [233], was mono-alkylated with an octadecyl lipid chain to produce **76.9**. Then, the coupling of **76.9** with the protected inositol **76.7** in the presence of 2,4,6-triisopropylbenzenesulfonyl chloride (TPS) followed by debenzylation by catalytic hydrogenolysis produced **76.10**. The authors reported that **76.10** inhibited protein kinase C.

**Figure 77:**
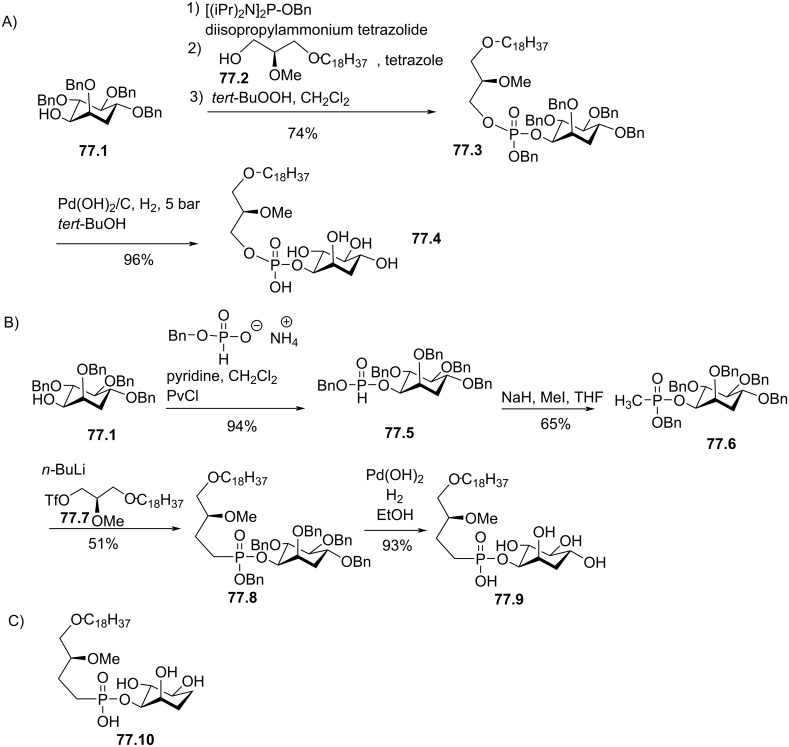
A) Synthesis of phosphatidyl-3-desoxy-inositol **77.4**; B) synthesis of phosphono-3-desoxyinositol **77.9**; C) structure of the analogue 3,4-desoxyinositol **77.10** [234–235].

In 1998, the groups of G. Powis and A. P. Kozikowski reported the synthesis of phosphatidyl-3-desoxyinositol ether lipid **77.4** and its phosphonate analogue **77.9** (Figure 77). The use of the 3-desoxyinositole moiety aimed to develop antiproliferative compounds without affecting the signaling roles of phosphatidyl inositol glycerol lipids [234]. For the preparation of **77.4** (Figure 77A), the reaction started from 2,4,5,6-tetra-*O*-benzyl-3-desoxy-*myo*-inositol (**77.1**) that reacted with *N*,*N*-diisopropyl-*O*-benzylphosphorodiamidite to produce a phosphoramidite as intermediate. This intermediate reacted with diether lipid **77.2** and the oxidation of the phosphite produced phosphate **77.3**. The final step consisted in removing the five benzyl groups by hydrogenolysis catalyzed with palladium hydroxide on charcoal to give **77.4**. For the phosphonate derivative **77.9** (Figure 77B) the synthesis used also **77.1** as substrate. The coupling of **77.1** with the ammonium salt of monobenzylphosphite in the presence of pivaloyl chloride (Pv) produced H-phosphonate **77.5**. Then, its deprotonation with sodium hydride and methylation of the intermediate produced methylphosphonate **77.6**. The deprotonation of **77.6** with butyllithium and the reaction of the intermediate with triflate **77.7** produced diether **77.8**. Finally, the benzyl groups were removed by catalytic hydrogenolysis. It must be noted that the same groups reported in 2000 the synthesis of **77.10** which is an analogue of **77.4** with a 3,4-desoxy-*myo*-inositol moiety as shown in Figure 77C [235]. These groups reported that **77.10** was 18-fold more efficient than **77.4** in the inhibition of PI3-K.

**Figure 78:**
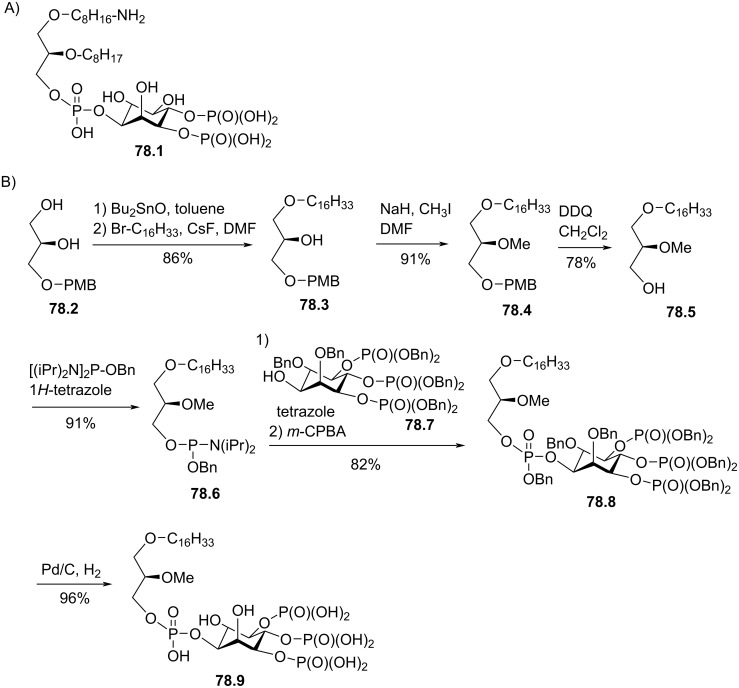
A) Structure of diether phosphatidyl-*myo*-inositol-3,4-diphosphate **78.1**; B) synthesis of phosphatidyl-*myo*-inositol-3,4,5-triphosphate **78.9** [108,236].

The incorporation of a phosphorylated inositol moiety in the structure of ether lipids was reported several times. In 1997, the group of J. R. Falck reported in a communication the synthesis of the diether glycero lipid **78.1** with a phosphatidylinositol diphosphate (PIP2) as polar-head group (Figure 78A) [236]. In 2001, the group of Chen reported in detail the synthesis of phosphatidyl-*myo*-inositol-3,4,5-trisphosphate derivative **78.9** (Figure 78B) [108]. The synthesis started from **78.2** (glycerol protected with a *para*-methoxybenzyl group in position *sn*-3). The incorporation of the hexadecyl lipid chain was achieved by using tin acetal [209] to produce **78.3**. The methylation of the secondary alcohol produced **78.4** and the deprotection of the *sn*-3 alcohol with 2,3-dichloro-5,6-dicyano-*para*-benzoquinone (DDQ) produced alcohol **78.5**. The reaction of **78.5** with *N,N*-diisopropyl-*O*-benzylphosphorodiamidite produced phosphoramidite **78.6**. Its reaction with 2,6-*O,O*-dibenzyl-*myo*-inositol-3,4,5-tris(dibenzylphosphate) **78.7** followed by the oxidation of the phosphite group to a phosphate group produced **78.8**. Finally, the catalytic hydrogenolysis of **78.8** produced the final product **78.9**. The authors reported that the diether **78.9** had a lower affinity for serum proteins compared to its diacyl analogue. **78.9** was also identified for its capacity to stimulate Ca^2+^ influx in T cells.

**Figure 79:**
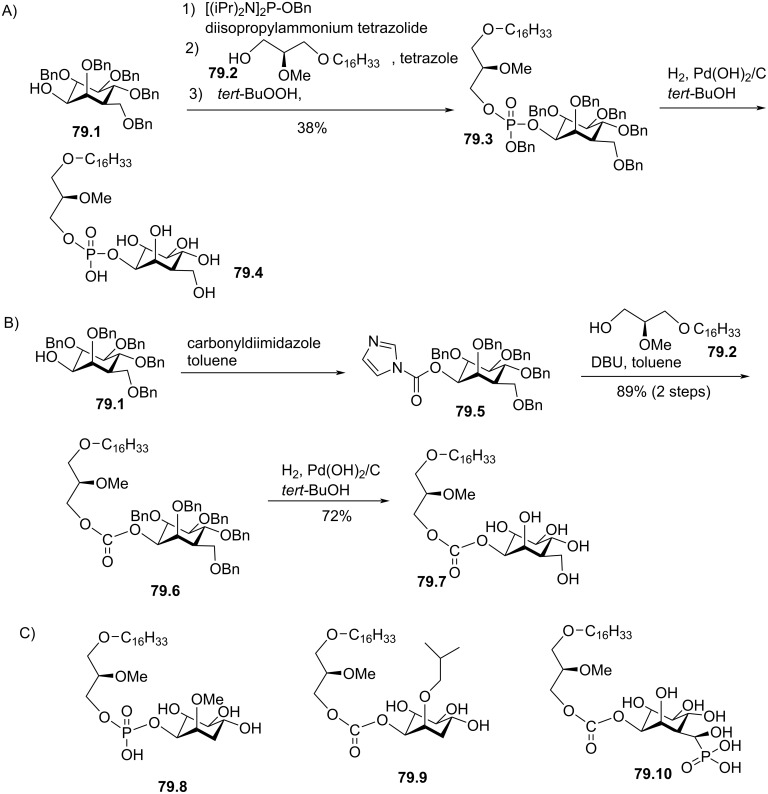
A) Synthesis of diether-phosphatidyl derivative **79.4** featuring a hydroxymethyl group in place of a hydroxy group present in *myo*-inositol; B) synthesis of analogue **79.7** possessing a carbonate moiety in place of the phosphate linkage; C) structure of diether DAG-PI analogues prepared following a similar synthetic scheme [237–239].

In 2000, the group of A. P. Kozikowski reported further modifications on the position 3 of *myo*-inositol by placing a hydroxymethyl group in place of the hydroxy function as illustrated with the structure of **79.4** (Figure 79A) [237]. In the meantime, this group also reported the replacement of phosphate linkage between the diether glycerol unit and the modified *myo*-inositol moiety as illustrated with compound **79.7** (Figure 79B). The synthesis of both compounds **79.4** and **79.7** required first the preparation of the analogue of *myo*-inositol **79.1** as shown in Figure 79. The synthesis of **79.4** used the phosphorodiamidite strategy to link **79.1** to the diether glycerol **79.2**. The final oxidation of the phosphite produced phosphate **79.3**. Finally, the debenzylation is achieved by catalytic hydrogenolysis to produce **79.4**. For the preparation of carbonate **79.7**, the carbonate linker is introduced via the reaction with 1,1’-carbonyldiimidazole to produce in a two-step sequence carbonate **79.6**. The final debenzylation produced the analogue **79.7**. With the aim to improve the metabolic stability of diether-PI analogues, A. P. Kozikowski reported other analogues featuring an alkylation in position 2 of *myo*-inositol as illustrated with **79.8** (Figure 79C) or with an isobutyl group as shown with **79.9** [238]. With the aim to prepare analogues that could mimic 3-phosphorylated phosphatidylinositol, A. P. Kozikowski’s group reported compounds with a hydroxymethylphosphonic acid moiety in position 3 as illustrated with compound **79.10** [239]. The complete biological studies of these diether analogues of diacylglycerolphosphatidylinositol (DAG-PI) were reported in a first study indicating that the desoxy analogues in position 3 and alkylated in position 2 and with a phosphate linker (e.g., **79.8**) were the most efficient compounds to inhibit the serine/threonine kinase Akt [240]. In another study, it was reported that some diether analogues of DAG-PI were able, simultaneously, to inhibit Akt and to activate AMP-activated protein kinase (AMPK) [241].

### 5 Aminoglycoside-based ether lipids

The anticancer properties of glyco-glycero ether lipids invited chemists to modulate the structure of the saccharide moiety leading to the incorporation of aminosaccharides either in position 2 or 3 of glycerol.

#### 5.1 Glycero ether lipids with aminoglycosides in position 2 of glycerol

**Figure 80:**
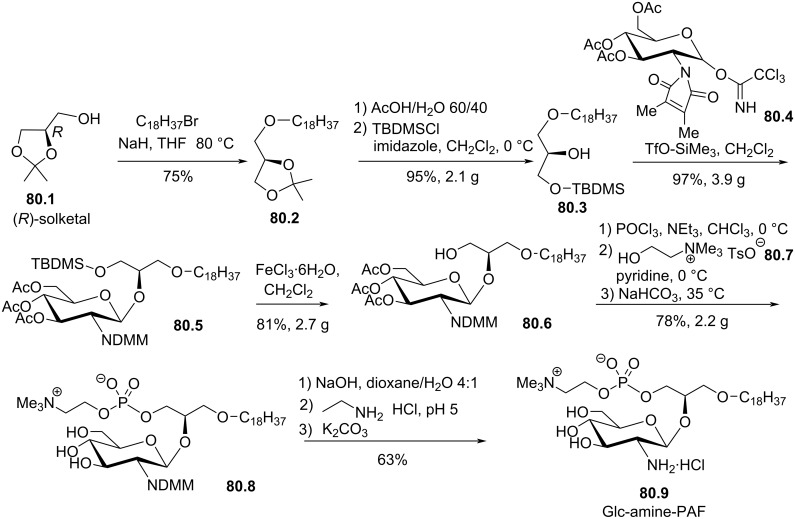
Synthesis of Glc-amine-PAF [78].

The group of K. Danker reported in 2005 the synthesis of glucosamine glycerophospho ether lipids [78]. The synthesis used (*R*)-solketal (**80.1**) as starting material (Figure 80). The incorporation of the lipid chain attached to the glycerol via an ether function produced **80.2**. Then, the acetal was cleaved in acetic acid in the presence of water and the deprotected primary alcohol was protected with *tert*-butyldimethylsilyl chloride (TBDMSCl) to produce **80.3**. The incorporation of the protected glucosamine **80.4** (the amine is protected with dimethylmaleimide (DMM) and the three alcohol functions were acetylated) activated at the anomeric position as tricholoroacetimidate was achieved by using trimethylsilyl trifluoromethanesulfonate as catalyst (TMSOTf; 1 mol %) to produce the intermediate **80.5**. Then, the TBDMS protecting group was removed to produce **80.7**. The authors mentioned some difficulties to remove the TBDMS protecting group by using classical conditions (TBAF or HF-pyridine) and reported that FeCl_3_·H_2_O (2 equivalents) in dichloromethane was efficient to produce **80.6**. The incorporation of the phosphocholine moiety was achieved by using POCl_3_ as phosphorus source and by a successive addition of alcohol **80.7**, choline tosylate (solubilized in a large amount of pyridine) and finally by the hydrolysis of the last P–Cl bond to produce **80.8**. The control of the temperature is essential to avoid the disubstitution of POCl_3_ as it was previously reported for the synthesis of a phosphocholine polar head group [88] or for the design of trisubstituted phosphates [242] or phosphoramidates [243]. Finally, the deprotection of the amine was achieved by the treatment with sodium hydroxide followed by the addition of hydrochloric acid at a controlled pH (pH 5). The final compound Glc-amine-PAF (**80.9**) was isolated in 27% overall yield (135 mg).

#### 5.2 Glycero ether lipids with aminoglycosides in position 3 of glycerol

The incorporation of an aminosaccharide moiety in position 3 of glycerol was more extensively studied than its incorporation in position 2 (last section).

**Figure 81:**
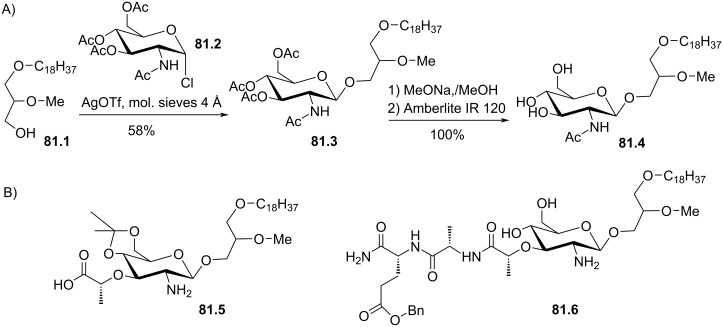
Synthesis of glucosamine ether lipid **81.4** and its analogues functionalized in position 3 of the aminoglycoside moiety [244].

In 1991, S. Adam and F. Kaufmann reported the synthesis of compound **81.4** (Figure 81) [244] with the aim to propose new antiproliferative ether lipids. **81.4** was prepared in two steps from the ether lipid building block **81.1**. First, the glycosilation reaction of **81.1** with the protected glucosamine **81.2** which was activated at the anomeric position was achieved in the presence of silver triflate (Figure 81A). The resulting glyco ether lipid **81.3** was deprotected with sodium methanolate to produce **81.4**. In addition, the authors substituted the aminoglycoside moiety in position 3 by the incorportation of propionic acid and a dipeptide fragment to produce respectivelly **81.5** and **81.6** (Figure 81B). It must be noted that the glycosylation reaction involving **81.1** and **81.2** was also reported by R. Bittman et al. with zinc chloride used as catalyst [245]. The β-stereoisomer was formed, however, the addition of trityl chloride and a longer reaction time (36 h) produced the α-stereoisomer. This result indicates that the β-isomer is formed under kinetic control and the α-isomer corresponds to the thermodynamic product.

**Figure 82:**
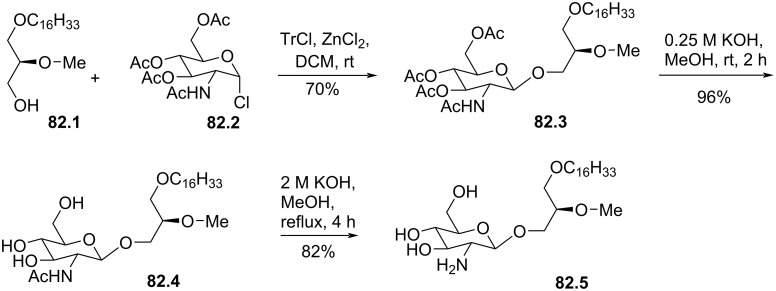
Synthesis of fully deprotected aminoglucoside ether lipid **82.5** [246].

In 1996 the group of Arthur and Bittman reported the synthesis of the fully deprotected aminoglycosyl ether lipid **82.5** (Figure 82) [246]. The glycosylation reaction involving **82.1** and **82.2** was achieved in the presence of zinc chloride and trityl chloride used in stoichiometric quantities. Then, the deprotection of acetyl ester functions was achieved with KOH in methanol at 20 °C to produce **82.4**. Finally, the deprotection of the primary amine was achieved with KOH in refluxed methanol solution to produce **82.5**. In 2011, the same group reported more hydrophobic analogues of **82.5** featuring an *N*-benzylated amine prepared by reductive amination of **82.5** in methanol [247]. In another study, the same group reported that **82.5** induced the formation of cytoplasmic acidic vacuoles [248]. They have also reported that **82.5** induced cell death by a mechanism independent of autophagy and caspase activation. **82.5** is likely to induce cell death via a permeabilization of lysosomal membrane leading to release hydrolases into the cytosol [249].

**Figure 83:**
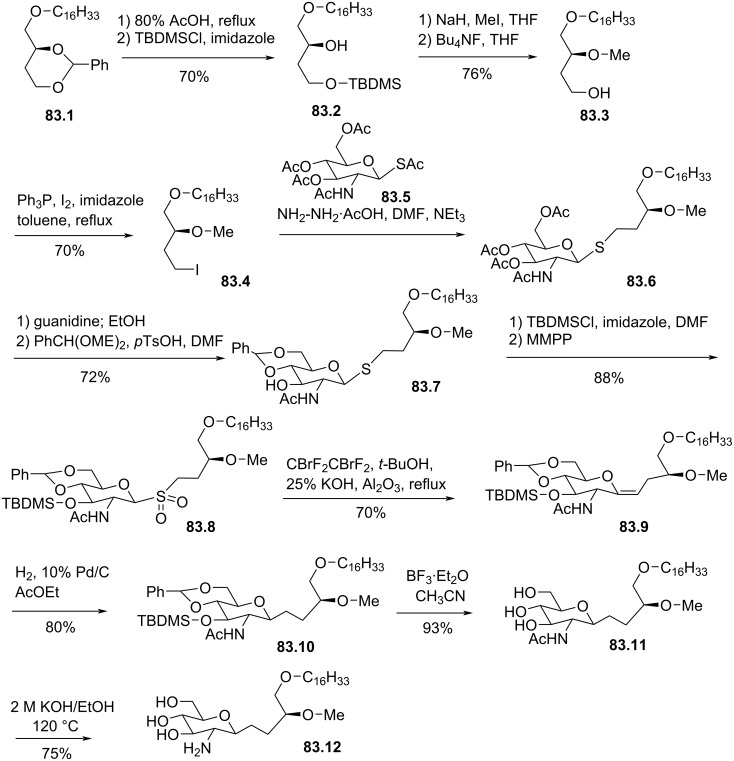
Synthesis of *C*-aminoglycoside **83.12** using Ramberg–Bäcklund rearrangement as a key step [250].

With the aim to reduce the degradation of aminoglycosides (e.g., **82.5**) with glucosidase, the synthesis of *S*- and *C*-glycosides was reported by the group of Bittman and Arthur (Figure 83) [250]. The synthesis started with the synthesis of the iodo ether lipid **83.4** from the protected ether lipid **83.1** that was prepared from 1,2,4-butanetriol [226]. The deprotection of **83.1** under acidic conditions followed by the silylation of the primary alcohol with TBMSCl produced **83.2**. Then, methylation of the secondary alcohol and deprotection of the primary alcohol produced **83.3**. The application of the Appel reaction in the presence of triphenylphosphine and iodine produced **83.4**. The selective deacetylation of the thioacetyl group of **83.5** in the presence of hydrazine, produced in situ a thiol that was used as nucleophilic species to react with iodide **83.4** to produce the thioglycoside **83.6**. The key step for the preparation of the *C*-glycoside involved a Ramberg–Bäcklund rearrangement of a sulfone that requires KOH. These reaction conditions were not compatible with the presence of the acetyl groups on the glucosamine moiety. Accordingly, the protecting groups were modified. First, the acetyl ester protecting groups were removed by reaction of guanidine in ethanol. The 4,6-diol was protected by *trans*-acetalization using benzylidene acetal to produce **83.7**. The remaining alcohol function was protected with TBDMS and the thioglycoside was oxidized to a sulfone with magnesium monoperoxyphthalate (MMPP) to produce **83.8**. The application of the Ramberg–Bäcklund rearrangement was achieved in the presence of dibromotetrafluoroethane as halogenating agent. The alkene **83.9** (*Z*-stereoisomer only) was hydrogenated and then the protecting groups were removed in two steps to produce **83.12**.

**Figure 84:**
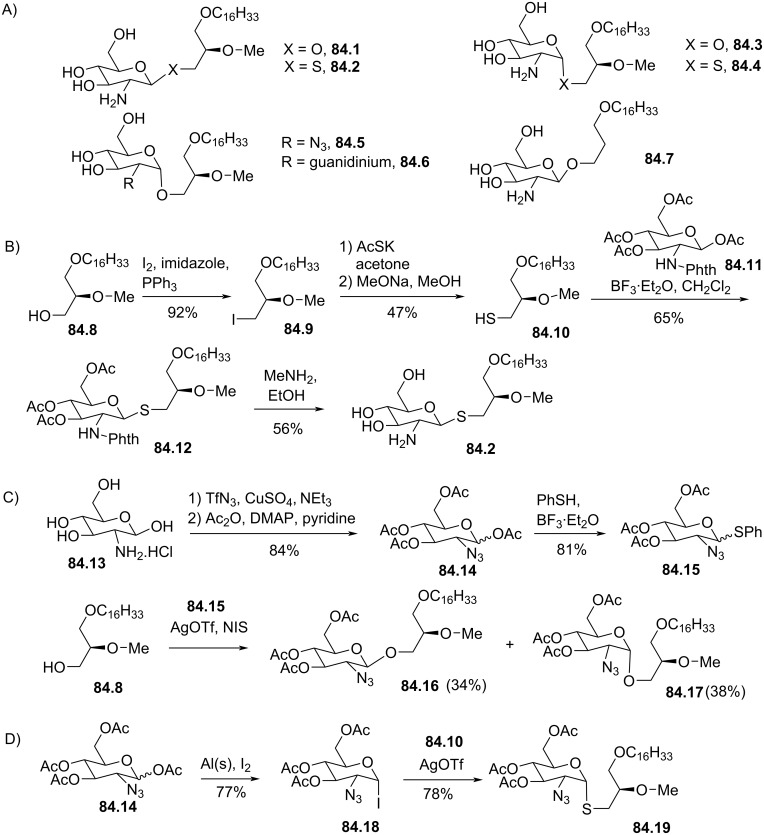
A) List of the most important glyco lipids and amino glyco lipids included in the study of Arthur and Schweizer; B) synthesis of the β-amino-thioglycoside **84.2**; C) synthesis of the key intermediates **84.16** and **84.17** for the preparation of the α-stereoisomers **84.3**, **84.5**, **84.6**; D) synthesis of the precursor **84.19** for the synthesis of the α-aminothioglycoside **84.4** [251].

In 2013, the groups of Arthur and Schweizer studied the role of the anomeric linkage, the cationic charge and the glycerol moiety on the antitumor activity of glyco glycero ether lipids [251]. In this study they have included the aminoglycoside **84.1** (β-anomer) and its thio-analogue **84.2**, the α-aminoglycoside **84.3** and its thio-analogue **84.4**, the α-glycoside with an azido group **84.5** or a guanidinium moiety **84.6** in position 3 of the saccharide unit and an analogue without the glycerol moiety **84.7** (Figure 84A). The thio-analogue **84.2** was prepared from the diether **84.8** (Figure 84B). The Appel reaction produced the iodide **84.9**. Then, the thiol **84.10** was prepared in two steps using potassium thioacetate as source of sulfur. The glycosylation reaction of **84.10** with glucosamine **84.11** protected on the amine with a phthalimide group and with four acetyl groups on the alcohol functions produced the protected aminothioglycoside **84.12**. Then, the deprotection of both the alcohol and primary amine was achieved with methylamine in ethanol to produce **84.2** (Figure 84B). The α-glycosides **84.4**, **84.5**, and **84.6** were prepared from glucosamine hydrochloride **84.13** (Figure 84C). The introduction of the azido group was achieved by an adaptation of the method initially reported by Vasella et al. [252], to produce, after protecting the alcohol function with acetyl groups, the azido derivative **84.14**. The activation of the anomeric position with thiophenol produced **84.15** that was engaged in the glycosylation reaction with alcohol **84.8** and catalyzed with silver triflate. The two diastereoisomers **84.16** and **84.17** were separated and **84.17** was used as key intermediate for the preparation of compounds **84.3**, **84.5**, and **84.6**. The α-thioglycoside was prepared from azido derivative **84.14** (Figure 84D). First, iodination of the anomeric position in the presence of aluminum and iodine [253] produced glycosyl iodide **84.18**. Then, the glycosylation reaction involving the thiol **84.10** and **84.18** in the presence of silver triflate produced exclusively the α-azidothioglycoside **84.19**. This compound was the precursor of compound **84.4**. Of note, the glycosylation reaction involving thiol **84.10** and the azidoglucoside activated in the anomeric position with a trichloroacetimidate was also reported by Schweizer and Arthur [254]. In that case a mixture of α- and β-stereoisomers was isolated and separated to produce both anomers of amino-thioglycoside **84.2** and **84.4**.

**Figure 85:**
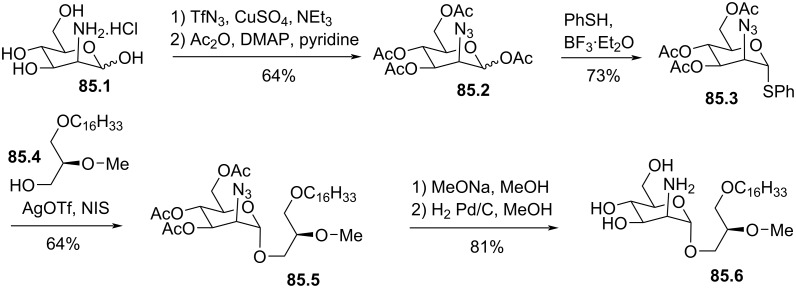
Synthesis of mannosamine ether lipid **85.6** [254].

In 2014 the group of Schweizer and Arthur reported the synthesis of mannosamine ether lipid **85.6** (Figure 85) [254]. The synthesis starts with the replacement of the amine group by an azido group using azide triflate as reagent. Then, the acetylation of the alcohol function produced **85.2**. The activation of the anomeric position with thiophenol in the presence of Lewis acid produced **85.3** as a pure anomer (α). The glycosylation reaction involving the ether alcohol **85.4** and **85.3** in the presence of silver triflate produced **85.5**. The deprotection of the alcohol function and the reduction of the azide group into an amine produced the mannosamine ether lipid **85.6**. It was observed that **85.6** had a weak effect on the viability of different epithelial cancer cell lines.

**Figure 86:**
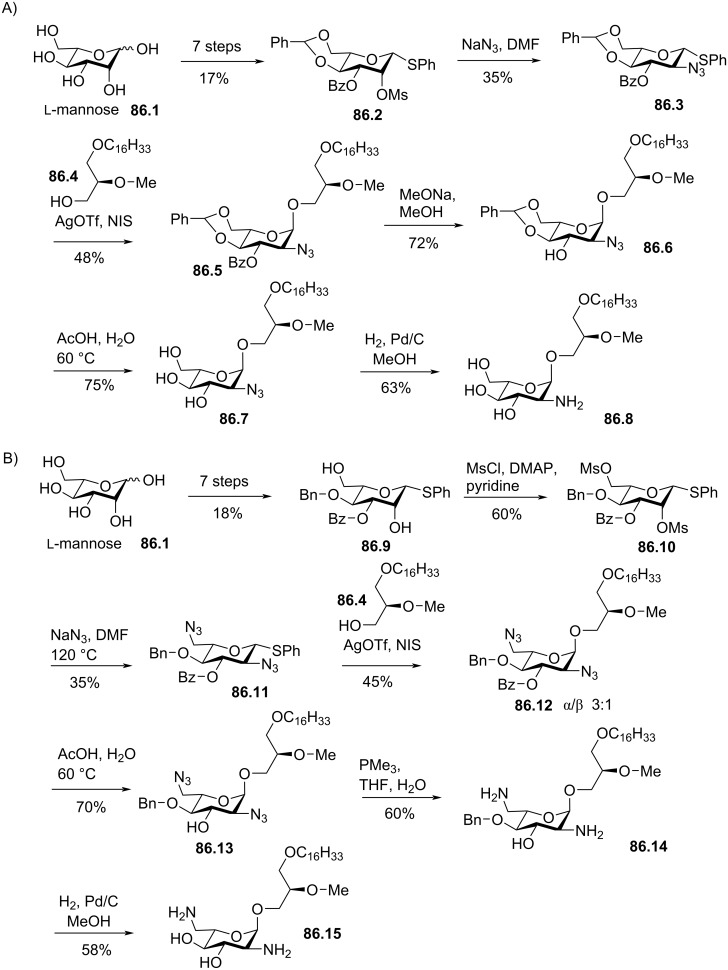
A) Synthesis of glucosamine ether lipids with a non-natural ʟ-glucosamine moiety; B) synthesis of ether lipids with a 2,6-diamino-ʟ-glucosyl moiety [255].

With the aim to avoid the degradation of glycoside ether lipids that can occur in vivo with glucosidases, the groups of Arthur and Schweizer reported the synthesis of glucosamine ether lipid derivatives featuring the non-natural ʟ-glucosamine (Figure 86A) [255]. The synthesis started from ʟ-mannose (**86.1**) that was transformed into **86.2** after 7 steps (17% yield). Then, **86.2** reacted with sodium azide to produce **86.3**. The glycosylation reaction of **86.3** with **86.4** in the presence of silver triflate produced the intermediate **86.5**. Then, the deprotection of the benzoyl group with methanolate produced **86.6**. An acidic treatment removed the acetal to produce **86.7**. Finally, the reduction of the azide moiety by hydrogenolysis catalyzed with palladium on charcoal produced **86.8**. The authors also reported the synthesis of analogues of **86.8** having on the ʟ-glucosamine moiety a second amino group (Figure 86B). The intermediate **86.9** was prepared from ʟ-mannose in 7 steps (18% yield). **86.9** was mesylated in positions 2 and 6 to produce **86.10**. The incorporation of two azide groups was obtained by reaction of **86.10** with sodium azide to produce **86.11**. Then, the glycosylation reaction in the presence of **86.4** and silver triflate produced a mixture of anomers. The anomer α-**86.12** was separated and engaged in a deprotection of the benzoyl group to produce **86.13**. Then, the Staudinger reaction with trimethylphosphine produced the diamino derivative **86.14**. Finally, the debenzylation of the protected alcohol was achieved by hydrogenolysis catalyzed by palladium on charcoal to produce **86.15**. The authors reported that **86.14** was one of the most efficient compounds to kill cancer stem cells in this series of compounds. The mechanism of action does not involve apoptosis pathways nor a membrolytic effect. Some results showing cytoplasmic vacuolization suggest cell death by methuosis.

**Figure 87:**
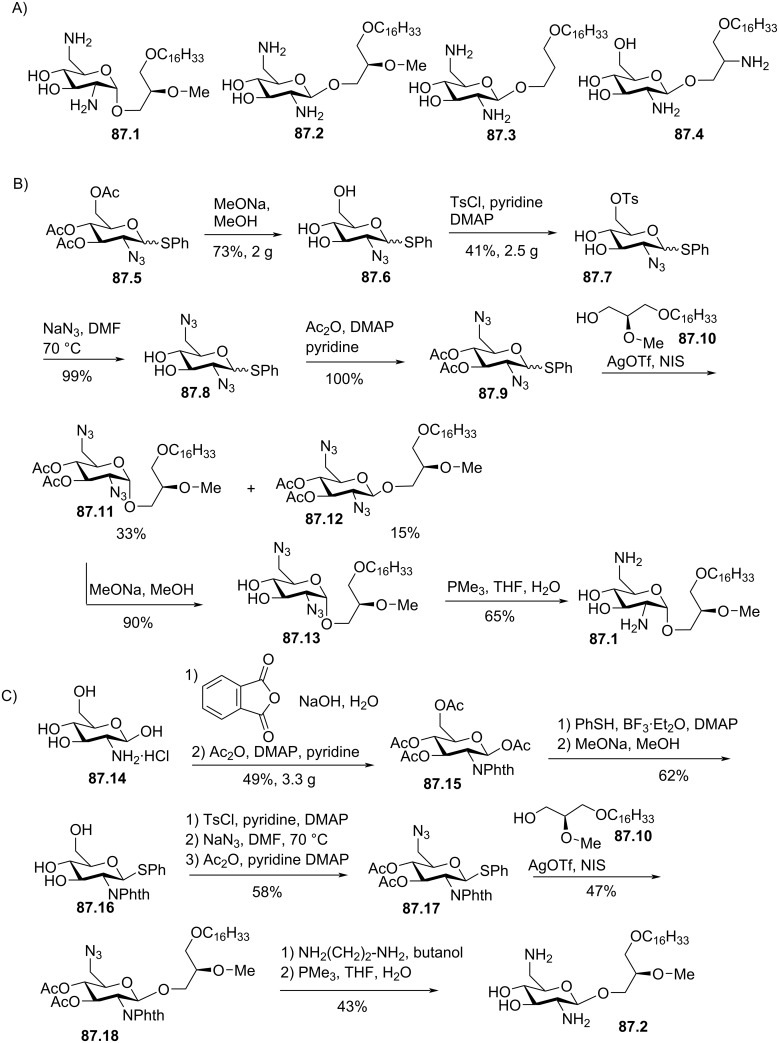
A) Structure of the most efficient anticancer agents **87.1**–**87.4** featuring a diamino glyco ether lipid structure; B) synthesis of the α-diamino glyco ether lipid **87.1**; C) synthesis of the β-diamino glyco ether lipid **87.2** [256].

**Figure 88:**
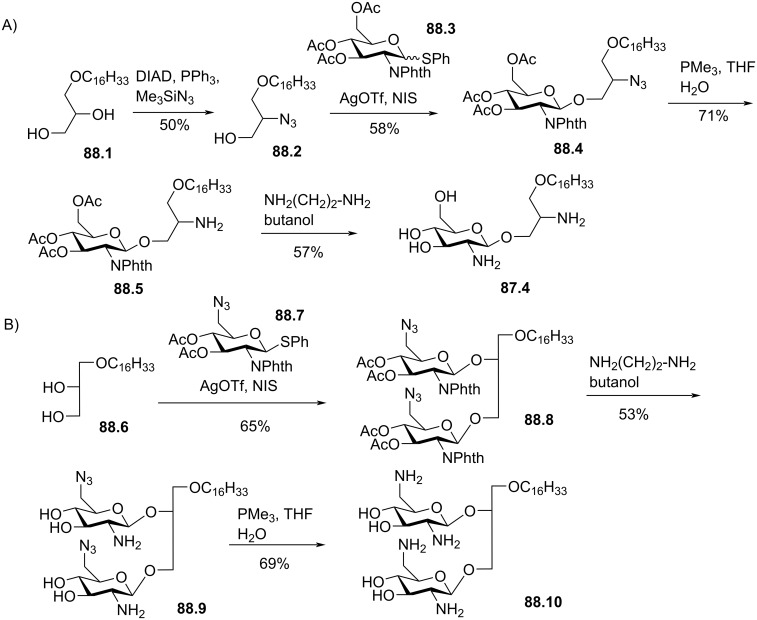
A) Synthesis of diamino glyco ether lipid **87.4**; B) synthesis of bis-glycosylated ether lipid **88.10** [256].

In a series of diamino ether lipids, the derivatives **87.1**–**87.4** (Figure 87A) were identified by the group of Arthur and Schweizer as the most efficient anticancer agents against several epithelial cancer cells [256]. The synthesis of **87.1** is reported in Figure 87B. Glucosamine was transformed in compound **87.5** in 3 steps following a reported methodology [251]. Then, **87.5** was deacetylated to produce **87.6**. The primary alcohol was selectively tosylated to produce **87.7** and then, the azido group was introduced to give the diazide derivative **87.8**. The two remaining unprotected primary alcohol were then acetylated to give **87.9**. The glycosylation reaction was achieved by reaction of **87.9** with **87.10** in the presence of silver triflate and *N*-iodosuccinimide (NIS). A mixture of anomers was formed and the α-anomer **87.11** was isolated in 33% yield. The deacetylation produced **87.13**. Then, the Staudinger reaction was applied in the presence of trimethylphosphine to produce **87.1**. For the synthesis of the β-anomer **87.2**, the strategy was different (Figure 87C). Starting from glucosamine hydrochloride **87.14**, the amine was protected as naphthalimide (NPhth) and then, the alcohol functions were acetylated to produce the fully protected intermediate **87.15**. The introduction of the phenylthiol moiety at the anomeric position followed by removing the acetyl protecting groups produced **87.16**. A three-step sequence (tosylation, azidation, acetylation) allowed to introduce an azido group in place of the primary alcohol and to protect the two remaining alcohol functions as acetyl esters **87.17**. The glycosylation reaction with **87.10** produced the β-anomer **87.18** in 47% yield. Then, the deprotection of the amine and alcohols was achieved with ethylenediamine in butanol and the two azido groups were converted in an amine under Staudinger conditions to produce **87.2**. **87.3** was synthesized following the same methods as used for the synthesis of **87.2**. The synthesis of **87.4** is detailed in Figure 88A. The synthesis started by a Mitsunobu reaction using diisopropylazodicarboxylate (DIAD), triphenylphosphine and trimethylsilylazide as nucleophile. As previously reported by Bittman et al., this Mitsunobu reaction applied to 1,2-diols is regioselective in favor of the formation of 2-azido-1-ol [257]. The application of these conditions to compound **88.1** produced the 2-azido derivative **88.2** in 50% yield. Then, the glycosylation reaction with **88.3** in the presence of silver triflate and *N*-iodosuccinimide produced the glyco ether lipid **88.4**. The reduction of the azido group to an amino group under Staudinger conditions produced **88.5**. Then, the alcohol and amino groups were deprotected with ethylenediamine in butanol to produce **87.4**.

A double glycosylation of the glycerol ether lipid **88.6** with the donor **88.7** was also reported (Figure 88B). The resulting product **88.8** was deprotected with ethylenediamine to produce **88.9**. Then, the two azido groups were reduced with trimethylphosphine to produce the tetraamino derivative **88.10**. This compound **88.10**, likely due to its higher hydrophilic properties that could reduce cell absorption, proved to be less toxic for cancer cells when compared to compounds **87.2** and **87.3** [256].

A similar double glycosylation but with mono-amino glycoside was also reported by Arthur and Schweizer [258]. The singularity of the biological action of this class of compounds (amino glycoside ether lipids) that kill cancer cells by methuosis (apoptosis independent pathway) was recently reviewed [259].

**Figure 89:**
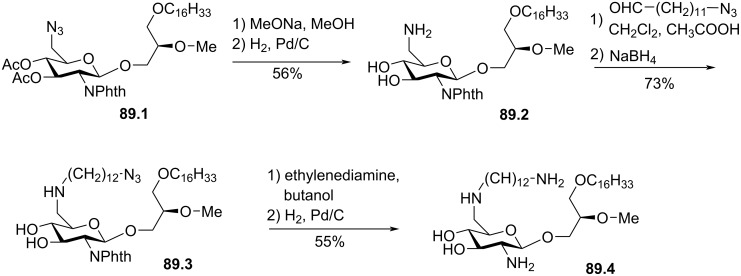
Synthesis of triamino ether lipid **89.4** [260].

The incorporation of a third amino function within the structure of mono-amino-glycoside ether lipids was reported by the groups of Arthur and Schweizer (Figure 89) [260]. **89.1** that was previously reported [258] was used as starting material. The two acetylated alcohol functions were deprotected by *trans*-esterification using methanolate as nucleophile. Then, the reduction of the azido group by palladium-catalyzed hydrogenation produced **89.2** (Figure 89). The reductive amination of **89.2** with 12-azidododecanal produced **89.3**. The deprotection of the primary amine protected as naphthalimide (NPhth) was achieved with ethylenediamine in butanol. Then, the azido group was reduced to a primary amine by palladium hydrogenation to give the final compound **89.4**. **89.4** was identified as the most efficient compound in a series of 8 compounds that were evaluated for their cytotoxicity on 6 human epithelial cancer cell lines. Noteworthy, **89.4** was more efficient than doxorubicin and cisplatin against BT474 cancer stem cells and was also very efficient against triple negative breast cancer (TNBC) cell lines. In vitro experiments revealed that **89.4** did not activate caspase nor had a membranolytic mode of action.

**Figure 90:**
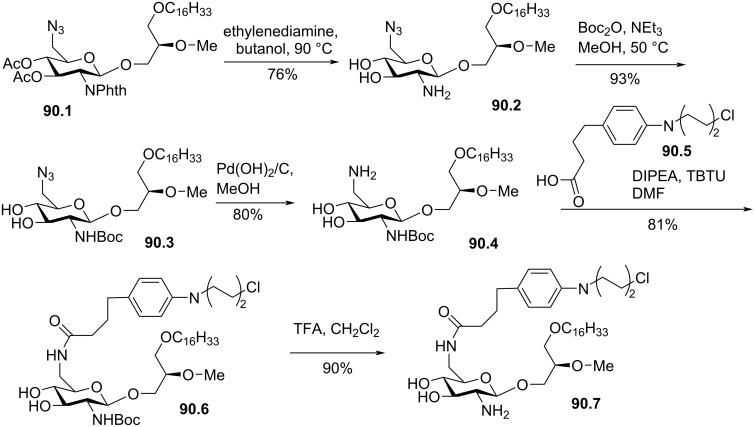
Synthesis of chlorambucil conjugate **90.7** [261].

The groups of G. Arthur and F. Schweizer also reported the synthesis of amino-glycosyl ether lipids conjugated with chlorambucil which is an alkylating agent [261]. The conjugation was achieved in positions 2 or 6 of the 2,6-diaminoglucopyranoside moiety via an amide linkage. An illustration of this conjugation in position 6 is depicted in Figure 90. Starting from **90.1**, the primary amine was deprotected with ethylenediamine in butanol to produce **90.2**. Then, the primary amine was protected as a *tert*-butoxycarbamate (BOC) to produce **90.3**. The hydrogenation of the azide function produced the amine **90.4** that was engaged in an amidation reaction with chlorambucil (**90.5**) in the presence of DIPEA, TBTU in dimethylformamide to produce **90.6**. In the last step, the BOC protecting group was removed with trifluoroacetic acid (TFA) to produce **90.7**. The viability of six epithelial cancer cells (e.g., MDA-MB-231, PC3) was evaluated in the presence of **90.7**. The authors reported that **90.7** was more cytotoxic than the same compound which lack the chlorambucil moiety.

### 6 Summary of the main synthesis approaches

This review reports the synthesis of a multitude of ether lipids featuring different lipid chains, polar head groups and possessing a glycerol unit or molecular fragment mimicking it. Each synthesis is singular but some synthesis steps and synthesis strategies are common and some are more frequently employed. In this section we propose to summarize first the main strategies to prepare the glycero lipid building blocks and then the methods used to introduce the polar head groups (e.g., phosphocholine or a saccharide unit).

**Figure 91:**
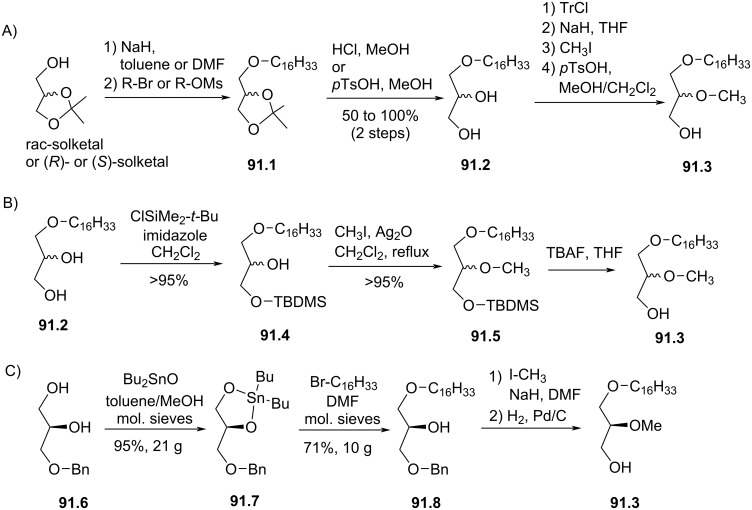
Three main methods for the preparation of glycerol ether lipid **91.3**; A) from solketal and via a tritylation step; B) by using TBDMS as protecting group and a methylation step using silver oxide; C) by using dibutyltin oxide.

As depicted in Figure 91A, one of the most frequently used sequence starts from solketal (racemic or chiral). The lipid chain (here a palmitic lipid chain) was first introduced and the two alcohol functions are deprotected under acidic conditions. Then, the selective protection of the primary alcohol can be achieved with trityl chloride in pyridine [139] or in dichloromethane in the presence of DMAP [189]. The methylation of the secondary alcohol followed by the deprotection of the trityl group produced the key intermediate **91.3**. The limitation of this approach can come from the stability of the trityl group when the intermediate compound must be purified by chromatography on silica gel. An alternative strategy (Figure 91B) uses *tert*-butyldimethylsilyl (TBDMS) as protecting group. The methylation of the secondary alcohol is also readily alkylated by using iodomethane in the presence of Ag_2_O [215]. Another strategy uses dibutyltin oxide to produce the tin acetal **91.7** as intermediate. Interestingly, this acetal can be regioselectively alkylated with a lipid chain to produce, at a multi-gram scale, the lipid glycerol derivative **91.8** [209]. This efficient method was globally less employed. This is likely explained by the use of tin species that are known to be difficult to remove completely from the final compounds.

**Figure 92:**
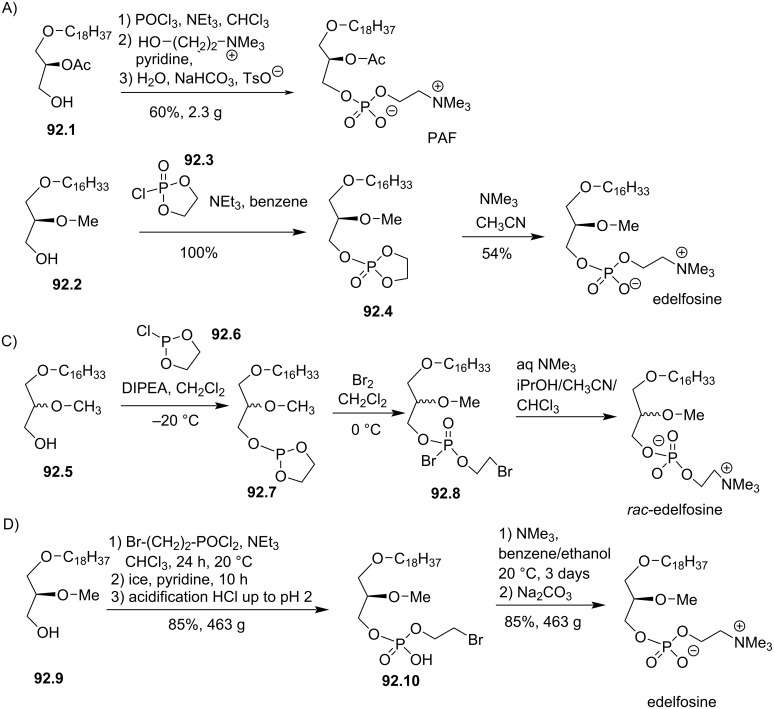
Four different methods for the installation of the phosphocholine polar head group; A) method using POCl_3_ and choline tosylate; B) method using 2-chloro-2-oxo-1,2,3-dioxaphospholane; C) method using a cyclic chlorophosphite; D) method using 2-bromoethyl phosphorodichloridate.

For the installation of a phosphocholine polar head group four classical methods are presented in Figure 92. The first method uses POCl_3_ as electrophile followed by the reaction with choline tosylate in the presence of pyridine (Figure 92A). This reaction was applied to the preparation of PAF [71]. The difficulty with this method comes from the sensitivity of POCl_3_ with water and from the low solubility of choline tosylate. An alternative method that involved well soluble reagents uses 2-chloro-2-oxo-1,2,3-dioxaphospholane (**92.3**, Figure 92B). The intermediate **92.4** is readily opened thanks to the nucleophilicity of trimethylamine. This method was applied for the preparation of edelfosine [118]. An alternative of this method uses 2-chloro-1,3,2-dioxaphospholane (**92.6**). The bromination of the intermediate **92.7** produces the dibromo derivative **92.8** that was converted into racemic edelfosine after a reaction with trimethylamine (Figure 92C) [119]. Finally, the use of 2-bromoethyl phosphorodichloridate produces the monobromo derivative **92.10** that was subsequently transformed in edelfosine (Figure 92D) [116]. It must be noted that all these methods implied water sensitive reagents and the purification of the final compounds is not so easy.

**Figure 93:**
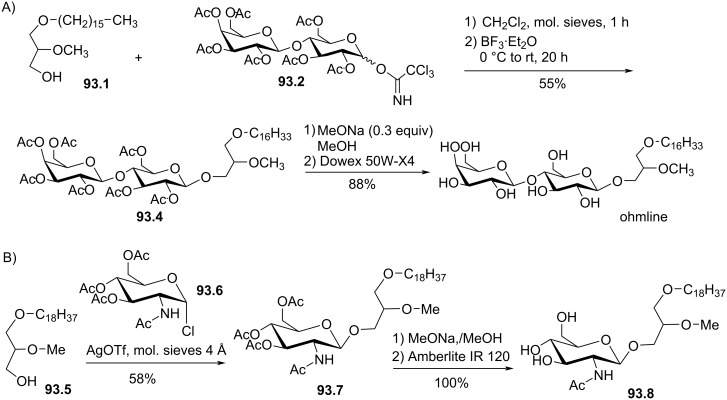
Illustration of two methods for the installation of saccharides or aminosaccharides; A) O-glycosylation reaction used to prepare ohmline; B) O-glycosylation involving protected chloroglucosamine.

Most recent works have revealed the interest to replace the phosphocholine polar head group by a saccharide moiety or an aminosaccharide group. Two examples of the incorporation of a saccharide polar head group are illustrated in Figure 93. For the incorporation of a lactose unit (a monosaccharide group can be introduced according to the same methodology), the O-glycosylation reaction involves the alcohol **93.1** and the protected lactose **93.2** activated at the anomeric position with a trichloroacetimidate (Schmidt’s method) [262]. This protocol was applied to prepare ohmline [41]. For the incorporation of the aminoglycoside moiety, a classical method for the O-glycosylation consists to engage the protected chloroglucosamine **93.6** in the presence of Lewis acid. This sequence was used to prepare compound **93.8** [244].

## Conclusion

Ether lipids constitute a class of natural compounds that are present in many human organs and their distribution/expression is altered in some pathological diseases (e.g., cancer). The exact roles of this class of lipids is not yet fully understood and the diversity of their structure due to the possible variation of the lipid chains present in *sn*-1 and *sn*-2 might influence their biological roles. The progress in analytical methods to determine more accurately the tissues distribution of each type of ether lipids is needed to correlate their structure on the physiological or pathological effect of this class of compounds. Beside the need to better understand the biological role of ether lipids, the development of synthetic analogues of ether lipids was developed thanks to organic chemists and biologists. This type of research, developed jointly by academics and private companies focused on the introduction of a second ether function in position *sn*-2 of glycerol in order to produce more biologically stable compound analogues of PAF. The identification of the anticancer effect of edelfosine invited to propose new structures and to develop new synthetic methodologies to produce ether lipid analogues. It must be noted that the initial works of H. Eibl, O. Westphal, and I. Benveniste followed by the important contributions of the groups of R. Bittman, G. Arthur and F. Schweizer were essential to this field of research. The works aiming to decipher the mechanism of action of edelfosine illustrated by the key contributions of the group of F. Mollinedo. This group demonstrated that edelfosine is a pro-apoptotic agent and they also demonstrated the role of the lipid raft in edelfosine action [263] that opened the way to lipid raft-targeted therapy [264]. More recent developments of ether lipids were based on one side by the synthesis of non-toxic analogues of edelfosine by the replacement of the phosphocholine moiety by a saccharide or disaccharide unit (ohmline). The group of C. Vandier brought a decisive contribution by demonstrating the effect of edelfosine and ohmline as modulators of the SK3 ion channel and having an influence on SK3-dependent cancer cell migration encountered in some cancer cells. The capacity of ohmline to modulate selectively the activity of some membrane proteins opens new perspectives of development that still require further investigations to determine the origin of this selectivity and the accurate mechanism of action. To this regard, the group of C. Vandier and we have reported that the chirality of ohmline at the *sn*-2 position of the glycerol unit had no effect on the modulation of the SK3 function [41]. In addition, we have reported that ohmline modulates the biophysics of model plasma membranes suggesting that ohmline could exert its effect not by a direct interaction with the SK3 protein but by modulating the lipid environment or the dynamics of the lipid environment of membrane proteins [218]. According to this mechanism, it is likely that cholesterol could act as a molecular relay. The selectivity of action of ohmline which is now well characterized (e.g., no effect on SK2) could be explained by the different interactions of the membrane proteins with its lipid environment. Another important and recent development was the development of new analogues of edelfosine featuring an aminoglycoside of the poly-aminoglycoside moiety (GAEL) as polar head group instead of the phosphocholine moiety. These compounds in contrast to ohmline feature cancer cell toxicity thus highlighting the effect of the amino groups on cell toxicity. Some derivatives feature remarkable toxicity on different epithelial cancer cells. The mechanism of action of this type of compounds recently studied by the groups of G. Arthur and F. Schweizer, suggest that this type of compounds kill cancer cells via an apoptosis-independent mechanism and caspase-independent cell death. These authors demonstrate that endocytosis is required to observed toxicity. The action of GAELs could induce cancer cell death via methuosis. These results also contribute to demonstrate the interest of this class of compounds and emphasize the need of additional studies to evaluate the therapeutic usefulness of this type of compounds.

This review, which is focused on the synthetic methodologies used to prepare ether lipids, aims to offer an overview on the different methods used to prepare ether lipids with structural variations on the polar head groups, on the glycerol moiety or on the lipid chains. The most recent results emphasize the original mechanism of action of some ether lipid derivatives and also their selective effects on some membrane proteins. These results must invite to additional studies that will likely involve the synthesis of new ether lipid derivatives.
